# Body size and intracranial volume interact with the structure of the central nervous system: A multi-center in vivo neuroimaging study

**DOI:** 10.1162/imag_a_00559

**Published:** 2025-05-07

**Authors:** René Labounek, Monica T. Bondy, Amy L. Paulson, Sandrine Bédard, Mihael Abramovic, Eva Alonso-Ortiz, Nicole T. Atcheson, Laura R. Barlow, Robert L. Barry, Markus Barth, Marco Battiston, Christian Büchel, Matthew D. Budde, Virginie Callot, Anna Combes, Benjamin De Leener, Maxime Descoteaux, Paulo Loureiro de Sousa, Marek Dostál, Julien Doyon, Adam V. Dvorak, Falk Eippert, Karla R. Epperson, Kevin S. Epperson, Patrick Freund, Jürgen Finsterbusch, Alexandru Foias, Michela Fratini, Issei Fukunaga, Claudia A.M. Gandini Wheeler-Kingshott, GianCarlo Germani, Guillaume Gilbert, Federico Giove, Francesco Grussu, Akifumi Hagiwara, Pierre-Gilles Henry, Tomáš Horák, Masaaki Hori, James M. Joers, Kouhei Kamiya, Haleh Karbasforoushan, Miloš Keřkovský, Ali Khatibi, Joo-won Kim, Nawal Kinany, Hagen Kitzler, Shannon Kolind, Yazhuo Kong, Petr Kudlička, Paul Kuntke, Nyoman D. Kurniawan, Slawomir Kusmia, Maria Marcella Laganà, Cornelia Laule, Christine S.W. Law, Tobias Leutritz, Yaou Liu, Sara Llufriu, Sean Mackey, Allan R. Martin, Eloy Martinez-Heras, Loan Mattera, Kristin P. O’Grady, Nico Papinutto, Daniel Papp, Deborah Pareto, Todd B. Parrish, Anna Pichiecchio, Ferran Prados, Àlex Rovira, Marc J. Ruitenberg, Rebecca S. Samson, Giovanni Savini, Maryam Seif, Alan C. Seifert, Alex K. Smith, Seth A. Smith, Zachary A. Smith, Elisabeth Solana, Yuichi Suzuki, George W Tackley, Alexandra Tinnermann, Jan Valošek, Dimitri Van De Ville, Marios C. Yiannakas, Kenneth A. Weber II, Nikolaus Weiskopf, Richard G. Wise, Patrik O. Wyss, Junqian Xu, Julien Cohen-Adad, Christophe Lenglet, Igor Nestrašil

**Affiliations:** Division of Clinical Behavioral Neuroscience, Department of Pediatrics, Masonic Institute for the Developing Brain, University of Minnesota, Minneapolis, MN, United States; NeuroPoly Lab, Institute of Biomedical Engineering, Polytechnique Montreal, Montreal, QC, Canada; Department of Radiology, Swiss Paraplegic Centre, Nottwil, Switzerland; Centre de recherche du CHU Sainte-Justine, Université de Montréal, Montreal, QC, Canada; Centre for Advanced Imaging, Australian Institute for Bioengineering and Nanotechnology, The University of Queensland, St Lucia, Australia; Department of Radiology, Faculty of Medicine, University of British Columbia, Vancouver, BC, Canada; Athinoula A. Martinos Center for Biomedical Imaging, Department of Radiology, Massachusetts General Hospital, Charlestown, MA, United States; Harvard Medical School, Boston, MA, United States; Harvard-Massachusetts Institute of Technology Health Sciences & Technology, Cambridge, MA, United States; School of Electrical Engineering and Computer Science, The University of Queensland, St Lucia, Australia; NMR Research Unit, Queen Square Multiple Sclerosis Centre, Department of Neuroinflammation, Queen Square Institute of Neurology, Faculty of Brain Sciences, University College London, London, United Kingdom; Department for Systems Neuroscience, University Medical Center Hamburg-Eppendorf, Hamburg, Germany; Department of Neurosurgery, Medical College of Wisconsin, Milwaukee, WI, United States; Clement J. Zablocki Veteran’s Affairs Medical Center, Milwaukee, WI, United States; Aix-Marseille Univ, CNRS, CRMBM, Marseille, France; APHM, Hopital Universitaire Timone, CEMEREM, Marseille, France; Department of Computer Engineering and Software Engineering, Polytechnique Montreal, Montreal, QC, Canada; Sherbrooke Connectivity Imaging Lab (SCIL), Computer Science department, Université de Sherbrooke, Sherbrooke, QC, Canada; Université de Strasbourg, CNRS, ICube, Strasbourg, France; Department of Radiology and Nuclear Medicine, University Hospital Brno and Masaryk University, Brno, Czech Republic; Department of Biophysics, Faculty of Medicine, Masaryk University, Brno, Czech Republic; McConnell Brain Imaging Centre, Montreal Neurological Institute, McGill University, Montreal, QC, Canada; Department of Physics and Astronomy, University of British Columbia, Vancouver, BC, Canada; Max Planck Research Group Pain Perception, Max Planck Institute for Human Cognitive and Brain Sciences, Leipzig, Germany; Stanford University, Stanford, CA, United States; Spinal Cord Injury Center Balgrist, University Hospital Zurich, University of Zurich, Zurich, Switzerland; Wellcome Centre for Human Neuroimaging, Queen Square Institute of Neurology, University College London, London, United Kingdom; Department of Neurophysics, Max Planck Institute for Human Cognitive and Brain Sciences, Leipzig, Germany; Institute of Nanotechnology, CNR, Rome, Italy; IRCCS Santa Lucia Foundation, Neuroimaging Laboratory, Rome, Italy; Department of Radiology, Juntendo University School of Medicine, Bunkyo, Tokyo, Japan; Department of Brain and Behavioural Sciences, University of Pavia, Pavia, Italy; Advanced Imaging and Artificial Intelligence Center, Neuroradiology Department, IRCCS Mondino Foundation, Pavia, Italy; MR Clinical Science, Philips Healthcare Canada, Mississauga, Canada; CREF - Museo storico della fisica e Centro studi e ricerche Enrico Fermi, Rome, Italy; Vall d’Hebron Institute of Oncology (VHIO), Vall d’Hebron Barcelona Hospital Campus, Barcelona, Spain; Center for Magnetic Resonance Research, Department of Radiology, University of Minnesota, Minneapolis, MN, United States; Faculty of Medicine, Masaryk University, Brno, Czech Republic; Department of Neurology, University Hospital Brno, Brno, Czech Republic; Multimodal and Functional Imaging Laboratory, Central European Institute of Technology, Brno, Czech Republic; Department of Radiology, Toho University Omori Medical Center, Tokyo, Japan; Department of Neurology, UCSF Weill Institute for Neurosciences, University of California San Francisco, San Francisco, CA, United States; Centre of Precision Rehabilitation for Spinal Pain (CPR Spine), University of Birmingham, Birmingham, United Kingdom; Centre for Human Brain Health, University of Birmingham, Birmingham, United Kingdom; Institute for Mental Health, University of Birmingham, Birmingham, United Kingdom; Biomedical Engineering and Imaging Institute, Department of Radiology, Graduate School of Biomedical Sciences, Icahn School of Medicine at Mount Sinai, New York, NY, United States; Department of Radiology, Baylor College of Medicine, Houston, TX, United States; Department of Psychiatry, Baylor College of Medicine, Houston, TX, United States; Neuro-X Institute, Ecole polytechnique fédérale de Lausanne, Geneva, Switzerland; Department of Radiology and Medical Informatics, Faculty of Medicine, University of Geneva, Geneva, Switzerland; Institute of Diagnostic and Interventional Neuroradiology, Faculty of Medicine and Carl Gustav Carus University Hospital, Technische Universität Dresden, Dresden, Germany; Division of Neurology, Faculty of Medicine, University of British Columbia, Vancouver, BC, Canada; CAS Key Laboratory of Behavioral Science, Institute of Psychology, Chinese Academy of Science, Beijing, China; Department of Psychology, University of Chinese Academy of Sciences, Beijing, China; First Department of Neurology, St. Anne’s University Hospital and Medical Faculty of Masaryk University, Brno, Czech Republic; IBM Poland, Department of Content Design, Cracow, Poland; Canon Medical Systems srl, Rome, Italy; Department of Pathology & Laboratory Medicine, University of British Columbia, Vancouver, BC, Canada; International Collaboration on Repair Discoveries (ICORD), University of British Columbia, Vancouver, BC, Canada; Department of Radiology, Beijing Tiantan Hospital, Capital Medical University, Beijing, China; Neuroimmunology and Multiple Sclerosis Unit, Laboratory of Advanced Imaging in Neuroimmunological Diseases (ImaginEM), Hospital Clínic Barcelona, Fundació de Recerca Clínic Barcelona-IDIBAPS and Universitat de Barcelona, Barcelona, Spain; Division of Pain Medicine, Department of Anesthesiology, Perioperative and Pain Medicine, Stanford University School of Medicine, Palo Alto, CA, United States; Department of Neurological Surgery, University of California, Davis, CA, United States; Section of Neuroradiology, Department of Radiology, Hospital Universitari Vall d’Hebron, Barcelona, Spain; Fondation Campus Biotech Geneva, Genève, Switzerland; Vanderbilt University Institute of Imaging Science, Vanderbilt University Medical Center, Nashville, TN, United States; Department of Radiology and Radiological Sciences, Vanderbilt University Medical Center, Nashville, TN, United States; Wellcome Centre For Integrative Neuroimaging, FMRIB, Nufﬁeld Department of Clinical Neurosciences, University of Oxford, Oxford, United Kingdom; Department of Radiology, Northwestern University, Chicago, IL, United States; e-Health Center, Universitat Oberta de Catalunya, Barcelona, Spain; Centre for Medical Image Computing, University College London, London, United Kingdom; School of Biomedical Sciences, Faculty of Health, Medicine and Behavioural Sciences, The University of Queensland, Brisbane, Australia; Department of Biomedical Sciences, Humanitas University, Pieve Emanuele (MI), Italy; Neuroradiology Unit, IRCCS Humanitas Research Hospital, Rozzano (MI), Italy; Department of Biomedical Engineering, Vanderbilt University, Nashville, TN, United States; Department of Neurosurgery, University of Oklahoma, Oklahoma City, OK, United States; The University of Tokyo Hospital, Radiology Center, Tokyo, Japan; Cardiff University Brain Research Imaging Centre (CUBRIC), School of Psychology, Cardiff University, Cardiff, Wales, United Kingdom; Mila - Quebec AI Institute, Montreal, QC, Canada; Department of Neurosurgery, Faculty of Medicine and Dentistry, Palacký University Olomouc, Olomouc, Czech Republic; Department of Neurology, Faculty of Medicine and Dentistry, Palacký University Olomouc, Olomouc, Czech Republic; Felix Bloch Institute for Solid State Physics, Faculty of Physics and Earth System Sciences, Leipzig University, Leipzig, Germany; Department of Neurosciences, Imaging, and Clinical Sciences, ‘G. D’Annunzio’ University of Chieti-Pescara, Chieti, Italy; Institute for Advanced Biomedical Technologies, ‘G. D’Annunzio’ University of Chieti-Pescara, Chieti, Italy; Functional Neuroimaging Unit, CRIUGM, University of Montreal, Montreal, Canada

**Keywords:** spinal cord, brain, body height and weight, intracranial volume, structural magnetic resonance imaging, in vivo human neuroimaging

## Abstract

Clinical research emphasizes the implementation of rigorous and reproducible study designs that rely on between-group matching or controlling for sources of biological variation such as subject’s sex and age. However, corrections for body size (i.e., height and weight) are mostly lacking in clinical neuroimaging designs. This study investigates the importance of body size parameters in their relationship with spinal cord (SC) and brain magnetic resonance imaging (MRI) metrics. Data were derived from a cosmopolitan population of 267 healthy human adults (age 30.1 ± 6.6 years old, 125 females). We show that body height correlates with brain gray matter (GM) volume, cortical GM volume, total cerebellar volume, brainstem volume, and cross-sectional area (CSA) of cervical SC white matter (CSA-WM; 0.44 ≤ r ≤ 0.62). Intracranial volume (ICV) correlates with body height (r = 0.46) and the brain volumes and CSA-WM (0.37 ≤ r ≤ 0.77). In comparison, age correlates with cortical GM volume, precentral GM volume, and cortical thickness (-0.21 ≥ r ≥ -0.27). Body weight correlates with magnetization transfer ratio in the SC WM, dorsal columns, and lateral corticospinal tracts (-0.20 ≥ r ≥ -0.23). Body weight further correlates with the mean diffusivity derived from diffusion tensor imaging (DTI) in SC WM (r = -0.20) and dorsal columns (-0.21), but only in males. CSA-WM correlates with brain volumes (0.39 ≤ r ≤ 0.64), and with precentral gyrus thickness and DTI-based fractional anisotropy in SC dorsal columns and SC lateral corticospinal tracts (-0.22 ≥ r ≥ -0.25). Linear mixture of age, sex, or sex and age, explained 2 ± 2%, 24 ± 10%, or 26 ± 10%, of data variance in brain volumetry and SC CSA. The amount of explained variance increased to 33 ± 11%, 41 ± 17%, or 46 ± 17%, when body height, ICV, or body height and ICV were added into the mixture model. In females, the explained variances halved suggesting another unidentified biological factor(s) determining females’ central nervous system (CNS) morphology. In conclusion, body size and ICV are significant biological variables. Along with sex and age, body size should therefore be included as a mandatory variable in the design of clinical neuroimaging studies examining SC and brain structure; and body size and ICV should be considered as covariates in statistical analyses. Normalization of different brain regions with ICV diminishes their correlations with body size, but simultaneously amplifies ICV-related variance (r = 0.72 ± 0.07) and suppresses volume variance of the different brain regions (r = 0.12 ± 0.19) in the normalized measurements.

## 1 Introduction

Knowledge about the relationship between body size (i.e., height and weight), spinal cord (SC), and brain structure is essential for a mechanistic understanding of human physiology and pathophysiology and, consequently, developing biomarkers critical for robust clinical trial designs. Besides sex and age, numerous other factors influence body size, including genetic makeup, race and ethnicity, socioeconomic and environmental factors, as well as developmental determinants. There are also diseases affecting physical makeup, spanning chronic conditions (i.e., anemia, asthma, celiac disease, inflammatory bowel disease, kidney, or heart insufficiency), hormonal diseases (i.e., growth or thyroid hormone disbalances), and/or rare disorders such as achondroplasia and Down, Noonan, or Turner syndromes (Butler et al., 2022;Pierpont et al., 2024). For example, patients diagnosed with Friedreich ataxia tend to be underweight in young age and overweight in adulthood (Boesch & Indelicato, 2021;M. Patel et al., 2021). Patients with different types of mucopolysaccharidoses are known to present with a short stature (Lin et al., 2019;Muschol et al., 2019;P. Patel et al., 2014). While neuroimaging measurements are usually compared with a healthy population, neither body height nor weight has been rigorously considered as putative confounding factors, normalization factors, and/or as variables necessary for an inter-population matching (Harding et al., 2021;Joers et al., 2022;Kovac et al., 2022;Provenzale et al., 2015;Rezende et al., 2023;Yund et al., 2015). Such a study design deficit can lead to bias in clinical outcomes, which applies even more explicitly to studies where the typical body size of the patients’ cohort differs from that of the control group. To assess the significance and importance of body size correction, we have investigated the impact of body size on structural neuroimaging measurements in the SC and brain of a healthy human population.*If the effect is significant, future clinical research studies and trials utilizing neuroimaging should include body size as a potential confounding biological factor to avoid bias in clinical outcomes*.

Evolutionary biology has identified links between species’ body weight, SC, and cerebral weights (MacLarnon, 1996b), and between spinal canal dimensions and adjacent cord (MacLarnon, 1995,1996a). Cadaveric human measurements revealed links between the cross-sectional area (CSA) of the cervical SC and cerebral weight, body height, and age (Kameyama et al., 1994). However, in vivo evidence of such a relationship between body size and central nervous system (CNS) structure is limited to a few magnetic resonance imaging (MRI) studies. In vivo CSA of the upper cervical SC (i.e., C2/3 segment) appears to be determined by both the cerebral volume and white matter (WM) content of cerebrospinal tracts (Engl et al., 2013). Recent exploration of the UK Biobank imaging dataset observed weak in vivo links between the CSA of the C2/3 SC segment and body height and weight, and moderate links between the CSA and brain and thalamus volumes (Alfaro-Almagro et al., 2018;Bédard & Cohen-Adad, 2022;Littlejohns et al., 2020). Weak correlations between body height, CSA of the SC (CSA-SC), and gray matter (GM), as well as brain volume scaling were also reported on a concurrent in vivo dataset (Papinutto et al., 2020). However, these effects disappeared when sex was controlled for (Papinutto et al., 2020). Additionally, the in vivo CSA of peripheral nerves has also been shown to correlate moderately with body height, body weight, and body mass index (BMI), but not age (Kronlage et al., 2019). Whether SC WM and GM contents are equally correlated with body size and distinct brain morphology has not been satisfactorily determined. Our**first hypothesis**was, therefore, that*“CSA of cervical SC WM and GM interacts with body size and morphology of distinct brain structures”*; we tested this premise by utilizing a multi-center*spine-generic*MRI dataset. The dataset allows for the separate assessment of cervical SC WM and GM morphology in a large cohort of healthy cosmopolitan volunteers with available demographic records and images of cerebral morphology (Cohen-Adad et al., 2021a,2021b).

Myelin content is an essential characteristic of the neural tissue microstructural integrity (Levitan & Kaczmarek, 2002). In the CNS, the ratio between axon diameter and diameter of the total nerve fiber (axon and myelin) is 0.6–0.7 (Susuki, 2010). As SC axons generally have larger diameters than axons within the brain (Aboitiz et al., 1992;Duval et al., 2019;Saliani et al., 2017;Veraart et al., 2020), SC myelin sheaths are often also thicker, increasing the overall diameter of the myelinated axons. Thicker myelin sheaths around axons accelerate nerve conduction speed independent of the axonal diameter (Rushton, 1951;Zalc, 2006,^2016^). Assuming a fairly constant axon/fiber diameter ratio (Susuki, 2010), thicker myelin sheaths are, therefore, expected for species with larger body sizes (Zalc, 2006,^2016^). Considering intra-species variability in body size, the overall degree of SC myelination might be influenced by the body size of a given specimen. If true, the influence of body size on myelin content may be detectable in SC images sensitive to tissue microstructure, such as diffusion tensor imaging (DTI) or magnetization transfer ratio (MTR) imaging. Both DTI and MTR image contrasts are available within the*spine-generic*dataset (Cohen-Adad et al., 2021a,2021b). Moreover, body weight and BMI are correlated with MTR of peripheral nerves and muscles (Fösleitner et al., 2022). Our**second hypothesis**was, therefore, that*“SC microstructure, as measured using MTR and DTI, interacts with body size”*.

Finally, the human brain volume and CSA-SC differ between sexes (Bédard & Cohen-Adad, 2022;Giedd et al., 1996;Papinutto et al., 2015,^2020^). It is well established that brain volume shrinks and cortical GM thickness thins with aging (Fjell et al., 2013;Peters, 2006;Thambisetty et al., 2010), with both processes accelerating after 45 years of age (Heymsfield et al., 2009;Peters, 2006). However, results obtained from pathological (Callaghan et al., 2014;Kameyama et al., 1994;Weitzenkamp et al., 2001;Zhang et al., 1996;Zhou et al., 1996) and neuroimaging (Callaghan et al., 2014) studies investigating the relationships between age and SC CSA have been less consistent. Recent high-resolution in vivo neuroimaging indeed observed weaker and slower aging effects in SC CSA than those described for brain morphology (Bédard & Cohen-Adad, 2022;Papinutto et al., 2015,^2020^). The UK Biobank dataset already showed that physical measures, including body height and weight, strongly impact quantitative brain structural measures in a population of 40–69-year olds while adjusted for sex and age (Miller et al., 2016). Outside of the UK Biobank, links between body size and brain volume have been reported with inconsistent results, spanning significant relationships with a stronger height influence (Baaré et al., 2001;Bédard & Cohen-Adad, 2022;Heymsfield et al., 2009) or non-significant findings (Willerman et al., 1991). Therefore, our**third hypothesis**was that:*“Cerebral morphology interacts with body height more profoundly than with body weight and age.”*

In addition to body size, brain morphology is often normalized with subject-specific intracranial volume (ICV) (Voevodskaya et al., 2014;Whitwell et al., 2001;Xie et al., 2005). ICV and head size were identified as significant covariates determining brain structure more profoundly than the body size in the UK Biobank dataset (Miller et al., 2016). That led us to the**fourth hypothesis**:*“Body size increases the predictive power of CNS structure.”*We tested our hypotheses by utilizing the*spine-generic*dataset of predominantly non-elderly healthy adults and considering sex effects.

## 2 Methods

### 2.1 Structural MRI data

Signed informed consent was obtained from all participants under the compliance of the corresponding local ethics committee (more info in the*Scientific Data*paper;Cohen-Adad et al., 2021b). The*spine-generic*protocol 3T MRI data were acquired once for each participant. Siemens scanners were used in 180 (67.41%) acquisitions, Philips scanners in 50 (18.72%) acquisitions, and GE scanners in 37 (13.87%) acquisitions. 3D T1w scans were utilized to estimate cerebral volumes and cortical thicknesses. 3D T2w scans were utilized to assess the cross-sectional area (CSA) of the cervical spinal cord (SC). Axial T2*w scans were utilized to estimate the CSA of white (WM) and gray (GM) matter of the cervical SC. Diffusion weighted imaging was utilized to estimate diffusion tensor imaging (DTI) and the corresponding microstructural maps for the cervical SC. GRE-T1w, GRE-MT1, and GRE-MT0 scans were used to derive the magnetization transfer ratio (MTR) maps in the cervical SC. More detailed information about protocol settings and scanner subtypes can be found in the*spine-generic*protocol original papers (Cohen-Adad et al., 2021a,2021b).

### 2.2 Image analysis

The same image processing pipeline was employed here, utilizing the Spinal Cord Toolbox (SCT) version 6.1 (De Leener et al., 2017), as developed originally for the*spine-generic*protocol (Cohen-Adad et al., 2021a,2021b). The*spine-generic*database (Cohen-Adad et al., 2021b) includes manual SC and/or WM/GM segmentation and cervical level labeling for MRI scans where the automated segmentation and/or labeling methods were inaccurate. In cases where manual segmentation existed, we used the existing manual segmentation to secure result reproducibility and reliability. CSA of the whole SC (CSA-SC) was computed and averaged from cervical C3-4 vertebral levels of the 3D T2w scan. CSA of WM and GM structures (CSA-WM, CSA-GM) was computed and averaged from cervical C3-4 levels of the axial T2*w scan. C3-4 levels were selected for CSA measurements since the T2*w imaging protocol had set the center of the field of view at the C3/4 disk and because C3-4 levels still contain the most sensory and motor fiber bundles. C3-4 average represents a robust representative morphological measurement as the CSA demonstrates high intra-individual correlation over segments (Healy et al., 2012;Kameyama et al., 1996), although the absolute CSA values inter-individually vary (Cohen-Adad et al., 2021b). All CSA measurements were measured in mm^2^units. Fractional anisotropy (FA), mean diffusivity (MD), radial diffusivity (RD), and MTR were estimated from cervical C2-5 vertebral levels for WM, GM, bilateral lateral corticospinal tracts, and bilateral dorsal columns utilizing the PAM50 atlas co-registration and weighted average techniques (Lévy et al., 2015). The C2-5 segment range was selected for DTI and MTR averaging to guarantee the robustness of the tract-specific measurements with minimal partial volume effects (Lévy et al., 2015).

Brain volume was segmented and parceled at partial sub-structures from 3D T1w scans with FreeSurfer ver. 7.2 (Fischl, 2012). All FreeSurfer-based brain imaging results were visually reviewed for accuracy and any inaccurate segmentations were fixed. During initial post-processing, 112 scans (46.86%) had inaccurate segmentation. Corrections were performed using FreeSurfer edits (i.e., control points, pial edits, both control points and pial edits, and recon-all interventions), AFNI’s (Analysis of Functional NeuroImages) 3dUnifize tool (Cox, 1996), and/or lesion fill using ITK-SNAP (Yushkevich et al., 2006) and FSL (Battaglini et al., 2012;Jenkinson et al., 2012). The lesion fill was utilized for one scan*(sub-mountSinai01*) where minor white matter hypo-intensities were present. Volumes of brain (BrainVol), brain GM (BrainGMVol), cortical GM (CorticalGMVol), cortical WM (CorticalWMVol), subcortical GM (SubCortGMVol, including amygdala, caudate, hippocampus, nucleus accumbens, pallidum, putamen, thalamus, ventral diencephalon, and substancia nigra), thalamus (ThalamusVol), cerebellum (CerebellumVol), brainstem (BrainStemVol), precentral cortex GM (PrecentralGMVol), postcentral cortex GM (PostcentralGMVol), and intracranial volume (ICV) were measured from the FreeSurfer segmentations in mm^3^units. A sub-analysis also utilized brain volume measurements as relative ratios of the whole ICV. Cortical thickness (cortical thickness), thickness of the precentral (PrecentralG Thickness), and postcentral gyrus (PostcentralG Thickness) were averaged across the left and right hemispheres as derived from the surface-based cortical parcellation. Precentral and postcentral cortices, motor and somatosensory cerebral centers, were investigated because the majority of the cervical SC WM cross-section are the motor and somatosensory pathways.

### 2.3 Exclusion of spinal cord and brain structural measurements

Spinal cord images were analyzed for all 267 participants. Cross-sectional area of SC (CSA-SC) was not estimated for four participants (listed in the category “csa_t2” in exclude.yml file, which contains the excluded subject ID and the verbal explanation of the exclusion; 1.50% of the dataset), CSA of WM and GM (CSA-WM and CSA-GM, respectively) was not estimated for four different participants (category “csa_gm” in the exclude.yml file; 1.50%), DTI measurements were not estimated for four participants (categories “dti_fa,” “dti_md,” and “dti_rd” in the exclude.yml file; 1.50%), and MTR measurements were not estimated for five participants (category “mtr” in the exclude.yml file; 1.87%). The exclude.yml file is available at:https://github.com/spine-generic/data-multi-subject/blob/r20231212/exclude.yml. The most common reasons for SC measurement exclusions were (*i*) motion artifacts, (*ii*) subject repositioning during data acquisition, (*iii*) poor data quality, (*iv*) wrong field of view placement, or (*v*) not following required imaging parameters. The analysis excluded all CSA, DTI, and MTR SC measurements for one additional subject (*sub-mniS05*; 0.37% of the dataset) due to severe degenerative cervical SC compression (maximal compression at C3/C4 level).

We analyzed brain images from 239 participants (89.51% of the dataset). We excluded T1w scans of 28 participants (10.49%) from the analysis because the images demonstrated field of view cutoffs (18 scans; 6.74%), defacing errors (5 scans; 1.87%), poor image contrast in superior cerebral regions (4 scans; 1.50%), and severe motion artifacts (1 scan; 0.37%). Excluded brain scans are listed in the exclude.yml file as the category “brain_t1.”

### 2.4 Body mass measurements

Body mass index (Nuttall, 2015), body surface area (Du Bois & Du Bois, 1989), and lean body weight (James, 1976) were estimated utilizing body height and weight measurements.

### 2.5 Effect of ICV normalization on cross-sectional brain volume measurements

Let*x*be a region-specific brain volume measurement. If*x*≪ ICV (e.g., ThalamusVol), then a normalized brain volume*x_norm_= x/ICV*becomes proportional to ICV^-1^and an effect of the underlying region-specific brain structure can be minimized in the normalized volume measurement. In the statistical analysis, we correlated*x_norm_*with original*x*[mm^3^] and ICV^-1^to test whether*x_norm_*preserves more information about*x*or ICV. We also correlated*x*and*x_norm_*with body size and assessed outcome differences.

### 2.6 Statistical analysis

Statistical analysis and figure visualization were implemented in the programming environment MATLAB R2021b (*Natick, USA*). Each variable or log(variable) was normalized into the space of the normal distribution and the Kruskall–Wallis test tested whether investigated variables meet conditions for Gaussian or log-Gaussian distribution (p < 0.05). Between-group differences were tested with two-sample or paired t-tests. Correlation analysis utilized Pearson (*r*) and Spearman (*ρ*) correlation coefficients, considering correlation to be significant if p_FWE_< 0.05 (FWE—family-wise error correction) after the Bonferroni multiple-comparison correction. Correlation coefficients were estimated for raw and normalized dataset values, where the manufacturer-specific average was subtracted from all SC quantitative measurements to minimize the effects of the previously reported inter-manufacturer variability in the spine-generic dataset (Cohen-Adad et al., 2021b). For SC DTI and MTR correlation analysis, GE scanner raw values were excluded (i.e., 13.87% of the dataset) due to strong offsets compared with Siemens and Philips scanner values. SC qMRI measurements, FreeSurfer-based brain measurements, age, body height, and body weight were cross-correlated, and significant correlations (after the Bonferroni correction) were identified. The dataset was split into males and females and the correlation analysis was post-hoc repeated to address sex effects in the data demonstrating significant correlations. Due to the reduced sample size at half, the uncorrected p < 0.05 was considered significant in the post-hoc analysis investigating the sex effects. The correlation analysis was also post-hoc repeated for SC measurements while excluding all 64 subjects with degenerative cervical SC compression (as identified in the*spine-generic*database; r20231212) to test for the compression effects on the study outcomes. The critical p_FWE_< 0.05 remained here, although the dataset was reduced to 76% of its original size.

Manufacturer-specific average was subtracted from all SC structural measurements. Then, all multivariate data were normalized to mean = 0 and standard deviation STD = 1 for each examined variable. Such normalized data formed an input matrix for exploratory principal component analysis (PCA) optimized via singular value decomposition. Variables were visualized in the space of orthogonal principal components via biplot projections, and between-variable relationships were quantified and interpreted in the rotated principal space explaining the majority of the data variance.

Several linear regression models (Eqs. 1–14) were estimated for the SC and brain structural measurements (***y***) demonstrating significant correlation with age, ICV, body height, and/or body weight, respectively. Models’ coefficients of determination (*R^2^*) objectively assessed which demographic variable or set of demographic variables explained most of the demography-related variance in the SC and brain structure. Models utilizing simultaneous regression of body height and body weight were not utilized as body height and weight are strongly linearly dependent variables. The variable***y**_0_*represents the model’s constant member, the β parameters are model regression coefficients (Eqs. 1–14). Categorical variable***Sex***was modeled as a vector of values 0.5 at positions of males and of values -0.5 at positions of females. Manufacturer-specific average was subtracted from all SC structural measurements before the regression analysis.

$$\text{y}\propto {\text{y}}_{0}+\beta_{Age}\;\cdot \;\text{Age}$$ (1)

$$\text{y}\propto {\text{y}}_{0}+\beta_{Sex}\;\cdot \;\text{Sex}$$ (2)

$$\text{y}\propto {\text{y}}_{0}+\beta_{Height}\;\cdot \;\text{Height}$$ (3)

$$\text{y}\propto {\text{y}}_{0}+\beta_{Weight}\;\cdot \;\text{Weight}$$ (4)

$$\text{y}\propto {\text{y}}_{0}+\beta_{ICV}\;\cdot \;\text{ICV}$$ (5)

$$\text{y}\propto {\text{y}}_{0}+\beta_{Sex}\;\cdot \;\text{Sex}+\;\beta_{Age}\;\cdot \;\text{Age}$$ (6)

$$\text{y}\propto {\text{y}}_{0}+\beta_{Sex}\;\cdot \;\text{Sex}+\;\beta_{Height}\;\cdot \;\text{Height}$$ (7)

$$\text{y}\propto {\text{y}}_{0}+\beta_{Sex}\;\cdot \;\text{Sex}+\beta_{Weight}\;\cdot \;\text{Weight}$$ (8)

$$\text{y}\propto {\text{y}}_{0}+\beta_{Sex}\cdot \;\text{Sex}+\beta_{ICV}\;\cdot \;\text{ICV}$$ (9)

$$\text{y}\propto {\text{y}}_{0}+\beta_{Sex}\;\cdot \;\text{Sex}+\beta_{Age}\;\cdot \;\text{Age}+\beta_{Height}\;\cdot \;\text{Height}$$ (10)

$$\text{y}\propto {\text{y}}_{0}+\beta_{Sex}\;\cdot \;\text{Sex}+\beta_{Age}\;\cdot \;\;\text{Age}+\beta_{Weight}\;\cdot \;\text{Weight}$$ (11)

$$\text{y}\propto {\text{y}}_{0}+\beta_{Sex}\cdot \;\text{Sex}+\beta_{Age}\cdot \;\text{Age}+\beta_{ICV}\cdot \;\text{ICV}$$ (12)

$$\text{y}\propto {\text{y}}_{0}+\beta_{Sex}\cdot \;\text{Sex}+\beta_{Age}\cdot \;\text{Age}+\beta_{ICV}\cdot \;\text{ICV}+\beta_{Height}\cdot \;\text{Height}$$ (13)

$$\text{y}\propto {\text{y}}_{0}+\beta_{Sex}\cdot \;\text{Sex}+\beta_{Age}\cdot \;\text{Age}+\beta_{ICV}\cdot \;\text{ICV}+\beta_{Weight}\cdot \;\text{Weight}$$ (14)

Stepwise linear regression was also performed utilizing the same variables as in theEqs. 1–14. The threshold p-value to consider a variable as statistically significant for the multiple linear regression model was p < 0.05.

## 3 Results

### 3.1 Study cohort demography

Structural MRI data were acquired in a cohort of 267 neurologically healthy (self-reported) volunteers whose demographic data and intracranial volumes are summarized inTable 1. There was no significant difference in age between females and males, but body height, weight, BMI, body surface area (BSA), lean body weight (LBW), and ICV differed (Table 1). Female dataset provided slightly lower variance in age, body height, body weight, BSA, LBW, and ICV, and slightly higher variance in BMI (Table 1). All subject-specific demographic data are available at:https://github.com/spine-generic/data-multi-subject/blob/r20231212/participants.tsv. Body height and weight were significantly intercorrelated (Pearson correlation coefficient r = 0.702). ICV correlated significantly with body height (r = 0.463) and weight (0.357). However, the correlation coefficient magnitudes shows that ICV and body size were also carrying portions of mutually independent information. Moreover, only correlation between ICV and body height in males survived significance when the dataset was split regarding the sex (Table 2).

**Table 1.** Demography and intracranial volume of recruited cohort.

	All	Female	Male	p-value
Number of subjects	267	125 (46.82%)	142 (53.18%)	
Age [years]	30.1 ± 6.6 (19.0–56.0)	29.4 ± 6.4 (20.0–56.0)	30.6 ± 6.7 (19.0–56.0)	0.1537
Height [cm]	172.1 ± 10.0 (148.0–203.0)	164.9 ± 6.5 (148.0–185.0)	178.5 ± 8.0 (161.0–203.0)	<0.0001
Weight [kg]	68.3 ± 13.4 (41.0–120.0)	59.5 ± 9.7 (41.0–86.0)	76.0 ± 11.4 (55.0–120.0)	<0.0001
BMI [kg/m ^2^ ]	22.9 ± 3.3 (16.6–35.5)	21.9 ± 3.5 (16.6–35.5)	23.8 ± 2.8 (18.6–35.1)	<0.0001
BSA [m ^2^ ]	1.80 ± 0.21 (1.35–2.42)	1.65 ± 0.13 (1.35–2.01)	1.94 ± 0.17 (1.60–2.42)	<0.0001
LBW [kg]	52.5 ± 10.0 (33.5–78.1)	44.0 ± 4.5 (33.5–56.0)	60.2 ± 6.8 (46.2–78.1)	<0.0001
ICV [*10 ^6^ mm ^3^ ]	1.48 ± 0.23 (0.95–2.10)	1.34 ± 0.18 (0.95–1.72)	1.59 ± 0.20 (0.99–2.10)	<0.0001

Cell values are as follows: mean ± standard deviation (minimum–maximum). P-value was derived from a two-sample t-test comparing variable distributions between females and males.

BMI, body mass index; BSA, body surface area; LBW, lean body weight; ICV, intracranial volume.

**Table 2.** Pearson correlation coefficients between body size, age, spinal cord structure, brain structure, and intracranial volume, and post hoc sex effects in the correlation analysis.

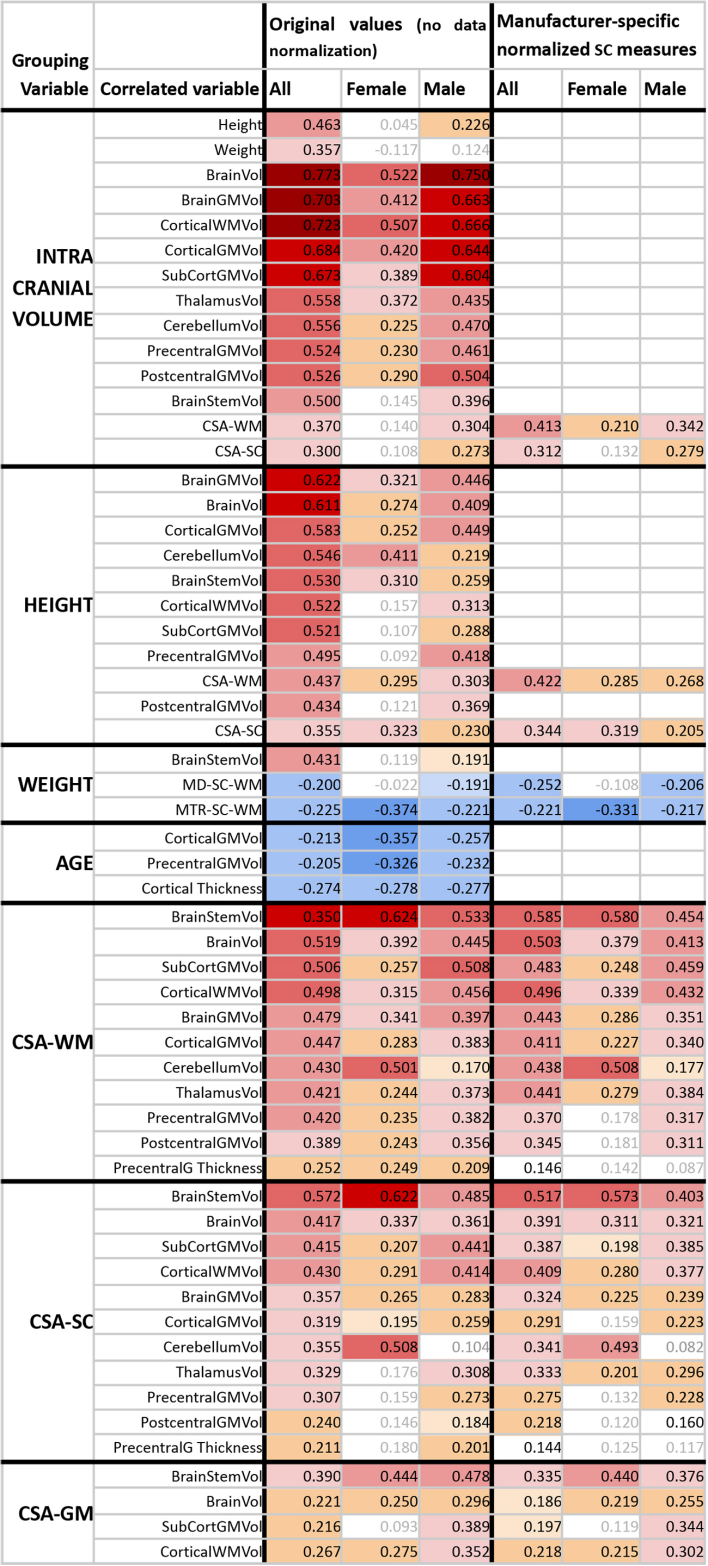

CSA, cross-sectional area; SC, spinal cord; WM, white matter; GM, gray matter; Vol, volume. The correlation analysis on non-normalized data identified a list of variable pairs with a correlation coefficient of p_FWE_< 0.05. The final list here only selects the variable pairs with a significant post hoc Pearson correlation coefficient (uncorrected p < 0.05 in at least one sex-specific sub-dataset (i.e., female and/or male). Insignificant correlation coefficients, which did not meet the post hoc condition uncorrected p < 0.05, are written with gray font. Positive correlation coefficients (p < 0.05) are visualized as a yellow–orange–pink–red shade of the table background. Negative correlation coefficients (p < 0.05) are visualized as a light blue–blue shade of the table background. CSA was measured as averages between C3-C4 segments. DTI and MTR were calculated as averages between C2-C5 segments. The column denoted*“Original values”*reports correlation coefficients for raw measurements with no normalization procedure prior to the correlation analysis. The column denoted*“Manufacturer-specific normalized SC measures”*reports correlation coefficients for SC structural measurements, which were normalized to zero mean for each scanner manufacturer before correlation analysis. Empty cells in the right half of the table represent combinations where no updated correlation coefficients were measured, because the utilized normalization of SC structural measurements had no effect on these correlation coefficients. Brain structural measurements were not considered necessary to normalize as we did not observe strong scanner-related effects in brain macrostructural measurements.

The 112 subjects (42 / 37.5% females) with manual edits necessary in brain image analysis were about 2 years younger (p = 0.0063; non-edited 31 ± 6 years; edited 29 ± 7 years), 5 cm taller (p = 0.0007; non-edited 170 ± 10 cm; edited 175 ± 10 cm), with 0.06 m^2^higher BSA (p = 0.0276) and 3 kg higher LBW (p = 0.0237). Body weight (p = 0.1848), BMI (p = 0.4393), and ICV (p = 0.0719; non-edited [1.45 ± 0.22]*10^6^mm^3^; edited [1.51 ± 0.24]*10^6^mm^3^) did not differ. Most variables that differed appear proportional to the higher frequency of manual edits in male brain scans. Seventy-seven of the 112 manually edited scans were acquired with the Siemens MRI scanner (49% of the Siemens scans), 18 of the 112 with the Philips scanner (37%), and 17 of the 112 with the GE scanner (52%).

### 3.2 Minimal impact of manual segmentation edits on accuracy of brain morphology

BrainGMVol was higher at about 27000 mm^3^(estimated error (Eq. 15) +3.8%; p = 0.0182) in males and 26400 mm^3^(error +2.8%; p = 0.0056) in females. CorticalGMVol was higher at about 25600 mm^3^(error +4.2%; p = 0.0061) in males and 23500 mm^3^(error +5.0%; p = 0.0028) in females. PrecentralGMVol was higher at about 1400 mm^3^(error +5.1%; p = 0.0203) in males. Cortical thickness was higher at about 0.04 mm (error +1.5%; p = 0.0349) in females. Note that the detected mm thickness error is markedly below the imaging spatial resolution. Otherwise, no differences were observed in brain morphology measurements regarding non-edited and manually edited results (Supplementary Table S1). Because all detected errors were ≤5%, we conclude a minimal impact of the utilized manual edits on brain morphology measurements. Contrary, the errors would be much larger without the editing.

error=200* (edited​_mean−non-edited​_mean)​/  (edited​_mean+non-edited​_mean). (15)

### 3.3 Gaussianity of demographic and structural MRI data

Age demonstrated log-Gaussian distribution. Body height demonstrated neither Gaussian (p = 0.0089) nor log-Gaussian (p = 0.0259) distributions. Body weight, BMI, all CSA measurements, all SC DTI measurements, and all brain morphological measurements demonstrated Gaussian distributions. All SC MTR measurements demonstrated neither Gaussian (p < 0.0086) nor log-Gaussian (p < 0.0009) distributions.

### 3.4 Body size interacts with the structure of spinal cord white matter

**Fig. 1.**
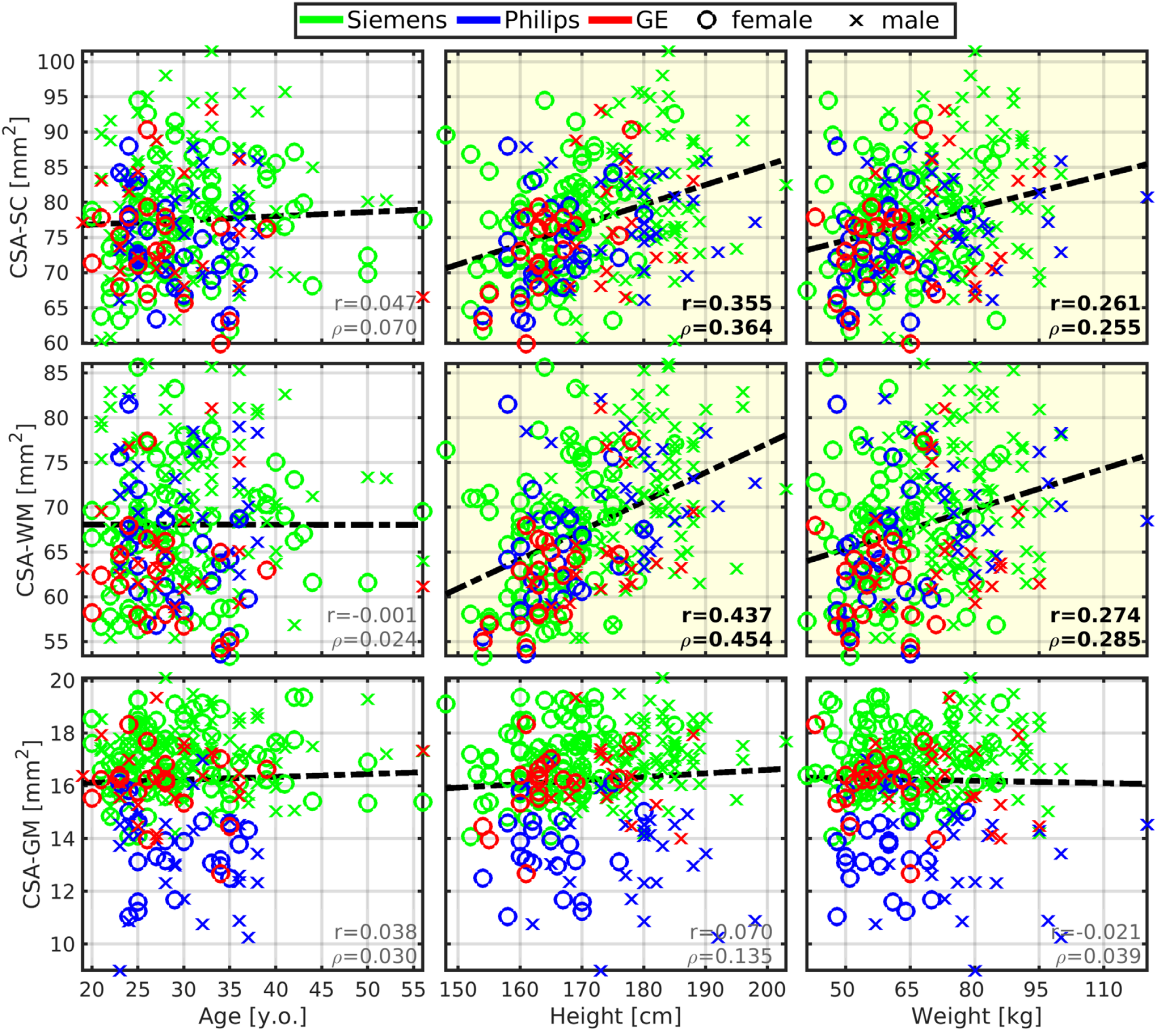
Cross-sectional area of spinal cord white matter correlates with body height and weight. CSA, cross-sectional area; SC, spinal cord; WM, white matter; GM, gray matter; r, Pearson correlation coefficient;*ρ*, Spearman correlation coefficient. All spinal cord measurements were averaged from cervical C3-4 levels. Regression lines (i.e., the dashed black lines) were estimated from all available data points. Plots with statistically significant correlation (p_FWE_< 0.05) are highlighted with yellow background, and corresponding r and*ρ*values are highlighted with black bold font.

**Fig. 2.**
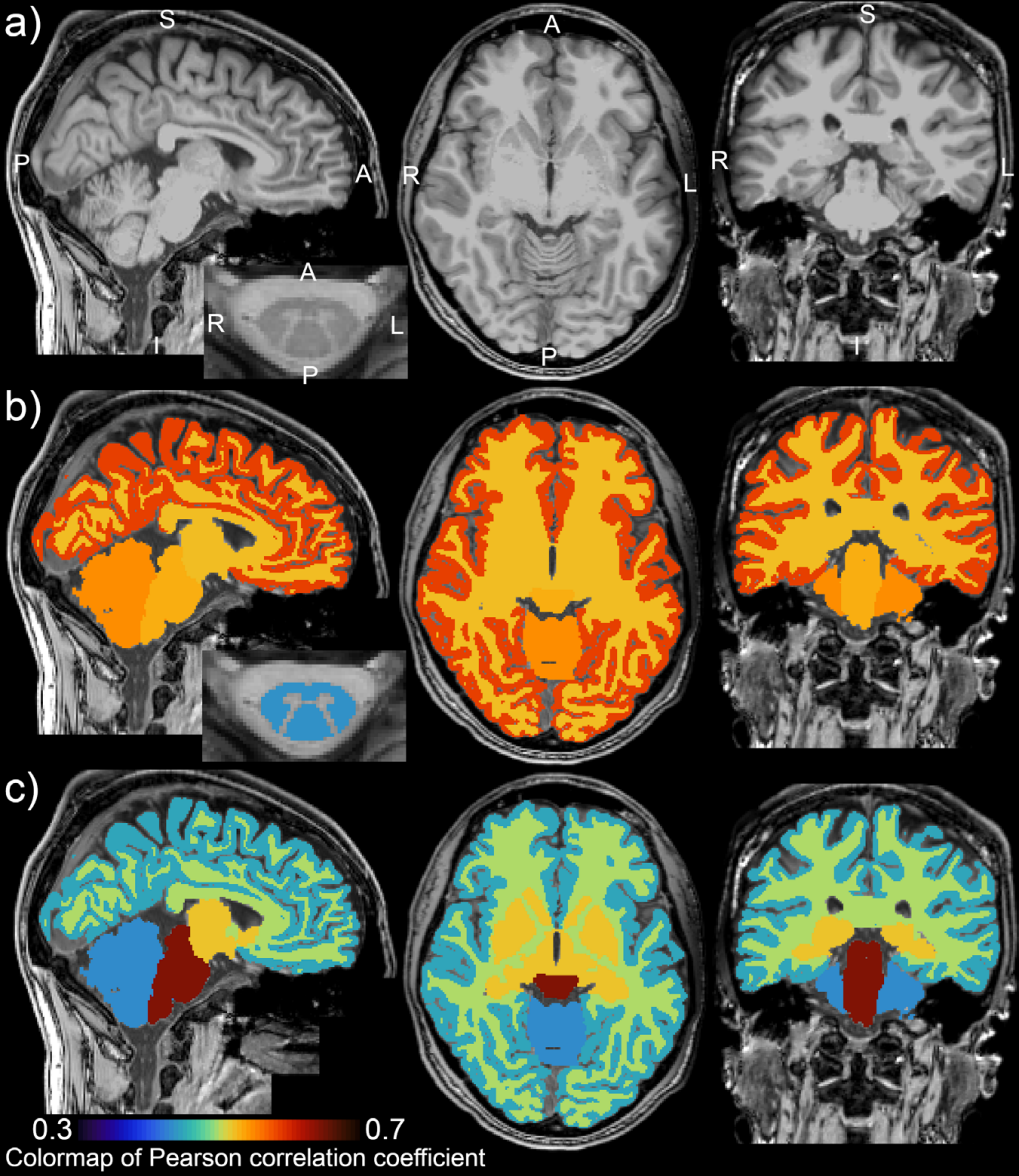
Pearson correlation coefficient maps showing interactions between body height and morphology of the central nervous system. (a) Representative image of brain and spinal cord (SC) anatomy. The brain scan shows cortical gray matter (GM), cerebral white matter (WM), subcortical GM structures, brainstem, and cerebellum. The axial SC scan shows the WM and GM anatomy at the C3/C4 level. Image orientation is described in (a): A, anterior; P, posterior; S, superior; I, Inferior; L, left; and R, right. (b) Pearson correlation coefficient between body height and (i) cortical GM volume, (ii) cerebral WM volume, (iii) subcortical GM structure volume, (iv) brainstem volume, (v) cerebellar volume, and (vi) cross-sectional area (CSA) of cervical SC WM at C3/C4 level. The colormap for the correlation values is shown in the left bottom corner of the figure. All correlations are significant (p_FWE_< 0.05). Regarding the investigated list of structures, body height demonstrated the strongest correlation with the cortical GM volume. (c) Pearson correlation coefficient between the CSA of cervical WM at C3/C4 level and (i) cortical GM volume, (ii) cerebral WM volume, (iii) subcortical GM structure volume, (iv) brainstem volume, and (v) cerebellar volume. The colormap for the correlation values is shown in the left bottom corner of the figure. All correlations are significant (p_FWE_< 0.05). The correlation map shows a descending gradient from the brainstem through subcortical GM structures and cerebral WM to cortical GM. The gradient may be driven by the increasing distance to the cervical SC level and decreasing relative volume of common tract pathways. The cerebellum shows the lowest, yet significant, correlation level. This finding may be explained by the fact that the cerebrum is more strongly and directly interconnected to the peripheral nervous system via SC than the cerebellum, with spinocerebellar tracts as the primary direct connections (Chandar & Freeman, 2014;Purves et al., 2001).

CSA of SC (CSA-SC) was correlated moderately with body height (r = 0.355;Fig. 1;Table 2), and this correlation strength was higher for the CSA-WM subregion (r = 0.437;Fig. 1;Table 2). CSA-SC and CSA-WM demonstrated minimal differences between scanner manufacturers (Fig. 1). Thus, the same correlation patterns for height were preserved even when manufacturer-specific averages of CSA-SC or CSA-WM were subtracted from corresponding CSA measurements prior to the correlation analysis in order to normalize data across scanners (Table 2). The correlation between body height and CSA-SC/CSA-WM remained significant even when the dataset was split into males and females (Table 2). Body weight was correlated weakly with CSA-SC (r = 0.261) and CSA-WM (r = 0.274). In addition, this correlation was not significant when the dataset was split into males and females (Supplementary Table S2). CSA-GM was not correlated with body size (Fig. 1;Supplementary Table S2). The CSA-GM measured on Philips scanners demonstrated a lower mean offset than for data obtained on Siemens and GE scanners (Fig. 1; p < 0.0001). Neither CSA measurement (i.e., SC, WM, GM) was correlated with age (Fig. 1;Supplementary Table S2). Overall, body height is the demographic variable driving the impact on CSA-WM and explaining the majority of demography-related variability in CSA measurements (Fig. 2b;Supplementary Table S3). ICV correlated with CSA-WM and CSA-SC less profoundly than body height (Fig. 1;Table 2;Supplementary Fig. S1).

**Fig. 3.**
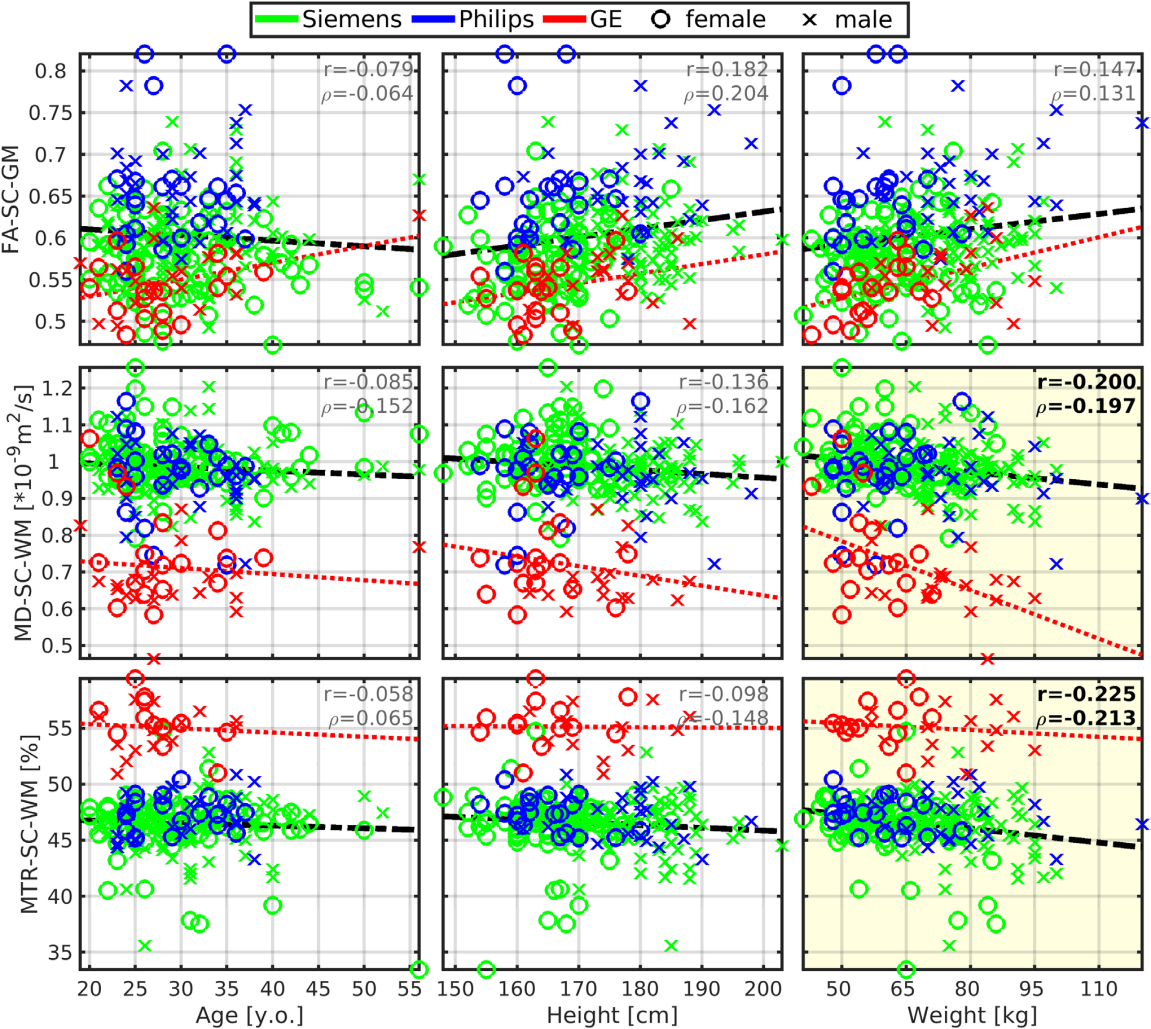
Mean diffusivity and magnetization transfer ratio in spinal cord white matter correlates with body weight. GM, gray matter; WM, white matter; SC, spinal cord; FA, fractional anisotropy; MD, mean diffusivity; MTR, magnetization transfer ratio; r, Pearson correlation coefficient;*ρ*, Spearman correlation coefficient. All spinal cord measurements were averaged from cervical C2-5 levels. Black dashed regression lines were estimated from the Siemens and Philips scanners’ data points. Red dotted regression lines were estimated from the GE scanner’s data points. Plots with statistically significant correlation (p_FWE_< 0.05) are highlighted with yellow background, and corresponding r and*ρ*values are highlighted with black bold font.

GE-scanner-derived DTI and MTR measurements significantly differed from Siemens and Philips scanners (Fig. 3; p < 0.0001). Therefore, GE scanner microstructural measurements (13.87% of the dataset) were not included in correlation analyses that did not use manufacturer-specific normalized microstructural values (Table 2). Body weight was correlated weakly with mean diffusivity (MD) in the WM region (r = -0.200;Fig. 3;Table 2;Supplementary Fig. S2) and bilateral dorsal columns (DC, r = -0.207;Supplementary Fig. S3). Body weight was not significantly correlated to MD for females (Table 2). No investigated DTI measures (i.e., MD, FA or RD) were correlated to body size when extracted from the GM region (Supplementary Fig. S4) or bilateral lateral corticospinal tracts (LCST;Supplementary Fig. S5). CSA-WM and SC FA were correlated weakly in DC (r = -0.247) and LCST (r = -0.224). Body weight was correlated weakly to MTR in WM (r = -0.225,Fig. 3), DC (r = -0.231;Supplementary Fig. S6), and LCST (r = -0.200;Supplementary Fig. S6), and not correlated to MTR in GM (Supplementary Fig. S6). The correlation between body weight and MTR remained significant, even when the dataset was split into males and females (Table 2). When the dataset was normalized for each manufacturer and values from GE scanners were included in the analysis, the correlation values remained almost identical (Table 2). This finding signifies that the observed effect remained identical but had slightly higher power due to the larger sample size (added 37 samples; +13.87%). The correlation analysis revealed no aging effects in DTI (-0.004 ≥ r ≥ -0.099) or MTR (-0.047 ≥ r ≥ -0.094) measures (Supplementary Figs. S2–S6) in our sample. However, the exploratory principal component analysis showed small effects in mutual covariance (Fig. 7d). Linear regression analysis showed that body weight explained the majority of the demography-related variance in our young adult sample DTI and MTR measurements (Supplementary Table S3).

### 3.5 Body height, ICV, and age interact with brain morphology

**Fig. 4.**
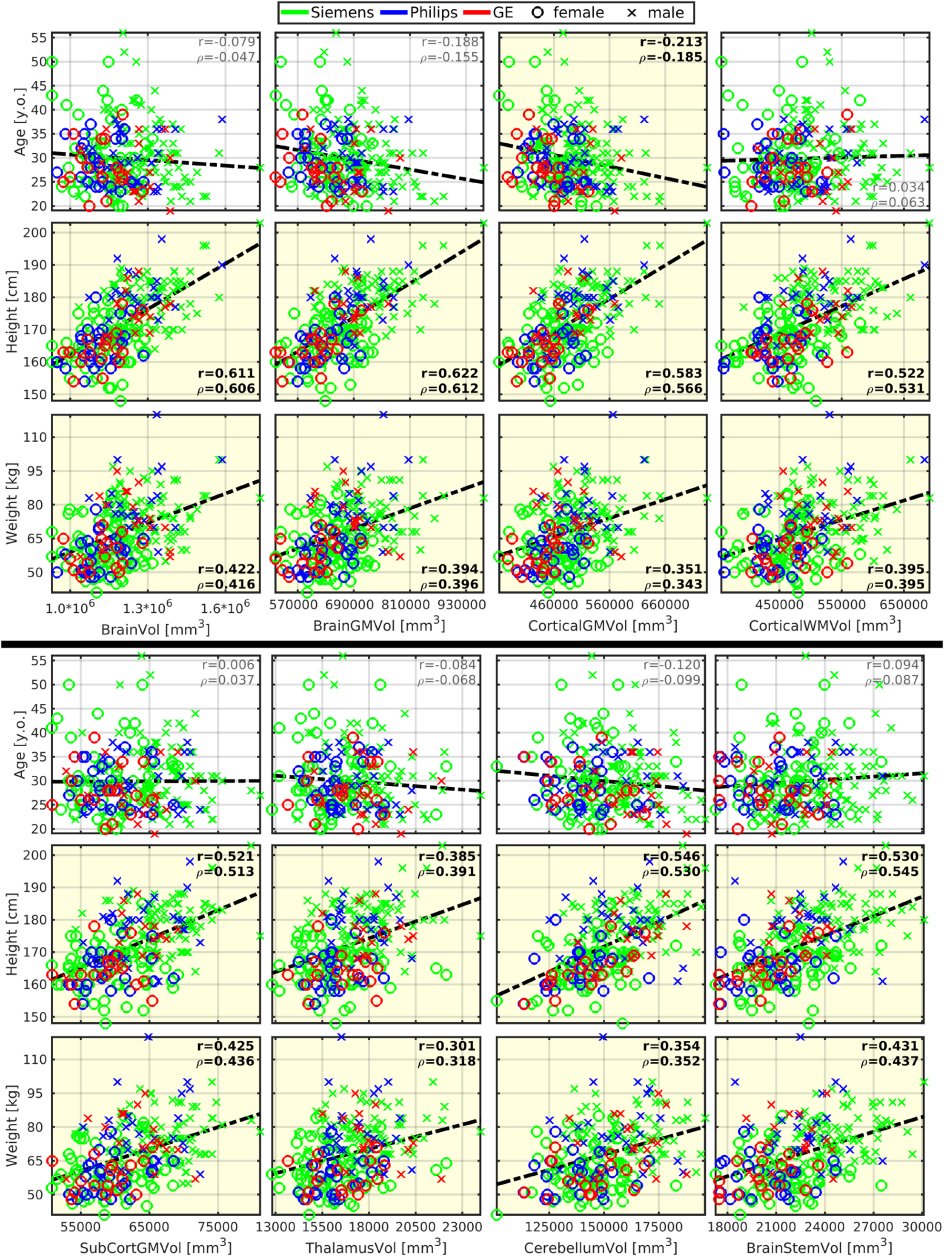
Brain morphology moderately correlates with body size but weakly with age. GM, gray matter; WM, white matter; Vol, volume; SubCort, subcortical; r, Pearson correlation coefficient;*ρ*, Spearman correlation coefficient. Regression lines (i.e., the dashed black lines) were estimated from all available data points. Plots with statistically significant correlation (p_FWE_< 0.05) are highlighted with yellow background, and corresponding r and*ρ*values are highlighted with black bold font.

Body height was correlated moderately with several cerebral volumes (r = 0.54 ± 0.06; 0.434 ≤ r ≤ 0.622), that is, volumes of the brain, brain GM, cortical GM, cortical WM, subcortical GM, thalamus, cerebellum, brainstem, precentral GM, and postcentral GM (Figs. 4and5a). The vast majority of correlations with body height remained significant even after the dataset was split to males and females, except for the volumes of cortical WM, subcortical GM, precentral GM, and postcentral GM in females (Table 2). The body height interacted most profoundly with the cortical GM volume (Fig. 2b).

**Fig. 5.**
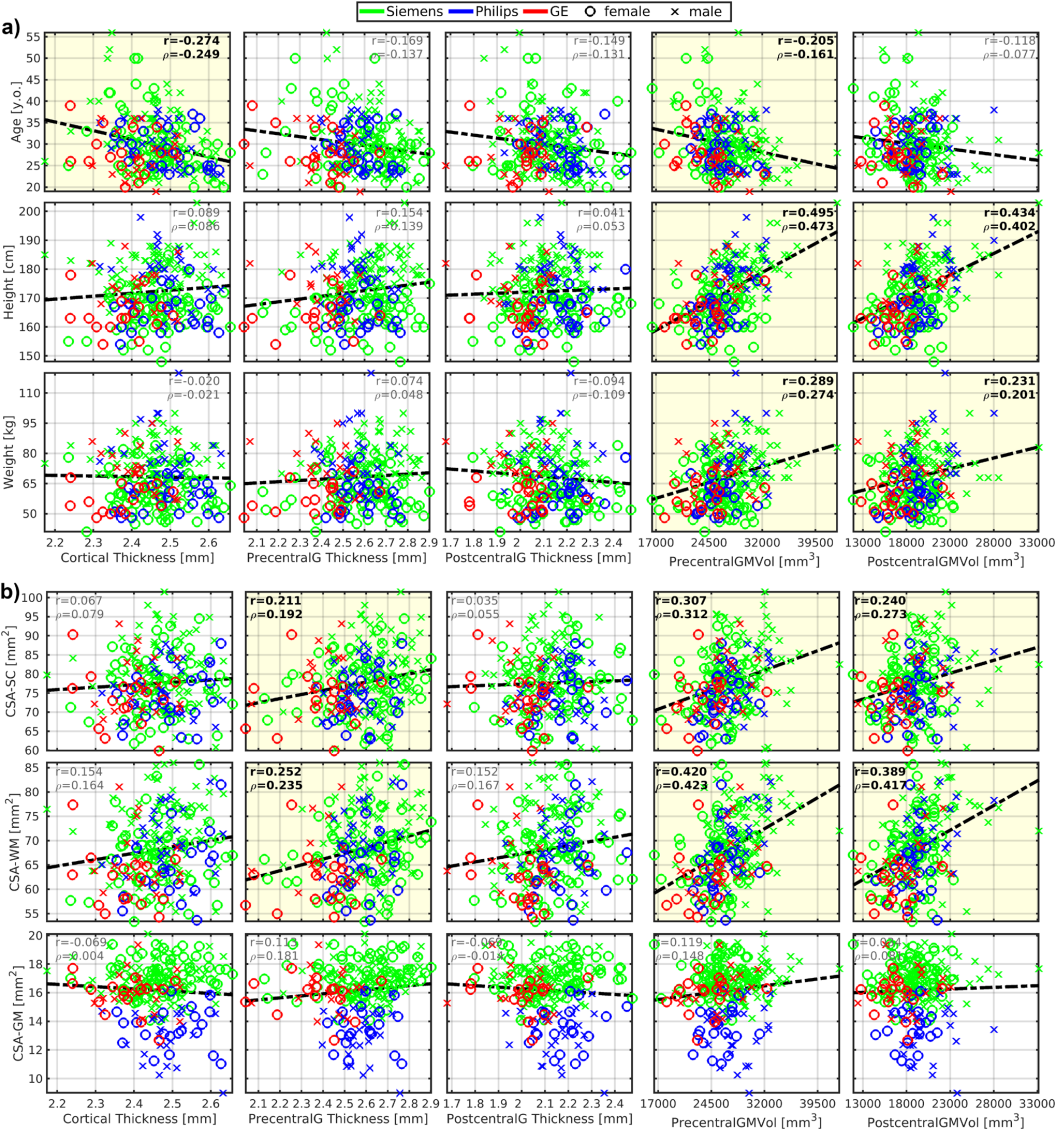
Cortical morphology correlates with body size, age, and cross-sectional area of the spinal cord white matter. CSA, cross-sectional area; SC, spinal cord; WM, white matter; GM, gray matter; PrecentralG, precentral gyrus; PostcentralG, postcentral gyrus; Vol, volume; r, Pearson correlation coefficient;*ρ*, Spearman correlation coefficient. Regression lines (i.e., the dashed black lines) were estimated from all available data points. Plots with statistically significant correlation (p_FWE_< 0.05) are highlighted with yellow background, and corresponding r and*ρ*values are highlighted with black bold font. (a) Graphs demonstrate correlations with body size and age. (b) Graphs demonstrate correlation with CSA measured in the SC region as averages from cervical C3-4 levels.

Body weight demonstrated weaker correlations in all the cortical regions that correlated moderately with body height (r = 0.37 ± 0.07;Figs. 4and5a;Supplementary Table S2). The only significant correlation that survived the dataset split to males and females was brainstem volume in males (Table 2).

Body height or body weight was not correlated with total cortical, precentral gyrus, and postcentral gyrus thicknesses (Fig. 5a).

ICV correlated with brain morphology more profoundly than body height (Table 2;Figs. 4and5a;Supplementary Fig. S1). Yet, regarding the low-to-moderate correlation coefficients between ICV and body size (Table 2), we assume that ICV and body size do not share entropy entirely. Thus, all effects in brain morphology cannot be explained by ICV only.

As expected, a weak manifestation of age-related cortical GM atrophy was observed in volume (r = -0.213) and thickness (r = -0.274) measures (Figs. 4and5a). The aging GM atrophy effects remained significant after the dataset split to males and females (Table 2).

Most importantly, the magnitude of linear dependence between brain morphology and body height (or ICV) was about two- to threefold compared with the effects of age (Table 2,Figs. 4and5a). Moreover, the young adult dataset showed that body height and ICV explain more, pathology unrelated, variance in brain volumetry than age and sex (Supplementary Table S3). Contrary, cortical thickness variance was associated predominantly with age (Supplementary Table S3).

### 3.6 Body size or concurrent body mass measurements?

BMI was highly linearly dependent on body weight (*r_BMI_*= 0.801). BSA and LBW were highly linearly dependent on body height (*r_BSA_*= 0.864;*r_LBW_*= 0.867) and weight (*r_BSA_*= 0.964;*r_LBW_*= 0.933), favoring effects of the weight against the height in the final measurement. Therefore, neither BMI, BSA, and LBW demonstrated higher linear dependence effects with CNS morphology than were observed for the body height (Supplementary Figs. S7–S9vs.Figs. 1;4and5a). BMI, BSA, and LBW do not seem to increase correlation magnitude with SC MTR when compared with body weight (Supplementary Fig. S10vs.Fig. 3). However, LBW may increase the correlation magnitude with SC DTI (Supplementary Fig. S10vs.Fig. 3).

### 3.7 Body size and ICV improve prediction of CNS structure

Linear regression of age itself explained only 2 ± 2% of investigated CNS morphology variance (Supplementary Table S3). Utilizing body height (R^2^= 27 ± 8%), sex (R^2^= 24 ± 10%), or ICV (R^2^= 36 ± 16%) separately explained a significant portion of variance in CNS morphology (Supplementary Table S3). However, the amount of explained variance in CNS morphology was maximized when a linear mixture of all four variables was modeled together (R^2^= 46 ± 17%;Supplementary Table S3). Stepwise linear regression identified age, body height, and ICV as significant variables determining investigated CNS morphological measurements (R^2^= 45 ± 17%;Table 3). Sex was an additional significant variable in predictions of cerebellar, brainstem, and subcortical GM volumes (Table 3). Pearson correlation coefficient between measured and predicted CNS morphology increased from r = 0.58 ± 0.15 to r = 0.66 ± 0.13 when compared with ICV correlations (Table 3).*Utilizing male dataset only,*the identified set of significant variables predicting CNS morphology remained very similar. Additionally, body weight was identified as an additional variable in some measurements. Explained CNS morphology variance was R^2^= 38 ± 18% and the Pearson correlation coefficient increased from r = 0.52 ± 0.16 to r = 0.60 ± 0.16 when compared with ICV correlations (Supplementary Table S4).*Utilizing female dataset only,*the identified set of significant variables predicting CNS morphology remained very similar to male and both sex models. However, the explained variance was only 20 ± 9% and the correlation coefficient increase was from r = 0.31 ± 0.15 to r = 0.43 ± 0.11, suggesting unidentified biological factor/s further determining the CNS morphology in young adult females (Supplementary Table S5).

**Table 3.** Stepwise linear regression fitted models predicting CNS structural measure (y).

Fitted model: y ∝ y _0_ + ∑ b _i_ *x _i_	* R ^2^*	* R ^2^_ICV_*	* R ^2^_Height_*	*r*	* r _ICV_*	RMSE	*y (mean ± STD)*
***BrainGMVol*** ∝ 42252 -1943*Age +2593*Height +0.164*ICV	63.6%	49.4%	38.7%	0.797	0.703	42399 mm ^3^	673668 ± 70031 mm ^3^
***BrainVol*** ∝ -35072 +4215*Height +0.354*ICV	68.0%	59.8%	37.4%	0.824	0.773	73122 mm ^3^	1213993 ± 128729 mm ^3^
***CorticalGMVol*** ∝ 35408 -1778*Age +1834*Height +0.13*ICV	59.7%	46.7%	34.0%	0.772	0.684	35417 mm ^3^	490600 ± 55570 mm ^3^
***CerebellumVol*** ∝ 48271 -348*Age +446*Height +0.025*ICV +6953*Sex	44.4%	30.9%	29.8%	0.666	0.556	13028 mm ^3^	151930 ± 17344 mm ^3^
***BrainStemVol*** ∝ 5814 +71.6*Height +0.003*ICV +1069*Sex	38.5%	25.0%	28.1%	0.620	0.500	2049 mm ^3^	22613 ± 2597 mm ^3^
***CorticalWMVol*** ∝ -3594 +1465*Height +0.165*ICV	56.9%	52.2%	27.2%	0.754	0.723	40055 mm ^3^	491753 ± 60605 mm ^3^
***SubCortGMVol*** ∝ 29015 +91*Height +0.012*ICV +2474*Sex	53.0%	45.3%	27.2%	0.728	0.673	3989 mm ^3^	62987 ± 5804 mm ^3^
***PrecentralGMVol*** ∝ 1702 -111*Age +114*Height +0.0061*ICV	39.1%	27.5%	24.5%	0.625	0.524	2865 mm ^3^	27103 ± 3641 mm ^3^
***PostcentralGMVol*** ∝ 1217 -50.5*Age +67.3*Height +0.0054*ICV	33.8%	27.6%	18.8%	0.582	0.526	2344 mm ^3^	19197 ± 2858 mm ^3^
***CSA-WM*** ∝ 14.23 +0.242*Height +8e-06*ICV	25.6%	17.1%	17.8%	0.506	0.370	6.2 mm ^2^	68.1 ± 7.4 mm ^2^
***CSA-SC*** ∝ 29.88 +0.224*Height +6e-06*ICV	15.5%	9.7%	11.8%	0.394	0.300	7.4 mm ^2^	77.4 ± 8.0 mm ^2^
***MD-SC-WM*** ∝ 1.030 -0.0012*Weight	4.3%	0.3%	2.2%	0.252	-0.015	0.08*10 ^-9^ m ^2^ /s	(0.95 ± 0.13)*10 ^-9^ m ^2^ /s
***MTR-SC-WM*** ∝ 50.3 -0.037*Weight	5.1%	1.5%	0.8%	0.221	-0.116	2.1%	47.5 ± 3.7%
***Cortical Thickness*** ∝ 2.57 -0.0037*Age	7.6%	0.2%	0.8%	0.274	0.042	0.08 mm	2.46 ± 0.09 mm

ICV, intracranial volume; CSA, cross-sectional area; SC, spinal cord; WM, white matter; GM, gray matter; Vol, volume; SubCort, subcortical;***y***, CNS measured structure;*y_0_*, model constant member (intersect);*b_i_*, regression coefficient of*i-th*variable*x*;*x*, regressed significant variable (e.g., Height, ICV, etc.); R^2^, coefficient of determination for the stepwise fitted model; R^2^_ICV_,- coefficient of determination for fitted linear regression model utilizing sex, age, and ICV variables; R^2^_Height_, coefficient of determination for fitted linear regression model utilizing sex, age, and body height variables; r, Pearson correlation coefficient between measured***y***and stepwise model predicted***y***;*r_ICV_*, Pearson correlation coefficient between measured***y***and ICV; RMSE, root mean square error between measured***y***and stepwise model predicted***y***; STD, standard deviation.

All variables listed in the fitted models met the statistical threshold condition p < 0.05. In all cases, the stepwise linear regression fitted model explained more data variance than concurrent linear mixture model utilizing sex, age, and ICV (*R^2^_ICV_*); or sex, age, and body height (*R^2^_Height_*), respectively. Coefficients of determination for other investigated mixture models are listed in theSupplementary Table S3. Pearson correlation coefficient also increased for the stepwise fitted model when compared with correlation levels with ICV (*r_ICV_*) or body height (Table 2) separately.

Body weight explained ≈5% variance in SC microstructure measured with DTI or MTR (Table 3;Supplementary Tables S3–S5). When the dataset was split into males or females only, body weight was not identified as a significant variable determining DTI (Supplementary Tables S4 and S5). Age explained ≈8% variance in cortical thickness measurements (Table 3;Supplementary Tables S3–S5).

CNS structural measurements and root mean square errors (RMSE) of all model predictions are listed inTable 3andSupplementary Tables S4 and S5. In all cases, the RMSE was lower than standard deviation of the structural measurement (Table 3;Supplementary Tables S4 and S5).

### 3.8 Cross-sectional area of spinal cord white matter interacts with brain morphology

**Fig. 6.**
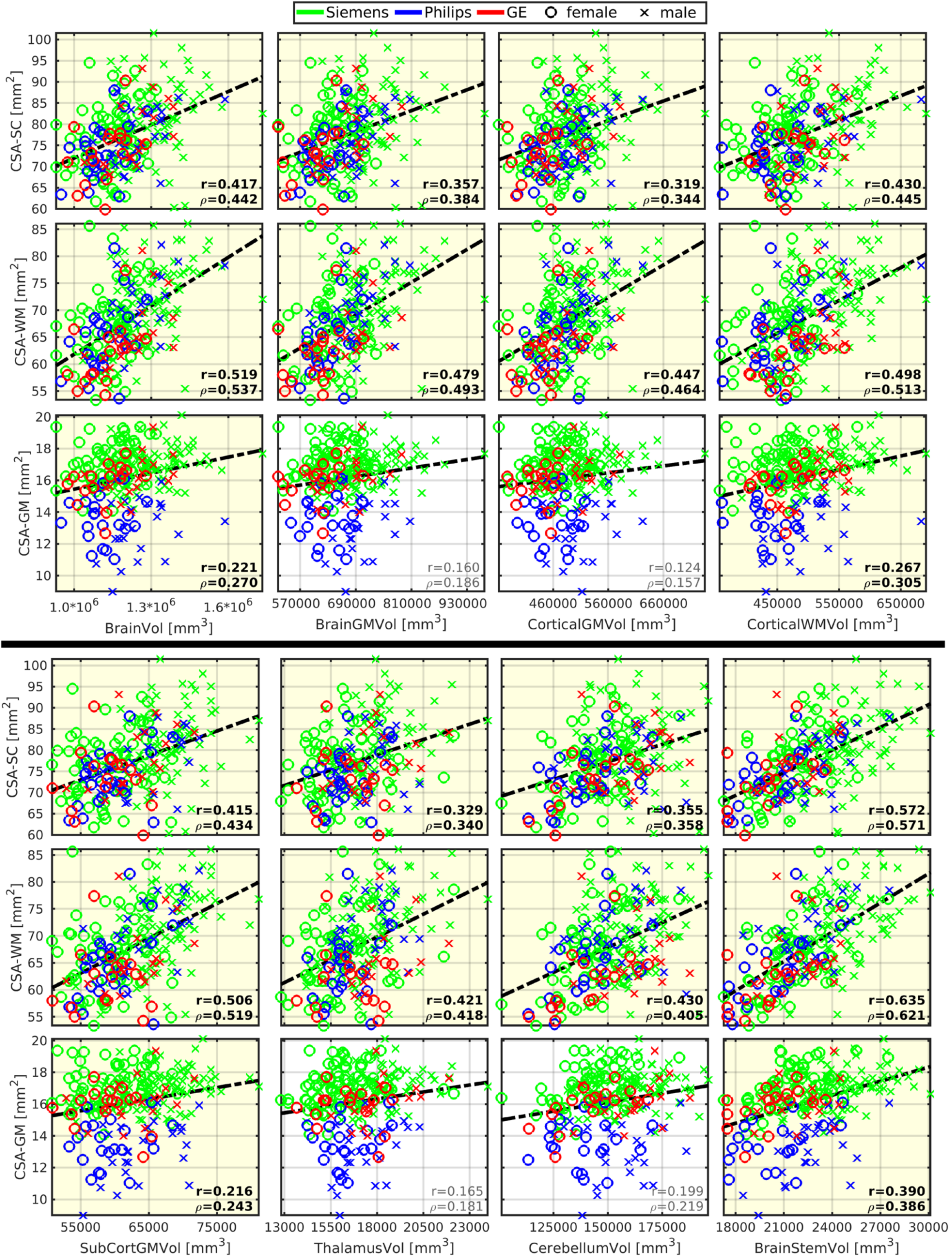
Brain morphology correlates with spinal cord morphology. CSA, cross-sectional area; SC, spinal cord; WM, white matter; GM, gray matter; Vol, volume; SubCort, subcortical; r, Pearson correlation coefficient;*ρ*, Spearman correlation coefficient. All SC measurements were averaged from cervical C3-4 levels. Regression lines (i.e., the dashed black lines) were estimated from all available data points. Plots with statistically significant correlation (p_FWE_< 0.05) are highlighted with yellow background, and corresponding r and*ρ*values are highlighted with black bold font.

CSA-SC (r = 0.38 ± 0.09; 0.240 ≤ r ≤ 0.575) and CSA-WM (r = 0.48 ± 0.07; 0.389 ≤ r ≤ 0.640) were correlated moderately with the investigated brain volumes, that is, volumes of the brain, brain GM, cortical GM, cortical WM, subcortical GM, thalamus, cerebellum, brainstem, precentral GM, and postcentral GM (Figs. 5band6;Table 2). Compared with CSA-SC, correlation strengths were higher for CSA-WM (Figs. 5band6;Table 2). CSA-GM was correlated weakly with the volume of the brain, cortical WM, subcortical GM, and brainstem, but the strength of these correlations was half weaker than those observed for CSA-SC and CSA-WM (Fig. 6;Table 2). All CSA-WM and most of the other observed correlations remained significant after the dataset split to females and males (Table 2) or when SC data were normalized (zero mean) for each manufacturer prior to correlation analysis (Table 2). CSA-WM was the primary marker defining the correlations with the brain volumes. There was a descending gradient of the CSA-WM correlation from the brainstem to subcortical GM and then cortical WM to the cortical GM (Fig. 2c). All these correlations were higher than the correlation with the volume of the cerebellum (Fig. 2c). Yet, even the correlation between CSA-WM and cerebellum volume was significant (Figs. 2cand6).

CSA-SC (r = 0.211) and CSA-WM (r = 0.252) were correlated weakly with the thickness of the precentral gyrus (Fig. 5b). The correlations remained significant after the dataset split to females and males (Table 2). However, the correlations disappeared when SC data were normalized before correlation analysis (Table 2). CSA-GM was not correlated with any utilized cortical thickness measurement (Fig. 5b).

### 3.9 Brain morphology and spinal cord microstructure are not related

No correlations were detected between SC WM/GM microstructure and cerebral volumes (i.e., total brain, brain GM, cortical GM, cortical WM, subcortical GM, thalamus, cerebellum, brainstem, precentral GM, and postcentral GM) or cortical thickness (Supplementary Figs. S11–S12), and between thickness measurements and DTI/MTR measurements, even if the SC ROIs were limited to the bilateral LCST or DC (Supplementary Figs. S13–S15).

### 3.10 ICV normalization of brain volumes emphasizes ICV information in the measurements

ICV normalization of volumes of different brain regions reduced correlation levels with body height and weight (Supplementary Fig. S16vs.Figs. 4and5a). But the normalization also reduced variance/entropy about the brain structure itself in the measurement because the normalized brain volumes (*x_norm_*) correlated more strongly with ICV^-1^(r = 0.72 ± 0.07; min 0.58; max 0.83;Supplementary Fig. S16) than with original volumes (*x*; r = 0.12 ± 0.19; min -0.14; max 0.42). Moreover, the ICV normalization emphasized scanner effects in the brain volume measurements. ICV normalization of brain volumes generated a distinct cluster of GE measurements (Supplementary Fig. S16) that was not observed in non-normalized brain volume measurements (Figs. 4and5a). Both brain volumes and ICV were measured with FreeSurfer v7.2. In 14 (8.9%) Siemens and 4 (8.2%) Philips T1w scans, ICV was estimated lower than the unnormalized brain volume. Therefore, the Freesurfer software provided unphysiological normalized brain volumes >100% in such scans (Supplementary Fig. S16). The utilized brain and intracranial volume variables are called BrainSegVol and eITV in the FreeSurfer software. Further detail origin of such software error is unknown to us at the moment. Image visual inspection did not identify any obvious pitfall in these 18 cases.

### 3.11 Scanner-related effects on SC structural measurements

CSA-SC and CSA-WM offsets differed minimally between manufacturers (Figs. 1;5band6). CSA-GM measurements on Philips scanners were significantly lower than CSA-GM measurements from Siemens and GE scanners (Figs. 1;5band6; p < 0.0001). Additional discussion about this specific CSA-GM issue can be found inCohen-Adad et al. (2021b). Data normalization before correlation analysis mainly decreased the correlation strengths in all CSA measures (without normalization: r = 0.348 ± 0.127; with normalization r = 0.313 ± 0.128;Table 2; paired t-test p < 0.0001). This finding underlines the importance of adjusting for scanner-related variability in CSA measurements to minimize risks of false positive results due to scanner-related data trends.

All microstructural measurements obtained with GE scanners showed significant offsets compared with those from Siemens and Philips scanners (Fig. 3;Supplementary Figs. S2–S6 and S10–S15; p < 0.0001). The differences had a direct impact on correlation analyses. Therefore, we performed correlation analyses of original values without GE values and correlation analyses of normalized values utilizing all scanners’ data. Correlation analyses were stable and comparable in the magnitude of correlation coefficients for MTR (Table 2) and MD (Table 2). The normalized correlation analysis provided higher statistical power due to the larger sample size. Additionally, if we utilized GE data (Fig. 3) in the correlation analysis without normalization, the resulting correlation coefficients for MD-SC-WM and MTR-SC-WM given inTable 2would be substantially lower.

### 3.12 Minimal impact of degenerative cervical spinal cord compression on correlation analysis

We excluded one participant with severe degenerative cervical SC compression that introduced outliers in SC structural measurements. However, the*spine-generic*database identifies an additional 61 subjects with mild degenerative compression and 2 subjects with severe degenerative compression and radiological myelopathy (Valošek et al., 2024). Analysis power decreased, but minimal nuances were detected in correlation coefficients when tested separately on subjects without or with degenerative cervical SC compression (seeSupplementary Slides). Thus, we conclude that SC compression had a minimal impact on the current study outcomes.

### 3.13 Principal component analysis (PCA) reveals body–SC–brain structural links

**Fig. 7.**
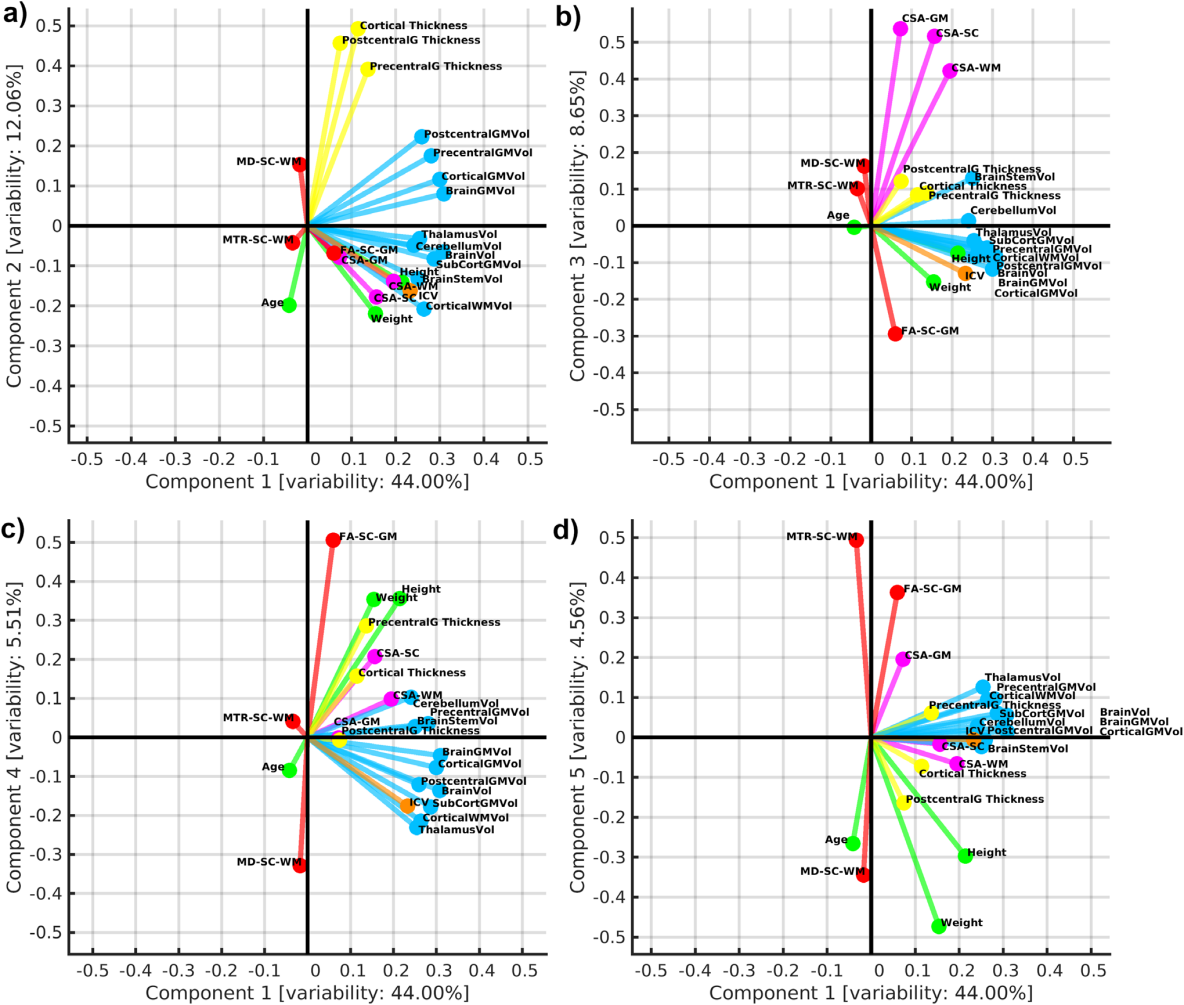
Exploratory visualization using biplot projections of principal components. (a) Biplot projection of 1^st^and 2^nd^principal components (PCs); (b) biplot projection of 1^st^and 3^rd^PCs; (c) biplot projection of 1^st^and 4^th^PCs; (d) biplot projection of 1^st^and 5^th^PCs. Variable vectors are visualized in each biplot projection with a color-coding characteristic for a corresponding variable group. Variable name abbreviations and variable color codings are described as follows.*Variable abbreviations:*ICV, intracranial volume; MD, mean diffusivity; RD, radial diffusivity; MTR, magnetization transfer ratio; SC, spinal cord; WM, white matter; GM, gray matter; CSA, cross-sectional area; Vol, volume; PrecentralG, precentral gyrus; PostcentralG, postcentral gyrus.*Variable color coding:*ICV, orange; demography, green; cerebral volumes, light blue; cortical thicknesses, yellow; SC morphometry, magenta; SC WM microstructure, red.*How to read a biplot:*The overall domain of each component axis is <-1,1>. Each variable is characterized as a vector of magnitude in the range of <0,1> in the biplot space. Angle 0° between the component axis and variable vector with magnitude 1 (or between two variable vectors both with magnitude 1) is proportional to Pearson correlation coefficient 1. Under the same vector magnitude circumstances, an angle of 180° equals Pearson correlation coefficient -1, and angles of 90° and 270° equal Pearson correlation coefficient 0. The lower magnitude of variable vectors proportionally decreases the overall linear dependence between vector angles close to 0° or 180°, respectively. Similarly, angle deviation from 0° or 180° also decreases the level of linear dependence between pairs of vectors in the biplot.

We subtracted manufacturer-specific average values from all SC structural measurements prior to the exploratory analysis via PCA. PCA did not include DTI and MTR measurements from bilateral LCST and DC, as the WM region provided analogic observations. Cerebral volumes, CSA-WM, body height, and ICV formed the first principal component (PC1), characterizing 44.00% of data variance (Fig. 7a). CSA-SC and body weight were close, yet separated from the PC1 cluster (Fig. 7a). This finding supports the previously observed body height and CSA-WM dominance in the observed effects (Figs. 1–2and4–6;Tables 2and3). Cortical thickness, MD-SC-WM, and age (negative effect) formed the second principal component (PC2), characterizing 12.06% of data variance (Fig. 7a) and presenting predominantly negative aging effects in the thickness measures. The PC1-PC3 projection showed that PC3 characterizes about 8.65% of data variance, predominantly explained by CSA-GM, MD-SC-WM, and FA-SC-GM (negative effect), that is, a link between SC GM morphology and SC microstructure (Fig. 7b). The PC2-PC3 projection verified that the cortical thickness variability predominantly forms PC2. In contrast, PC3 is predominantly formed by SC DTI and CSA-GM (Supplementary Fig. S17a). PC4 explained 5.51% of unique SC DTI microstructural data variance, which is not present in other investigated modalities and investigated demographic measures (Fig. 7c). PC5 showed positive effects of body weight and age on MD-SC-WM, and negative effects of body weight and age on MTR-SC-WM, FA-SC-GM, and CSA-GM. These effects explained about 4.56% of data variance (Figs. 3and7d). Simultaneously, the PC projections suggest that the impact of body weight on MTR- and DTI-derived microstructure metrics might be ≈5% (Figs. 3and7). That follows the result of explained variance in the regression analyses (Table 3;Supplementary Table S3). However, the positive effects of body weight on MD-SC-WM contradict our observation of weak negative correlation between body weight and SC MD (Fig. 3). PC3 and PC5 showed clear evidence that CSA-GM morphology and SC microstructure are linked, yet unrelated to cerebral and SC WM morphology (Fig. 7b, d;Supplementary Fig. S17a). In summary, PCA explained 75% of data variance, and roughly 25% is unexplained (Supplementary Fig. S17b).

## 4 Discussion

The current study, using the *spine-generic* dataset, presents unique multi-center in vivo evidence about adult human cervical SC and brain, and emphasizes the following findings:

***(i)*** Body height correlates moderately with SC WM and brain morphology, improves explanation of demography-related variance in such structural measurements from 26 ± 10% (range 6–37%) to 33 ± 11% (range 12–46%) in young adults, and underlines the impact of such pathology unrelated variability in structural neuroimaging data. When ICV is added into the morphology modeling, the explained variance increases to 46 ± 17% (range 16–69%).
***(ii)*** The expected aging effects (Bédard & Cohen-Adad, 2022;Fjell et al., 2013;Heymsfield et al., 2009;Papinutto et al., 2020;Peters, 2006;Thambisetty et al., 2010) explain minimal amounts of SC and brain structural data variance (2 ± 2%) in young adults except cortical thickness (8%).
***(iii)*** Body height predominantly impacts cortical GM volume (Fig. 2b) and may even define overall brain GM volume.
***(iv)*** Body weight correlates weakly with SC WM MTR, which is influenced by myelin content.
***(v)*** Body weight correlates weakly with SC WM microstructure assessed with DTI MD.
***(vi)*** Body weight explains ≈5–7% of DTI and MTR data variance.
***(vii)*** SC WM DTI and MTR explain a significant portion of examined dataset variance (≈14–19%) and are nearly orthogonal to most macrostructural measurements, except for the CSA-GM.
***(viii)*** Subcortical and cortical GM volumes are correlated with CSA-WM more profoundly than the cerebellar volume with a descending correlation gradient from the brainstem toward cortical GM (Fig. 2c).
***(ix)*** Cortical WM, subcortical GM, and brainstem volumes correlate with CSA-GM but much less profoundly than CSA-WM.
***(x)*** Cortical thickness of the precentral cortex correlates weakly with CSA-WM.
***(xi)*** We highlight the importance of considering the scanner-related effects present in SC imaging data (Cohen-Adad et al., 2021a,2021b).
***(xii)*** We confirm significant relationships between body size, brain volumes/weight, and CSA-SC in line with previously reported results (Baaré et al., 2001;Bédard & Cohen-Adad, 2022;Kameyama et al., 1994).
***(xiii)*** We confirmed strong ICV effects on brain morphology (Miller et al., 2016). Moreover, we demonstrated interactions between ICV and CSA-WM and that utilizing both ICV and body height can maximize the amount of explained variance in CNS morphology (i.e., brain volumetry and CSA measurements in the spinal cord).
**(xiv)** We showed that ICV normalization of brain volumes amplifies ICV-related variance/entropy in all tested regions of interest. Moreover, the normalization emphasized scanner effects.
**(xv)** Females showed a consistently lower level of association with the variables of interest compared with males, and thus also a lower predictive power of the tested linear mixture models to the brain and SC morphology.

### 4.1 Practical impact of the current study in clinical neuroimaging study designs

MRI of SC structure is emerging in clinical research of neurodegenerative diseases and SC injuries (Badhiwala et al., 2020;David et al., 2019;de Albuquerque et al., 2017;Fatemi et al., 2005;Hernandez et al., 2022;Huffnagel et al., 2019;Lema et al., 2017;Lukas et al., 2013;Pisharady et al., 2020;Querin et al., 2017;Schmierer et al., 2004;van de Stadt et al., 2020). Microstructural SC MRI of neural tissue integrity aims to understand pathophysiological changes at the subclinical or presymptomatic stage (Joers et al., 2022;Labounek et al., 2020;Martin et al., 2018;Valošek et al., 2021). Quantitative MRI has made significant advances over the past two decades for brain imaging (Ahn et al., 2019;Anik et al., 2007;Appelman et al., 2009;Asken et al., 2018;Aylward et al., 2000;Benatar et al., 2022;Fox et al., 1996;Ginestroni et al., 2009;Hall et al., 2008;Hayakawa et al., 2013;Kabani et al., 2002;Kinnunen et al., 2018;Masuda et al., 2022;Reading et al., 2005;Ringman et al., 2007;Rosano et al., 2005,^2010^;Rosas et al., 2006;Rovira et al., 2001;Wade et al., 2008), but is still in its early development stage when it comes to SC imaging. Sex matching and age matching are critical for any clinical neuroimaging study. Yet, we are proposing that mismatched variability in body size may influence imaging outcomes more profoundly than mismatched variability in age. Persistent marginal impact of body stature on brain structural and functional neuroimaging outcomes in the early elderly population (Alfaro-Almagro et al., 2021;Miller et al., 2016) further underlines the importance of our proposal. Therefore, body size needs to be considered in the rigor of future neuroimaging studies focusing on between-group differences in brain or SC structure to secure and guarantee the reproducibility of results. It has not been a common practice in design of the vast majority of current clinical studies focusing on brain or SC neuroimaging. An alternative solution in future clinical study designs can be normalizing structural measurements for body size or using body size as a confounding factor. In brain volume measurements, for example, SIENAX (Smith et al., 2002) or other kinds of normalization for the total ICV may offer an effective normalization method that provides reproducible results independent of body size. In the SC morphology, SIENAX (Papinutto et al., 2020) or the dimension of pontomedullary junction (Bédard & Cohen-Adad, 2022) have been implemented to normalize the CSA measurement. Yet, if possible, we conclude that body size matching provides a more optimal study design solution because we showed that body size characterizes a significant portion of CNS structural information that is not characterized by the ICV. Simultaneously, recruitment of body size matched participants should be an easier clinical design task than to utilize ICV matching.

### 4.2 Body size, sex, neuroimaging, and CNS (patho-)physiology

Body height had the highest impact on brain GM and SC WM morphology. Body height, higher cortical volume, and improved cognitive ability appears to be phenotypically interlinked (Vuoksimaa et al., 2018). The higher brain GM volumes in taller people may also explain their higher resistance to Alzheimer’s disease and other dementias (Daghlas et al., 2023;Jørgensen et al., 2020;Larsson et al., 2017). Gene expression could play a role here, as genetic variants that affect height also influence brain development and biological pathways that are involved in the development of Alzheimer’s disease (Larsson et al., 2017).

Although our data showed an insignificant interaction between body weight and CNS morphology after controlling for sex, body weight is known to influence CNS morphology and microstructure. Varying body weight showed WM and GM brain volume loss in patients with acute anorexia nervosa, and full WM volume and almost complete GM volume recoveries after the body weight had been regained (Seitz et al., 2014). In the opposite body weight spectrum, obesity demonstrated lower intra-cortical myelination in regions involved in reward processing, attention, salience detection, and higher intra-cortical myelination in regions associated with somatosensory processing and inhibitory control (Dong et al., 2021). High cumulative BMI is associated with smaller brain volume, larger volume of white matter lesions, and abnormal microstructural integrity (Lv et al., 2024;Ward et al., 2005;West et al., 2020). Increasing BMI changes cerebral WM microstructure assessed with DTI (Kullmann et al., 2016), but direction of DTI parameter trends in relation to body weight varies between studies (Okudzhava et al., 2022). Although precise pathophysiological processes are not well known today, it is certain that obesity causes neuroinflammation, thus, alters brain microstructure and increases risks of neurodegenerative disorders such as Alzheimer’s disease and other types of dementias (Woo et al., 2022). Our DTI and MTR data acquired in the current healthy population with low-to-moderate BMI may point to a borderline trend between homeostasis and mild microstructural changes related to higher body weight. The negative correlation between body weight and MTR has also recently been reported in peripheral nerves and skeletal muscles (Fösleitner et al., 2022). However, we cannot rule out the possibility of a transmit field (i.e., B1+) inhomogeneity-mediated bias in MTR. Although B1+ maps were not measured for the cervical SC in our study, similar to what has been observed in the brain at 3T (Glasser et al., 2022), we expect both B1+ inhomogeneity and deviation to correlate with body weight positively, hence body transmit coil loading. Typically, an underflipping (i.e., reaching smaller than the desired flip angle) is more likely than an overflipping for small structures like the cervical SC in the body. MTR’s sensitivity to B1+ potentially exacerbates the effect of even a small degree of underflipping for the MT pulse at 3T.

Body height and spinal cord length are linearly dependent (r ≈ 0.6) (Fradet et al., 2014;Zyoud et al., 2020). We showed that even CSA-SC and CSA-WM are linearly dependent with body height. Thus, the magnitude of the correlation with body height would be even higher than observed for the CSA measurements if level-specific SC and SC WM volumes were analyzed. Although CSA values are level dependent (Cohen-Adad et al., 2021a), the impact of the C3-4 level selection on general study conclusions should remain minimal due to high intra-individual CSA correlation over segments (Healy et al., 2012;Kameyama et al., 1996). Different associations of CSA-GM and CSA-WM with other investigated variables may affirm the necessity of further development of MRI protocols imaging SC GM in high contrast and detail (Cohen-Adad et al., 2022).

Recently, the correlation between CSA-SC at C2-3 level and body height, body weight, brain (WM/GM) volumes, and thalamus volume was observed in 804 UK Biobank brain imaging database participants (Bédard & Cohen-Adad, 2022). Our current*spine-generic*database study complements the UK Biobank results and expands the knowledge that these observations are almost exclusively SC WM related. Moreover, the current study identified more cerebral sub-regions involved than those investigated in the previous study. The lateral corticospinal tracts predominantly serving motor function are the major portion of the CSA-WM (Chandar & Freeman, 2014). Thus, its significant correlation with precentral gyrus thickness (primary motor cortex) seems logical from a neuroanatomical perspective. SC microstructure was also investigated and our exploratory approach via PCA clearly visualizes the body–SC–brain structural relationships.

Although we showed interactions between CSA-WM and ICV and that ICV can explain variance in SC morphology, it can often be challenging to design a neuroimaging study that measures both parameters. Studies focusing on SC pathology do not often acquire brain images (David et al., 2019;Kadanka et al., 2017;Keřkovský et al., 2012;Labounek et al., 2020;Martin et al., 2017,^2018^;Nouri et al., 2016;Valošek et al., 2021). In case of the ultra-high field MRI (≥7T), it can even be a challenging task as the highly optimized SC imaging coils do not cover the whole brain (Lopez-Rios et al., 2023).

Although clinical studies focusing on cerebral atrophy often normalize distinct brain region volumes with ICV (Voevodskaya et al., 2014;Whitwell et al., 2001;Xie et al., 2005) and we would usually normalize the data too, our cross-sectional results suggest that the normalization magnifies ICV information in such volume measurements. The ICV normalization impact on associations with neurocognitive or behavioral outcomes remains unclear. ICV normalization flips signs of the association with neurocognitive outcomes in dementia, but does not change the overall association conclusion (Konstantinou et al., 2016;Wang et al., 2024). The opposite sign may be an effect of the additional ICV^-1^scaling factor. However, ICV normalization biases volume-based behavioral models (Dhamala et al., 2022). In the*spine-generic*dataset, the Freesurfer provided higher BrainSegVol (brain volume) than eITV (ICV) in 18 scans. The FreeSurfer was reported to overestimate ICV by about 4% due to brain volume bias (Klasson et al., 2018), but that does not explain our observed phenomena that BrainSegVol can be higher than eITV. Future research may assess brain volume and ICV with concurrent tools (Harkey et al., 2022;Manjón & Coupé, 2016;Nerland et al., 2022).

The slightly lower variance in female data may be a cause of the lower predictive power of the utilized linear mixture models to brain and SC morphology. However, we doubt that it would halve the predictive accuracy. Thus, we speculate that unidentified female-specific biological factor(s) further determine females’ CNS morphology. Brain structural organization differs between males and females, potentially due to different hormonal levels and gene expression (Liu et al., 2020). Moreover, a pregnancy increases hormone production and induces long-lasting reversible and irreversible changes in females’ brain structure (Hoekzema et al., 2017;Pritschet et al., 2024). Neither hormonal, genetic, and pregnancy data were collected, thus impossible to test in the models with the current*spine-generic*records.

The*spine-generic*database (*r20231212*) identifies 64 recruited subjects with the presence of degenerative cervical SC compression, with 2 of these even demonstrating radiological signs of myelopathy (Valošek et al., 2024). These findings may represent a source of unexplained variance in our results, as compression and myelopathy are pathologies affecting CSA, DTI, and MTR measures (Kadanka et al., 2017;Labounek et al., 2020;Martin et al., 2018;Valošek et al., 2021). However, we showed in theSupplementary Slidesthat the impact of the compression on the correlation coefficient outcomes was minimal.

The observed negative correlation between age and cortical thickness and absence of correlation between body size and cortical thickness are in line with the literature (Frangou et al., 2022;Sowell et al., 2007;Tamnes et al., 2010;Thambisetty et al., 2010;Vuoksimaa et al., 2018). The GM volume reduction in subcortical structures is less profound than in the cortical GM volume and thickness (Fjell et al., 2013,^2021^;Narvacan et al., 2017). Therefore, we may only detect low, insignificant trends in the age-related reductions of the subcortical structures due to an undersampled elderly population in our dataset. SC CSA-GM is also expected to decline with age (Papinutto et al., 2015), but we observed no such effects. Absent SC GM reduction might imply a false positive result due to the limited spatial resolution of the imaging methods, and the undersampled elderly population. It may also mean that the pathophysiological dynamics of SC GM reduction are slower than in the subcortical region. Yet, validating and concluding any of such statements require a rigorous re-test utilizing a dataset with a larger sample elderly population or longitudinal follow-up.

### 4.3 Study limitations

Despite the relatively large sample size, there are still several limitations. First, we recruited healthy, predominantly young adults with average weight and low-to-moderate BMI. Therefore, the negative link between age and SC morphology, as observable in cohorts with greater age variability (Bédard & Cohen-Adad, 2022;Ishikawa et al., 2003;Papinutto et al., 2015), was absent in our study. We found that body size impacts structural measurements more profoundly than age. However, this finding warrants further investigation, as the moderate age effects may be explained by the relatively narrow age range and younger cohort (Heymsfield et al., 2009). However, concurrent study of 40–69-year-old adults also showed significant impact of body size on brain neuroimaging data (Alfaro-Almagro et al., 2021;Miller et al., 2016). ICV and head size were identified as an effective confounding factor minimizing the body size effects in brain structural measurements (Alfaro-Almagro et al., 2021;Miller et al., 2016). The head size is not possible to measure precisely from the*spine-generic*database, because the images covering the brain were manually defaced by deleting the facial area in images. Thus, a significant portion of the image capturing head is missing in every scan. Yet, we employed the ICV covariate that is highly correlated to the head size and expected to be a more relevant measure for brain-related analyses (Miller et al., 2016). CSA-GM, SC DTI, and SC MTR measurements demonstrated scanner-related variability, which needs to be addressed in multi-center data acquisition and analysis. Data of subjects with very low and high BMI may help to investigate the dependence of MTR and DTI measures on body weight. RF inhomogeneities need to be better mapped in future studies to avoid risks of biases in MTR outcomes. Comparison between SC and cerebral microstructure is impossible with the*spine-generic*database because the database does not contain images of brain microstructure. The current*spine-generic*database version does not allow assessing the impact of socioeconomic and race/ethnicity status on obtained MRI metrics (Piccolo et al., 2016). Relationships between spinal canal area (Fradet et al., 2014), cervical cerebrospinal fluid area (Fradet et al., 2014), and body size have not been investigated. Axial diffusivity (AD, i.e., another DTI metric) was not investigated. We expected that AD would provide similar results as observed for MD and RD due to expected high FA-MD-RD-AD intra-correlation levels; therefore, we decided to shrink the variable space. Overall, we conclude that body height and weight should be sufficient and self-explanatory measures of body size for the current study outcomes. However, future studies should measure LBW more rigorously than has been possible here and determine LBW effects on CNS microstructure. Reliable interpretation and additional value of our LBW and BSA association results may be limited because they were theoretically calculated utilizing body height and weight. The cross-sectional study design limits testing of body size changes on the CNS over time.

## 5 Conclusions

***(i)***We confirmed that*“Future clinical research studies and trials utilizing neuroimaging should include body size as a potential confounding biological factor to avoid bias in clinical outcomes.”**(ii)***We hypothesized that “CSA of cervical SC WM and GM interacts with body size and morphology of distinct brain structures,” but after analysis we refine this to*“CSA of cervical SC WM interacts with body height and morphology of distinct brain structures with a descending gradient from subcortical structures to cortical gray matter.”**(iii)***We hypothesized that “SC microstructure, as measured using MTR and DTI, interacts with body size,” but after analysis we refine this to*“SC WM microstructure, as measured as MD and MTR, interacts with body weight, and more profoundly in dorsal columns than in lateral corticospinal tracts.”*We confirmed our hypotheses that***(iv)**“Cerebral morphology interacts with body height more profoundly than with body weight and age”*and that***(v)**“Body size increases the predictive power of CNS structure.”*

## Supplementary Material

Supplementary Material

Supplementary Slides

## Data Availability

All raw data are publicly available at:https://github.com/spine-generic/data-multi-subject(utilized release ID: r20231212). MRI protocols for all optimized manufacturers and scanner types are publicly available at:https://github.com/spine-generic/protocols. Tables with SCT and Freesurfer measurements are available at:https://github.com/umn-milab/spine-generic-body-size-results(utilized release ID: r20250226). Spinal Cord Toolbox is available at:https://github.com/spinalcordtoolbox/spinalcordtoolbox(utilized version: 6.1; git commit: git-master-c7a8072fd63a06a2775a74029c042833f0fce510). FreeSurfer is available at:https://surfer.nmr.mgh.harvard.edu(utilized version: 7.2). All computer code providing image and statistical analyses is available at:https://github.com/spine-generic/spine-generic(utilized release ID: height-weight-analysis-v1.2).
