# Supplementary material for: Body size and intracranial volume interact with the structure of the central nervous system: A multi-center in vivo neuroimaging study
Source: Imaging Neurosci (Camb). 2025 May 7;3:imag_a_00559. doi: 10.1162/imag_a_00559 (PMC12319740; doi:10.1162/imag_a_00559)
Supplement: Supplementary Material [file imag_a_00559_supp1.pdf]

Supplementary material: **Body size and intracranial volume interact with the structure of the central nervous system: A multi-center in vivo neuroimaging study**

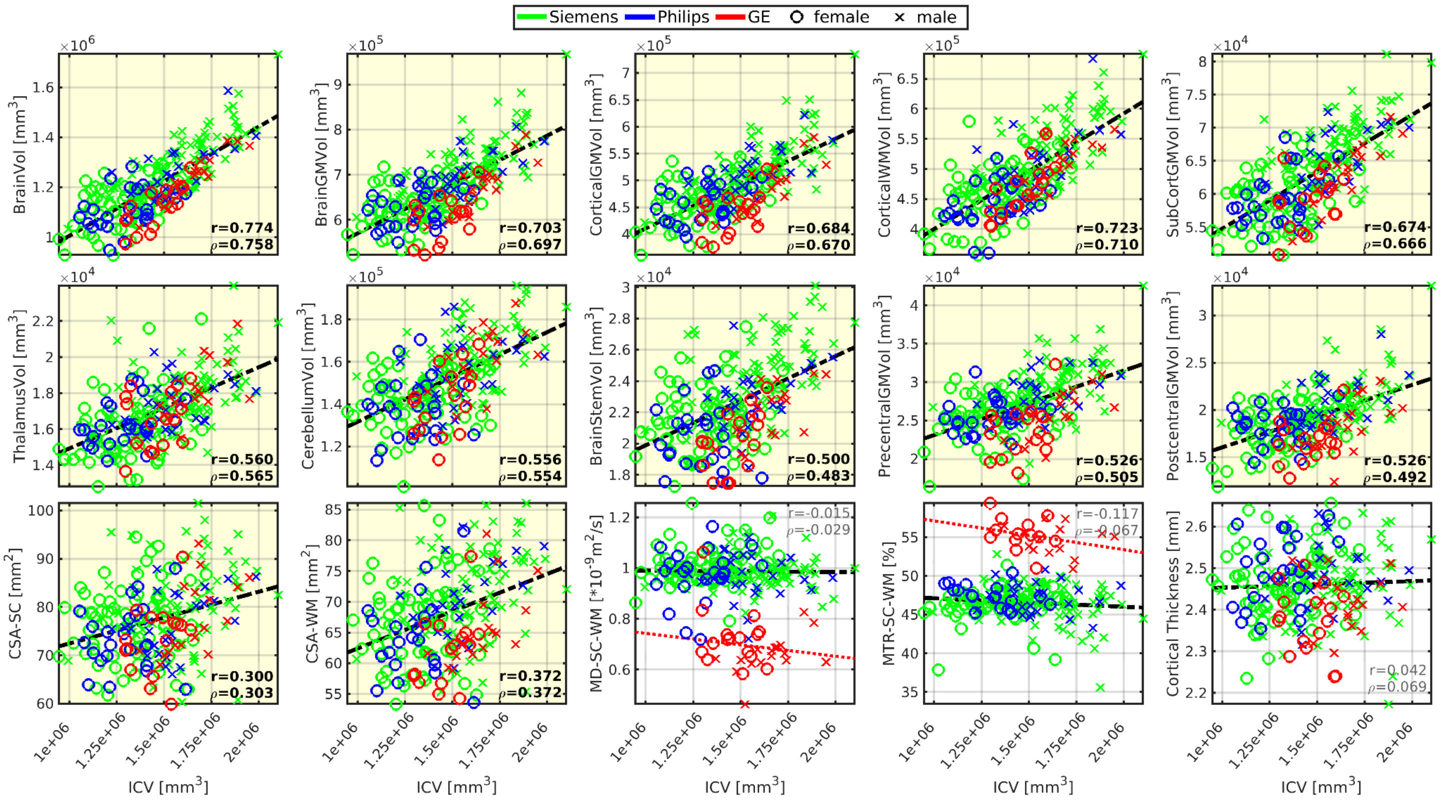

Figure S1: Central nervous system morphology correlates with intracranial volume.

Abbreviations: ICV - intracranial volume; CSA - cross-sectional area; SC - spinal cord; WM - white matter; GM - gray matter; Vol - volume; SubCort - subcortical; MD - mean diffusivity; MTR - magnetization transfer ratio; r - Pearson correlation coefficient; ρ - Spearman correlation coefficient. CSA measurements were averaged from cervical C3-4 levels. MD and MTR measurements were averaged from cervical C2-5 levels. Regression lines (i.e., the dashed black lines) were estimated from all available data points for CNS morphology. Black dashed regression lines were estimated from the Siemens and Philips scanners' data points for MD and MTR. Red dotted regression lines were estimated from the GE scanner's data points. Plots with statistically significant correlation ($p_{FWE}<0.05$) are highlighted with yellow background, and corresponding r and ρ values are highlighted with black bold font.

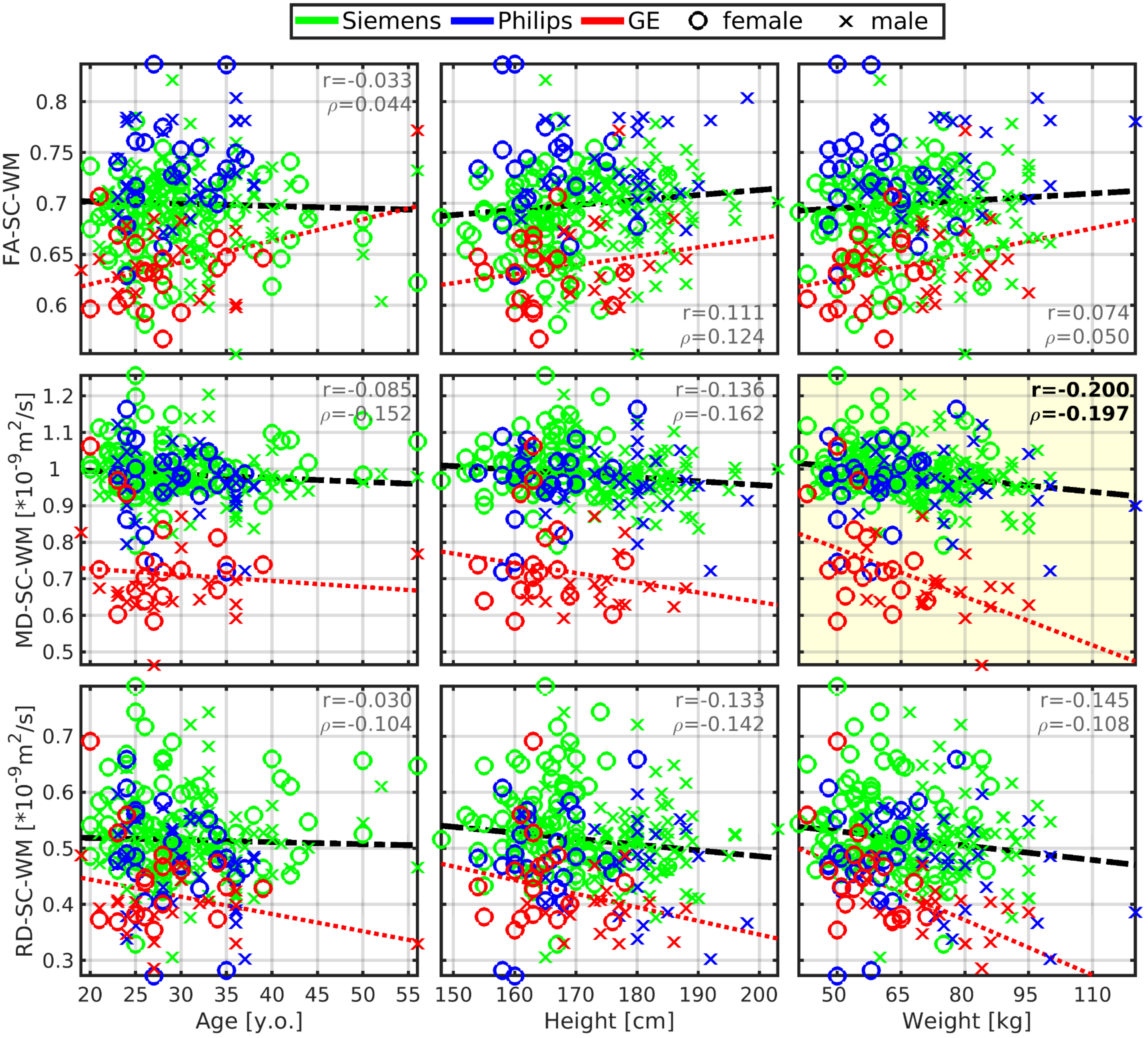

Figure S2: Mean diffusivity in spinal cord (SC) white matter (WM) correlates with body weight.
Abbreviations: WM - white matter; SC - spinal cord; FA - fractional anisotropy; MD - mean diffusivity; RD - radial diffusivity; r - Pearson correlation coefficient; ρ - Spearman correlation coefficient. All spinal cord measurements were averaged from cervical C2-5 levels. Black dashed regression lines were estimated from the Siemens and Philips scanners' data points. Red dotted regression lines were estimated from the GE scanner's data points. Plots with statistically significant correlation (p_{FWE}<0.05) are highlighted with yellow background, and corresponding r and ρ values are highlighted with black bold font.

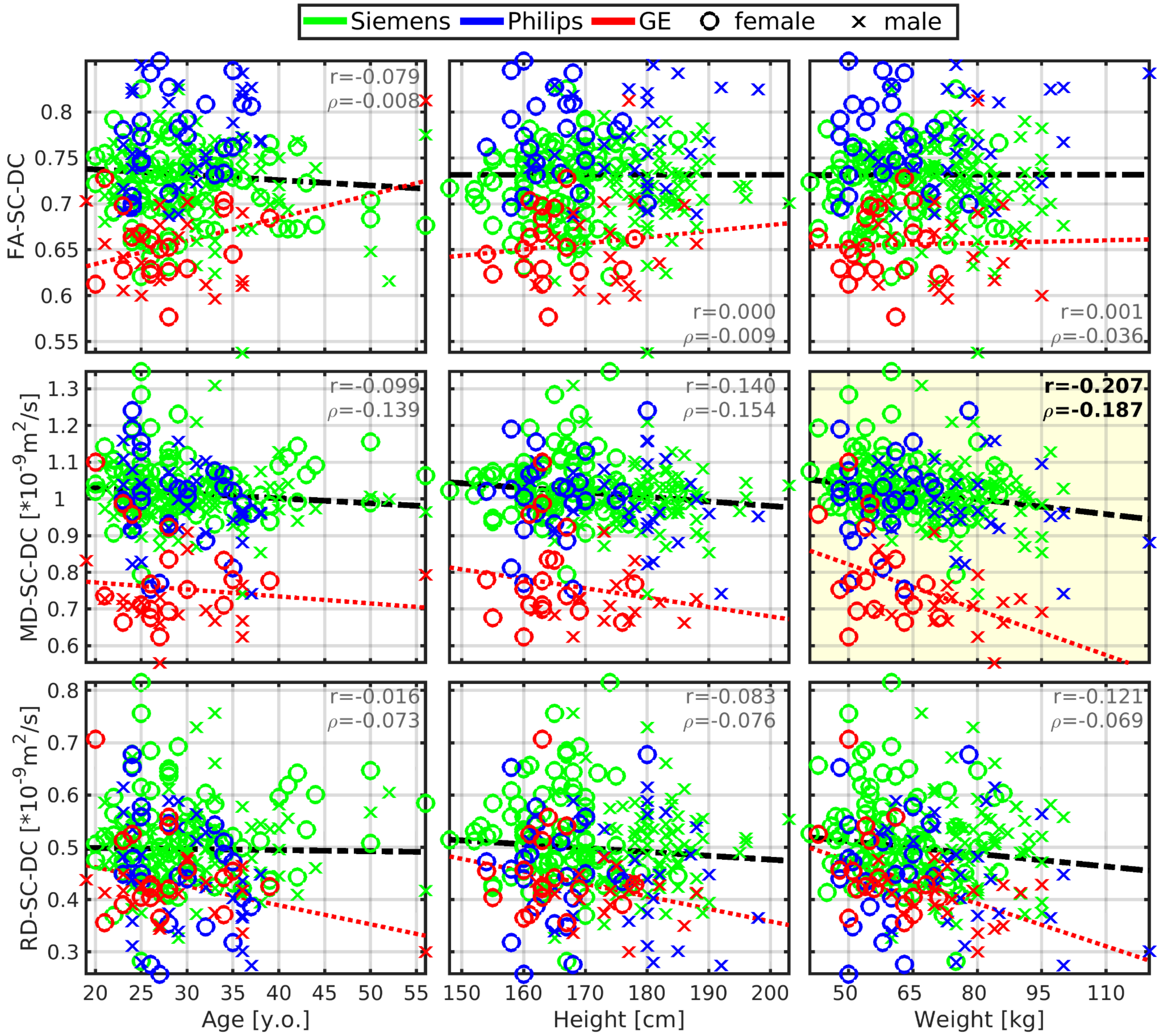

Figure S3: Mean diffusivity in spinal cord (SC) dorsal columns (DC) correlates with body weight.
Abbreviations: DC - dorsal columns; SC - spinal cord; FA - fractional anisotropy; MD - mean diffusivity; RD - radial diffusivity; r - Pearson correlation coefficient; ρ - Spearman correlation coefficient. All spinal cord measurements were averaged from cervical C2-5 levels. Black dashed regression lines were estimated from the Siemens and Philips scanners' data points. Red dotted regression lines were estimated from the GE scanner's data points. Plots with statistically significant correlation (p_{FWE}<0.05) are highlighted with yellow background, and corresponding r and ρ values are highlighted with black bold font.

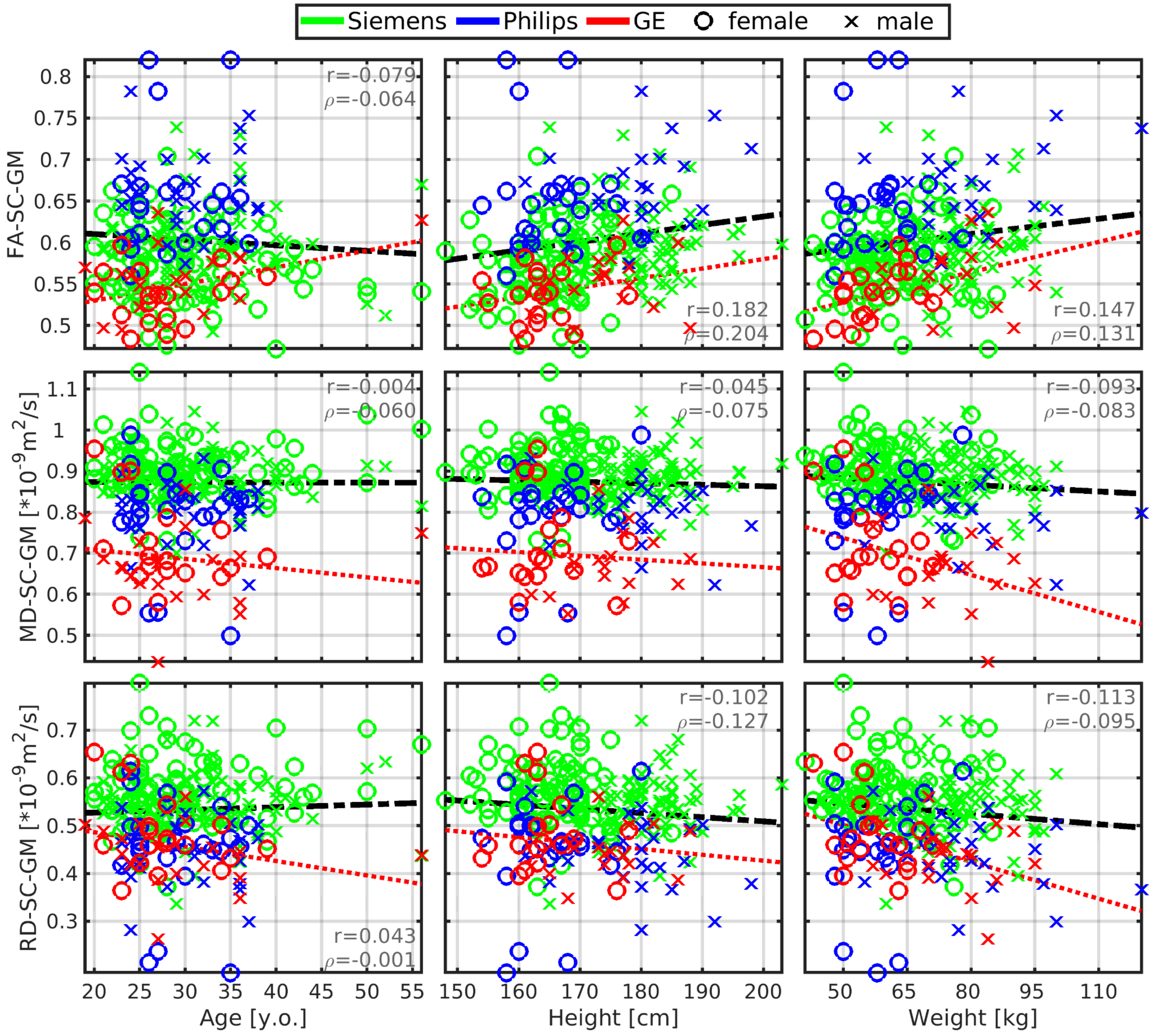

Figure S4: No correlation between diffusion tensor imaging in spinal cord (SC) gray matter (GM) and body size or age, respectively.

Abbreviations: GM - gray matter; SC - spinal cord; FA - fractional anisotropy; MD - mean diffusivity; RD - radial diffusivity; r - Pearson correlation coefficient; ρ - Spearman correlation coefficient. All spinal cord measurements were averaged from cervical C2-5 levels. Black dashed regression lines were estimated from the Siemens and Philips scanners' data points. Red dotted regression lines were estimated from the GE scanner's data points. No plotted correlations are significant (p_{FWE}<0.05).

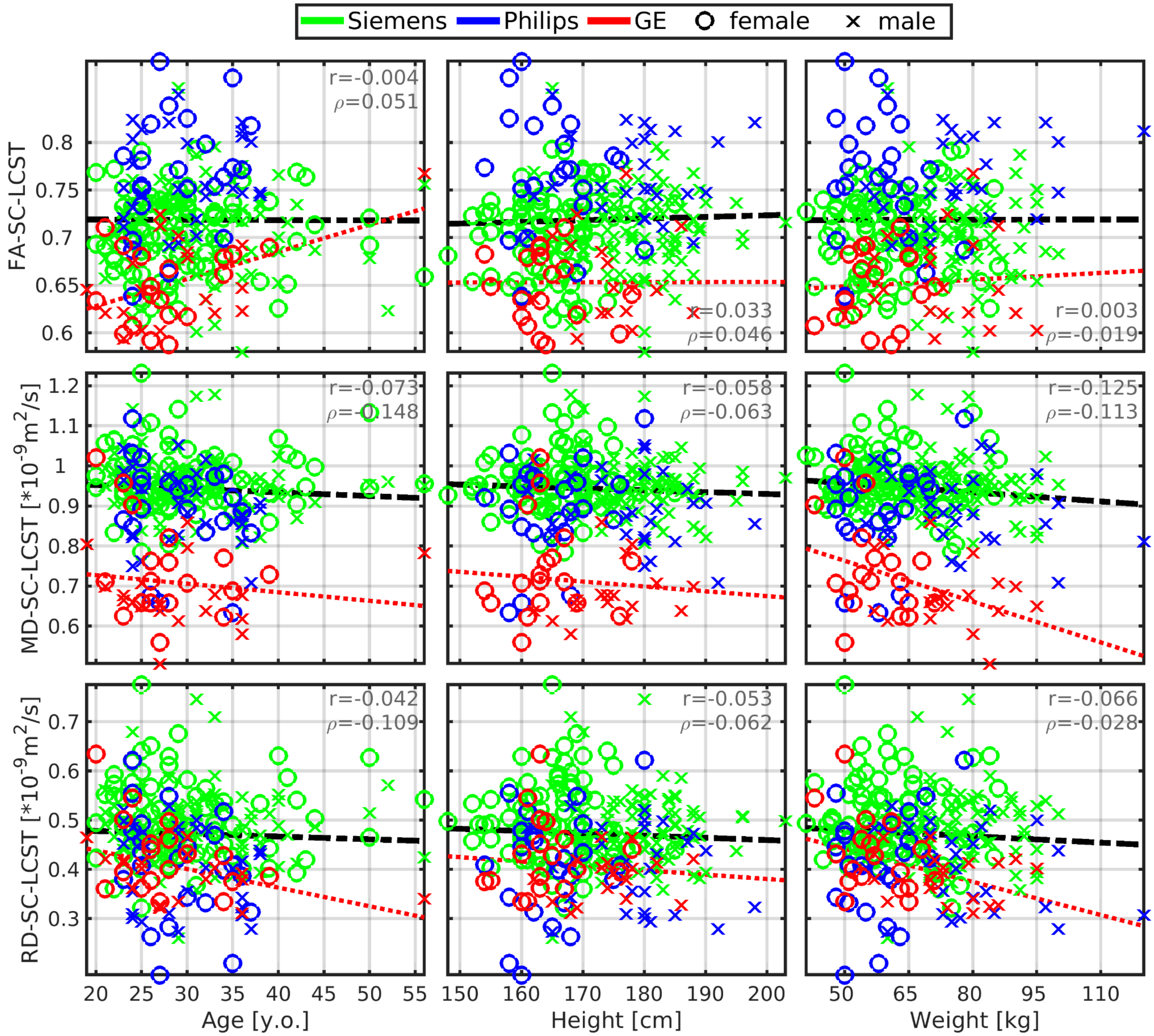

Figure S5: No correlation between diffusion tensor imaging in spinal cord lateral corticospinal tracts (LCST) and body size or age, respectively.

Abbreviations: LCST - lateral corticospinal tracts; SC - spinal cord; FA - fractional anisotropy; MD - mean diffusivity; RD - radial diffusivity; r - Pearson correlation coefficient; ρ - Spearman correlation coefficient. All spinal cord measurements were averaged from cervical C2-5 levels. Black dashed regression lines were estimated from the Siemens and Philips scanners' data points. Red dotted regression lines were estimated from the GE scanner's data points. No plotted correlations are significant (p_{FWE}<0.05).

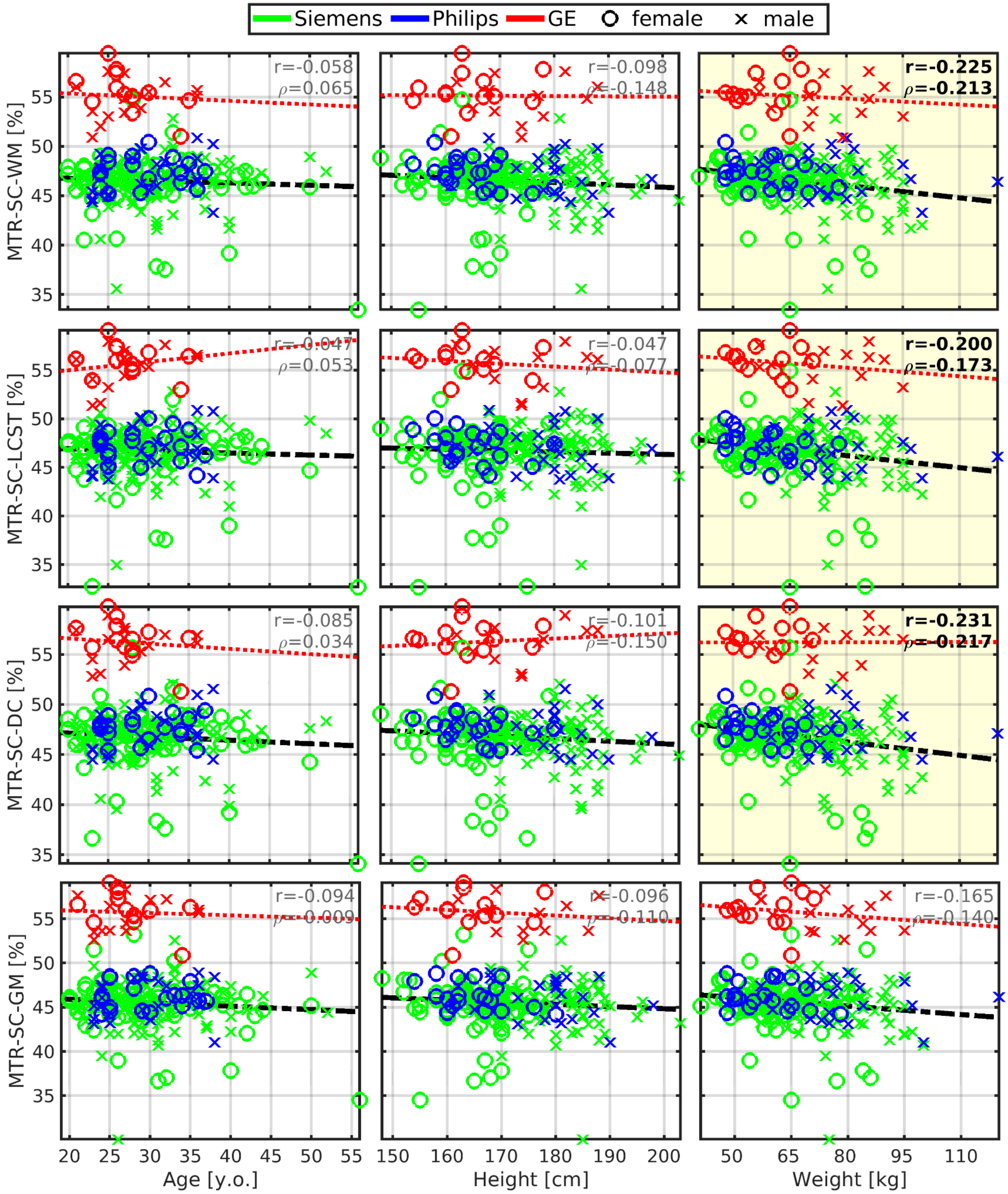

Figure S6: Spinal cord magnetization transfer ratio imaging correlates with body weight.

Abbreviations: WM - white matter; SC - spinal cord; LCST - lateral corticospinal tracts; DC - dorsal columns; GM - gray matter; MTR - magnetization transfer ratio; r - Pearson correlation coefficient; ρ - Spearman correlation coefficient. All spinal cord measurements were averaged from cervical C2-5 levels. Black dashed regression lines were estimated from the Siemens and Philips scanners' data points. Red dotted regression lines were estimated from the GE scanner's data points. Plots with statistically significant correlation ($p_{FWE}<0.05$) are highlighted with yellow background, and corresponding r and ρ values are highlighted with black bold font.

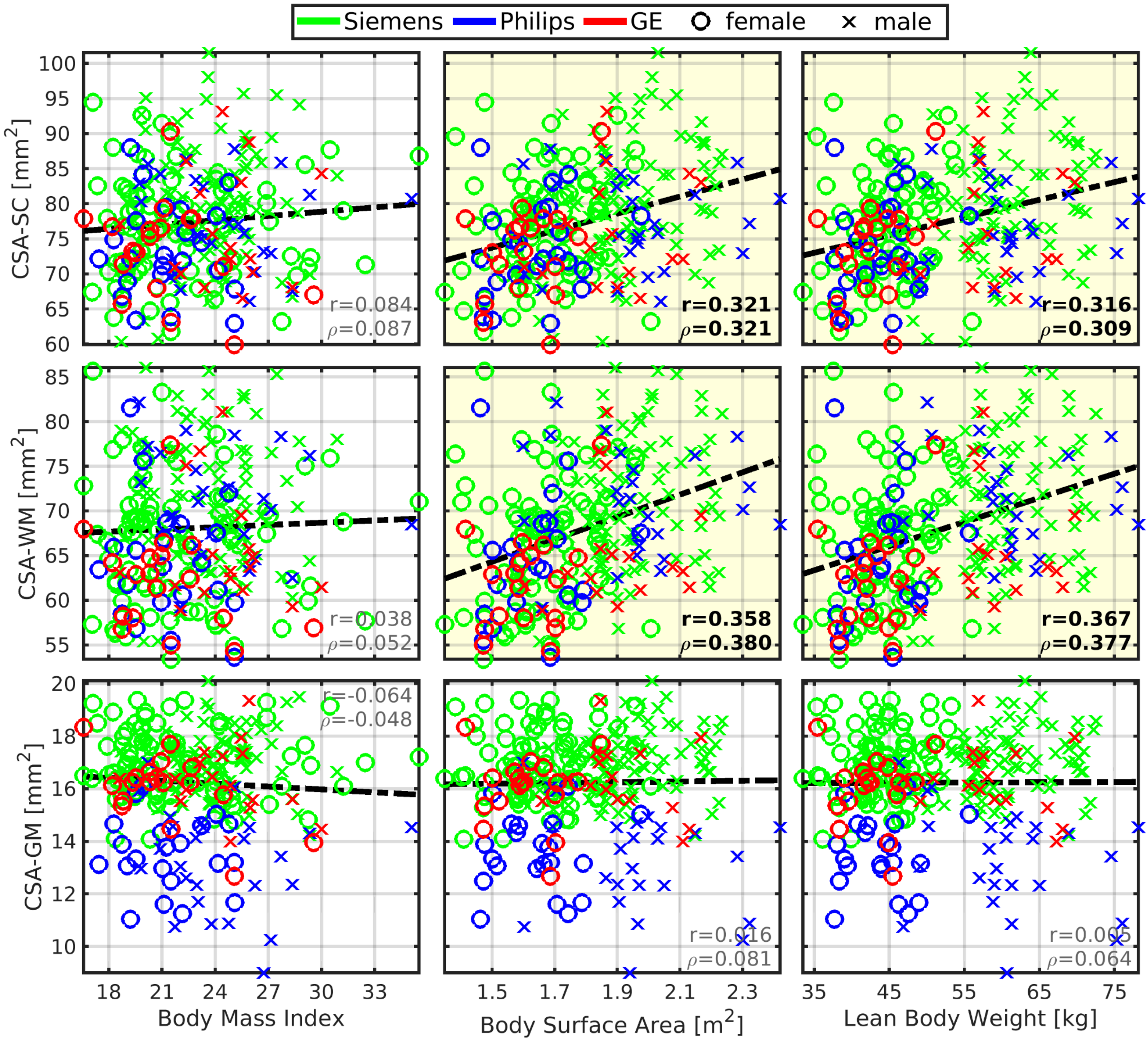

Figure S7: Cross-sectional area of spinal cord white matter correlates with body surface area and lean body weight.

Abbreviations: CSA - cross-sectional area; SC - spinal cord; WM - white matter; GM - gray matter; r - Pearson correlation coefficient; ρ - Spearman correlation coefficient. All spinal cord measurements were averaged from cervical C3-4 levels. Regression lines (i.e., the dashed black lines) were estimated from all available data points. Plots with statistically significant correlation (p_{FWE}<0.05) are highlighted with yellow background, and corresponding r and ρ values are highlighted with black bold font.

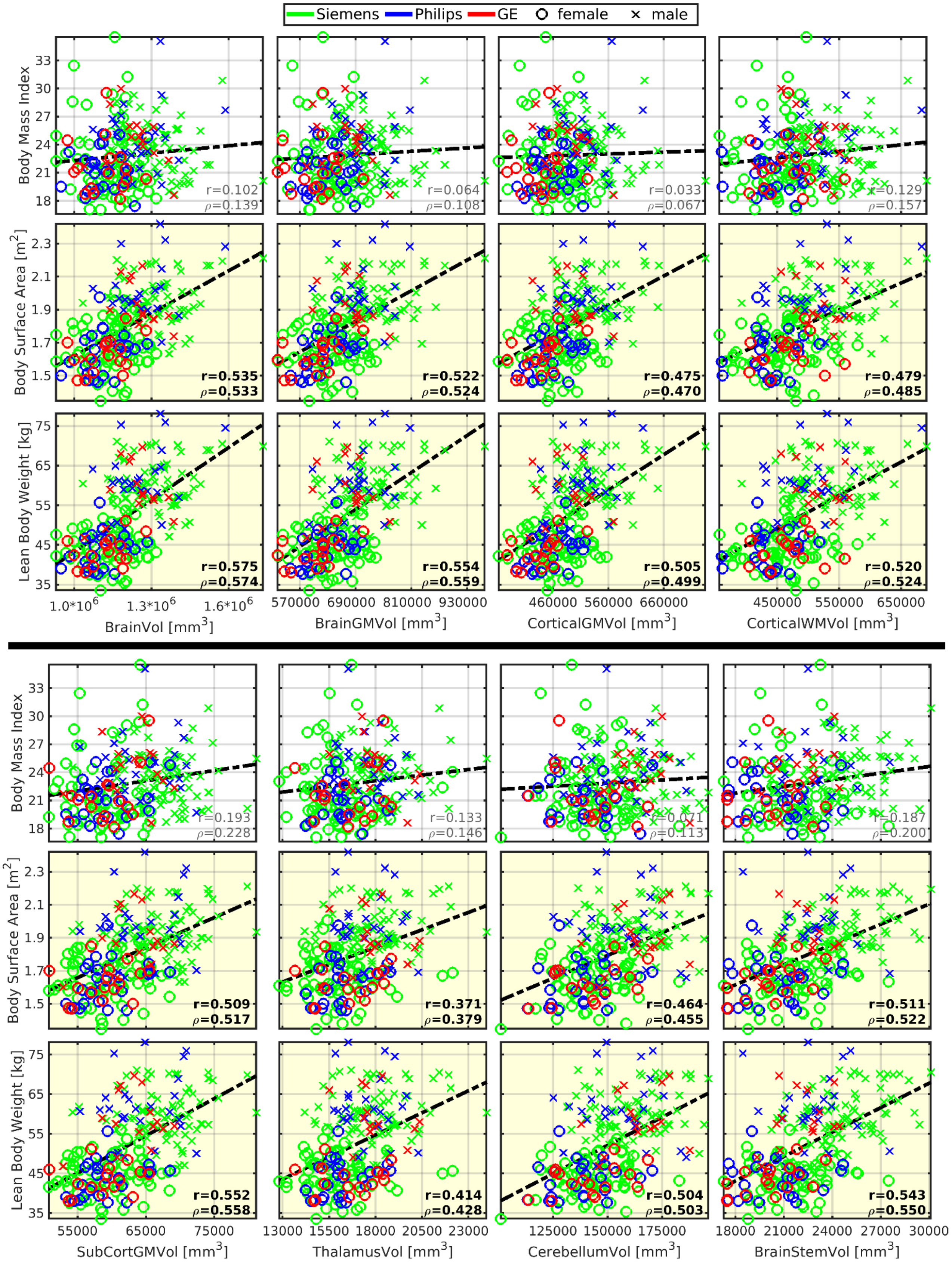

Figure S8: Brain morphology correlates with body surface area and lean body weight.

Abbreviations: WM - white matter; GM - gray matter; Vol - volume; SubCort - subcortical; r - Pearson correlation coefficient; ρ - Spearman correlation coefficient. Regression lines (i.e., the dashed black lines) were estimated from all available data points. Plots with statistically significant correlation ($p_{FWE}<0.05$) are highlighted with yellow background, and corresponding r and ρ values are highlighted with black bold font.

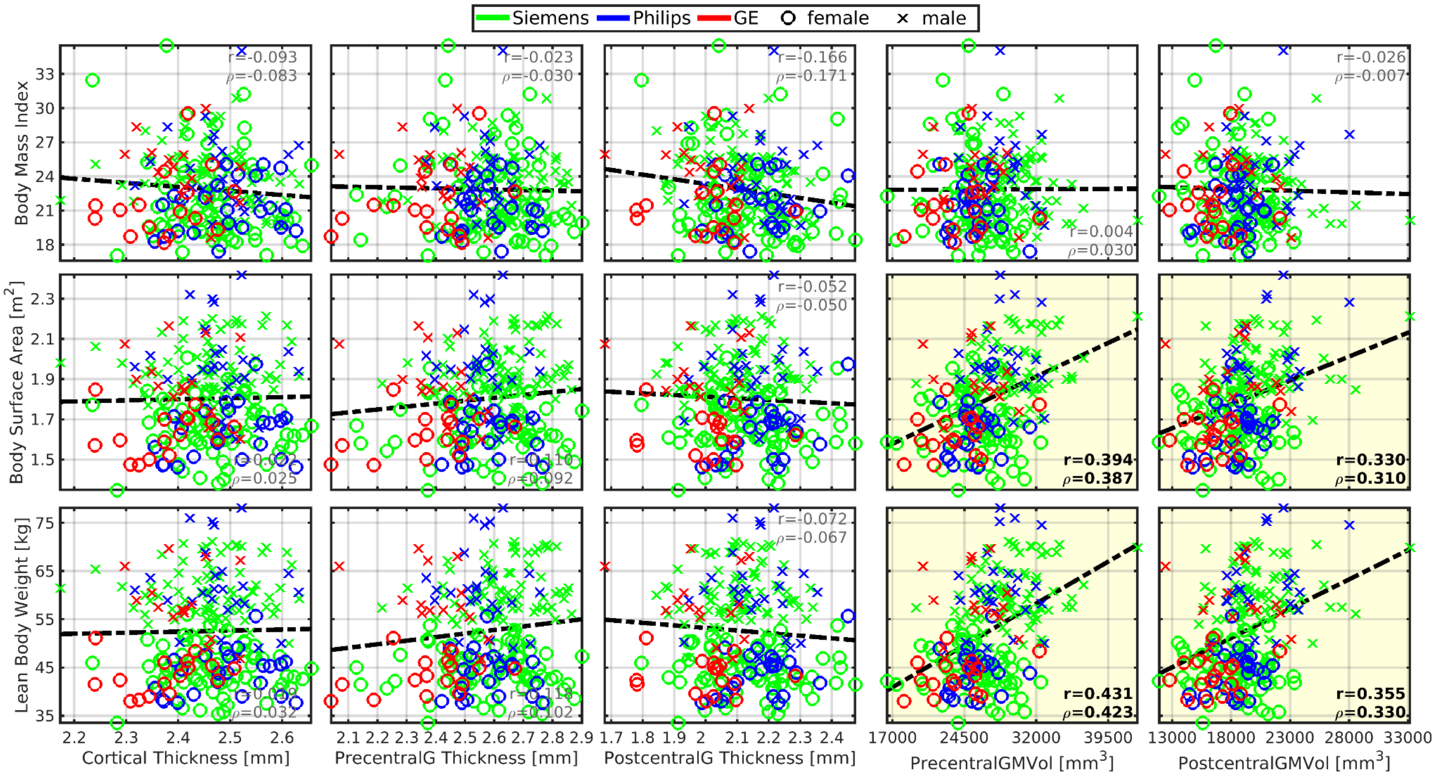

Figure S9: Precentral and postcentral gray matter volumes correlate with body surface area and lean body weight.

Abbreviations: WM - white matter; GM - gray matter; PrecentralG - precentral gyrus; PostcentralG - postcentral gyrus; Vol - volume; r - Pearson correlation coefficient; ρ - Spearman correlation coefficient. Regression lines (i.e., the dashed black lines) were estimated from all available data points. Plots with statistically significant correlation ($p_{FWE}<0.05$) are highlighted with yellow background, and corresponding r and ρ values are highlighted with black bold font.

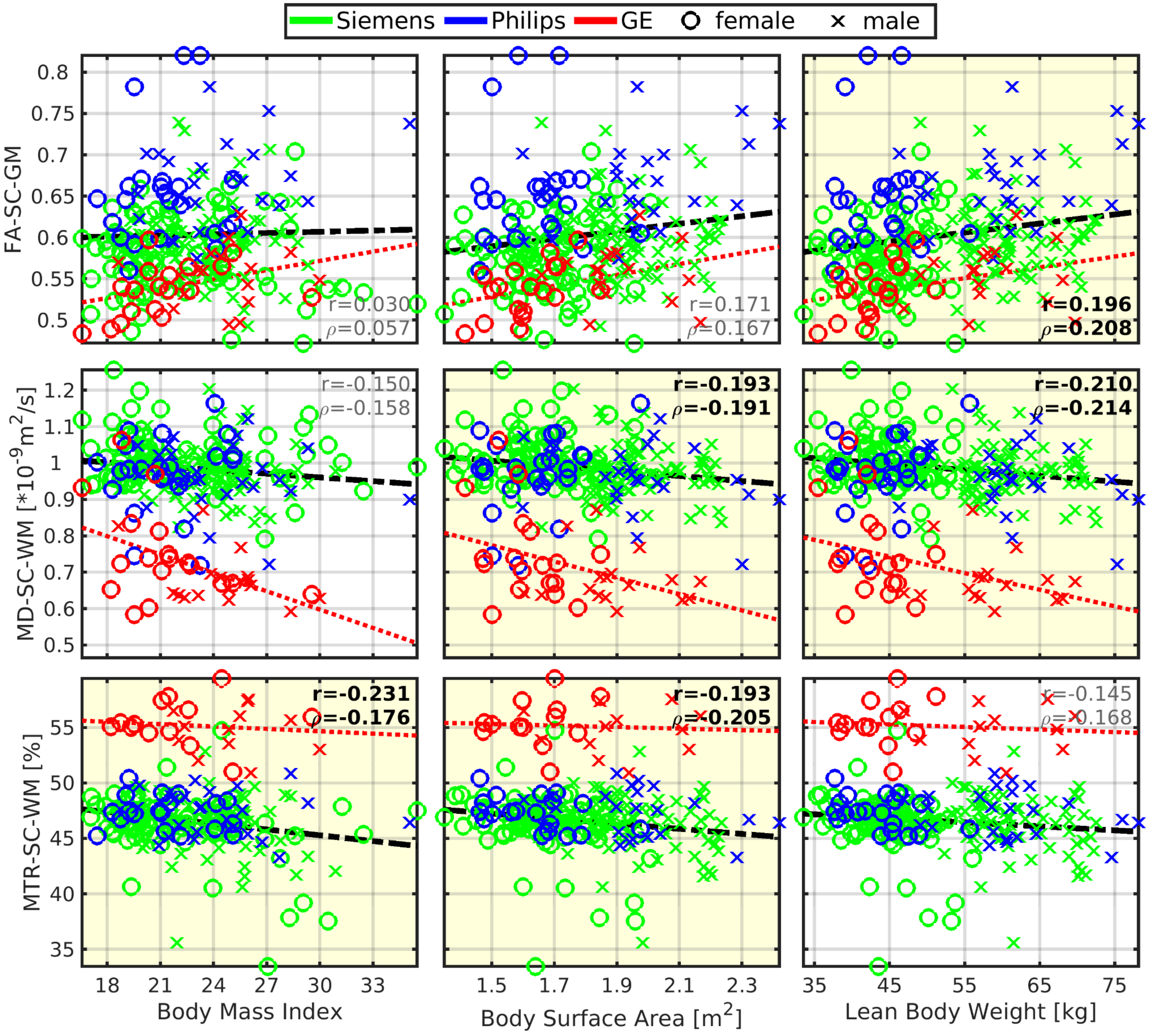

Figure S10: Mean diffusivity and magnetization transfer ratio in spinal cord white matter correlate with body mass index, body surface area or lean body weight.

<u>*Abbreviations:*</u> GM - gray matter; WM - white matter; SC - spinal cord; FA - fractional anisotropy; MD - mean diffusivity; MTR - magnetization transfer ratio; r - Pearson correlation coefficient; ρ - Spearman correlation coefficient. All spinal cord measurements were averaged from cervical C2-5 levels. Black dashed regression lines were estimated from the Siemens and Philips scanners' data points. Red dotted regression lines were estimated from the GE scanner's data points. Plots with statistically significant correlation ($p_{FWE}<0.05$) are highlighted with yellow background, and corresponding r and ρ values are highlighted with black bold font.

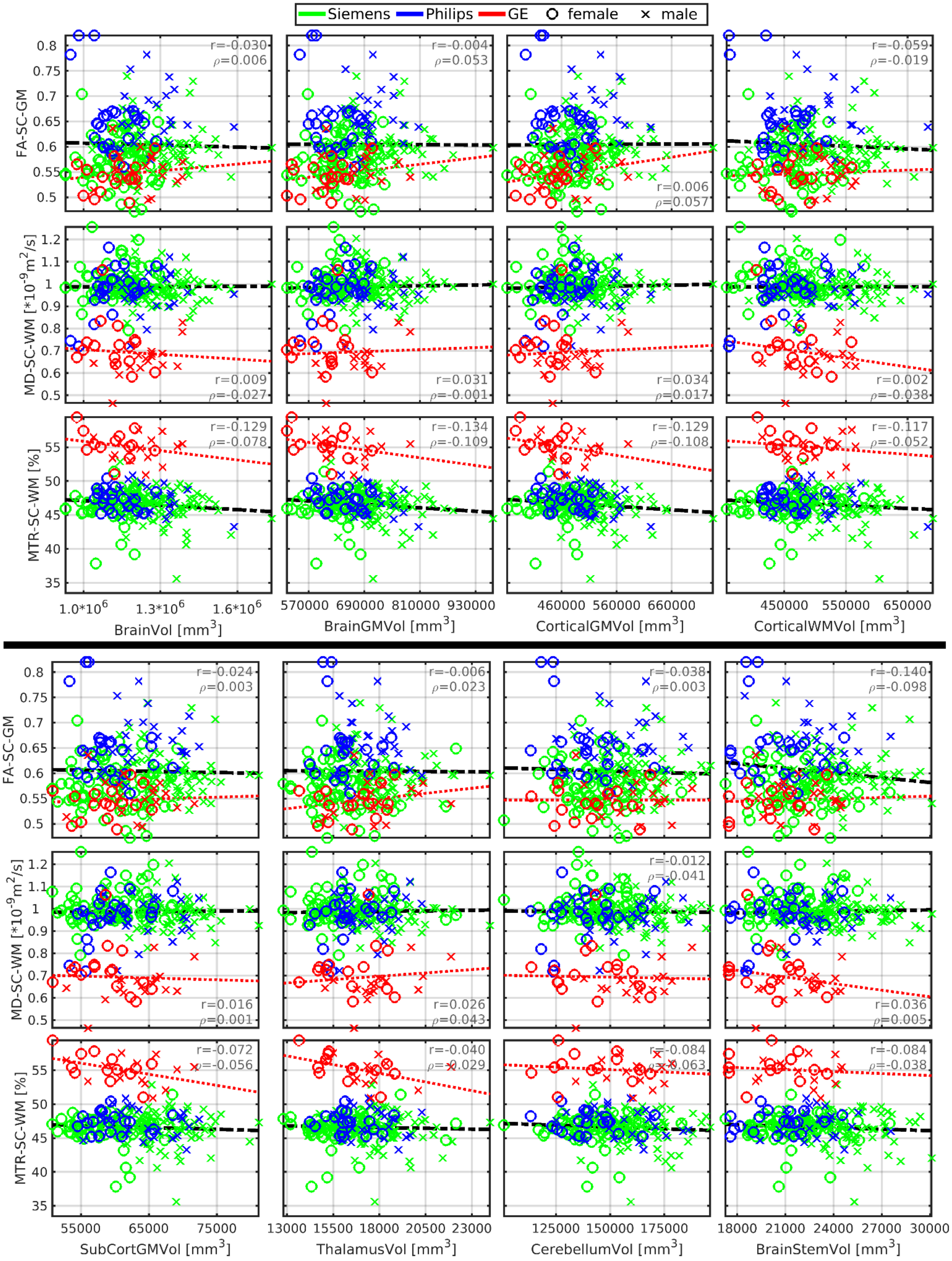

Figure S11: No links between cerebral macrostructure (x-axis) and spinal cord microstructure (y-axis). *Abbreviations:* WM - white matter; SC - spinal cord; FA - fractional anisotropy; MD - mean diffusivity; MTR - magnetization transfer ratio; GM - gray matter; Vol - volume; SubCort - subcortical; r - Pearson correlation coefficient; ρ - Spearman correlation coefficient. Regression lines (i.e., the dashed black lines) were estimated from all available data points. No plotted correlations are significant ($p_{FWE}<0.05$).

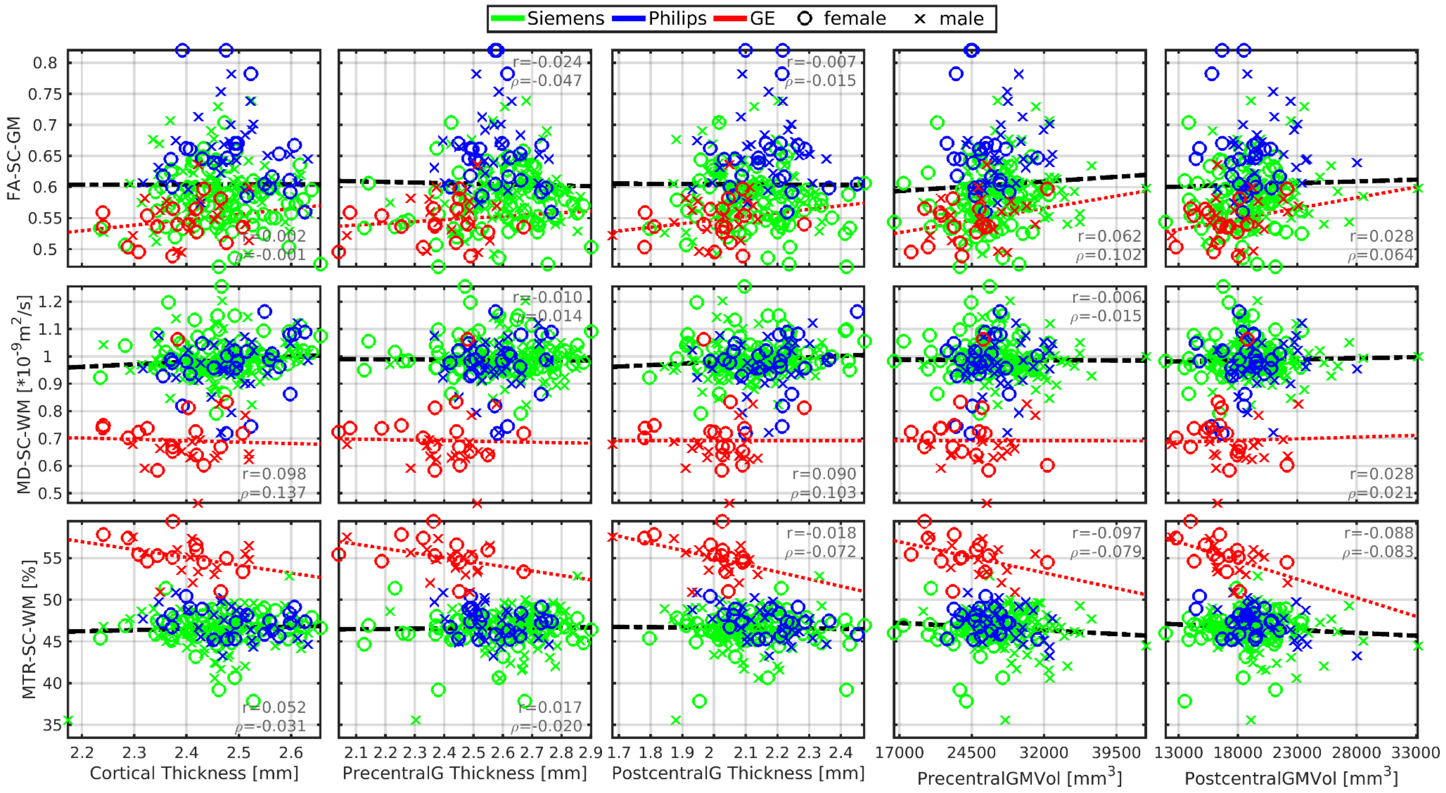

Figure S12: No links between cortical macrostructure (x-axis) and spinal cord microstructure (y-axis). <u>*Abbreviations:*</u> WM - white matter; SC - spinal cord; FA - fractional anisotropy; MD - mean diffusivity; MTR - magnetization transfer ratio; GM - gray matter; Vol - volume; r - Pearson correlation coefficient; ρ - Spearman correlation coefficient. Regression lines (i.e., the dashed black lines) were estimated from all available data points. No plotted correlations are significant (p_{FWE}<0.05).

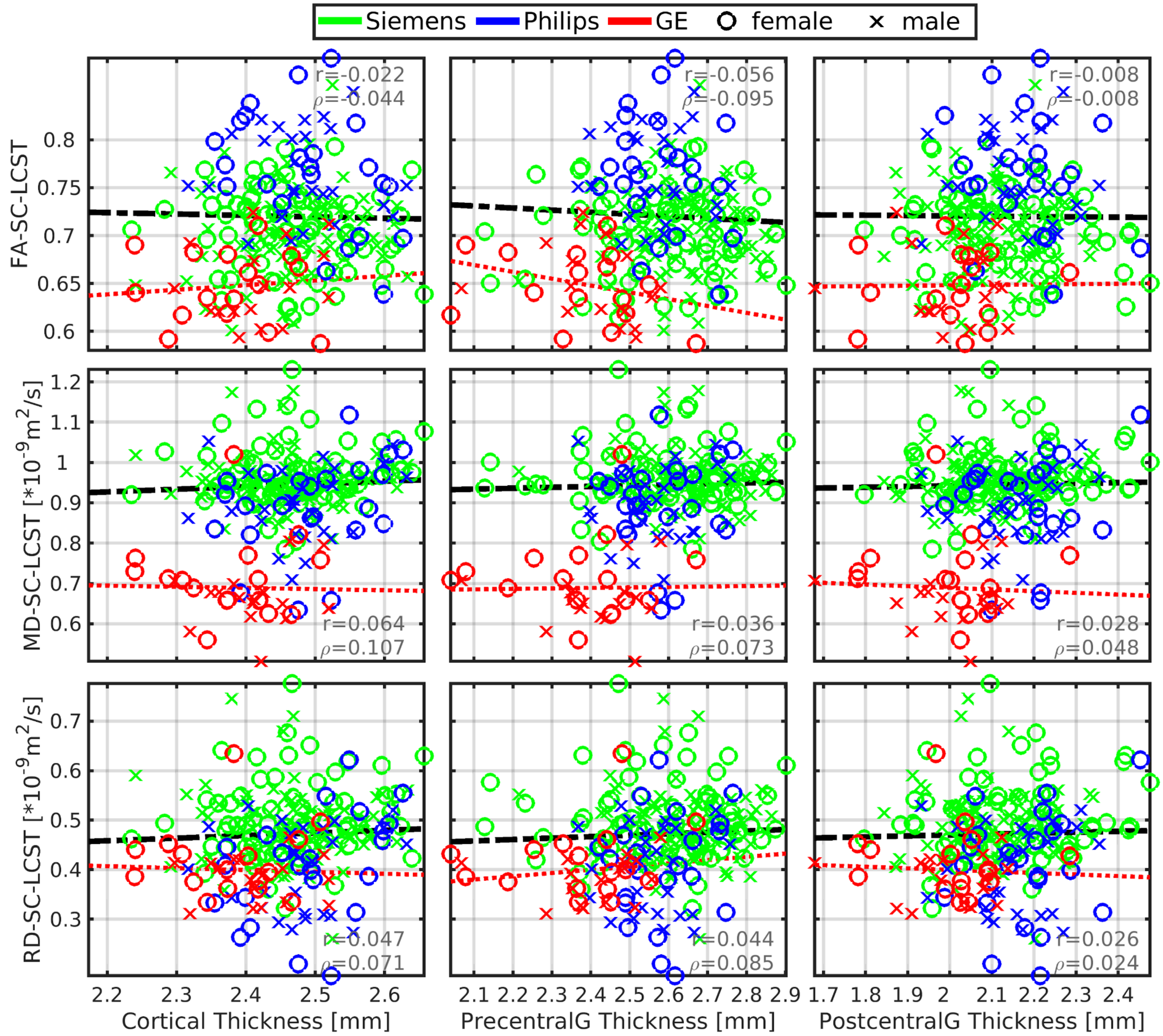

Figure S13: No links between cortical thicknesses (x-axis) and spinal cord diffusion tensor imaging of bilateral lateral corticospinal tracts (y-axis).

Abbreviations: LCST - lateral corticospinal tracts; SC - spinal cord; FA - fractional anisotropy; MD - mean diffusivity; RD - radial diffusivity; r - Pearson correlation coefficient; ρ - Spearman correlation coefficient. All spinal cord measurements were averaged from cervical C2-5 levels. Black dashed regression lines were estimated from the Siemens and Philips scanners' data points. Red dotted regression lines were estimated from the GE scanner's data points.No plotted correlations are significant ($p_{FWE}<0.05$).

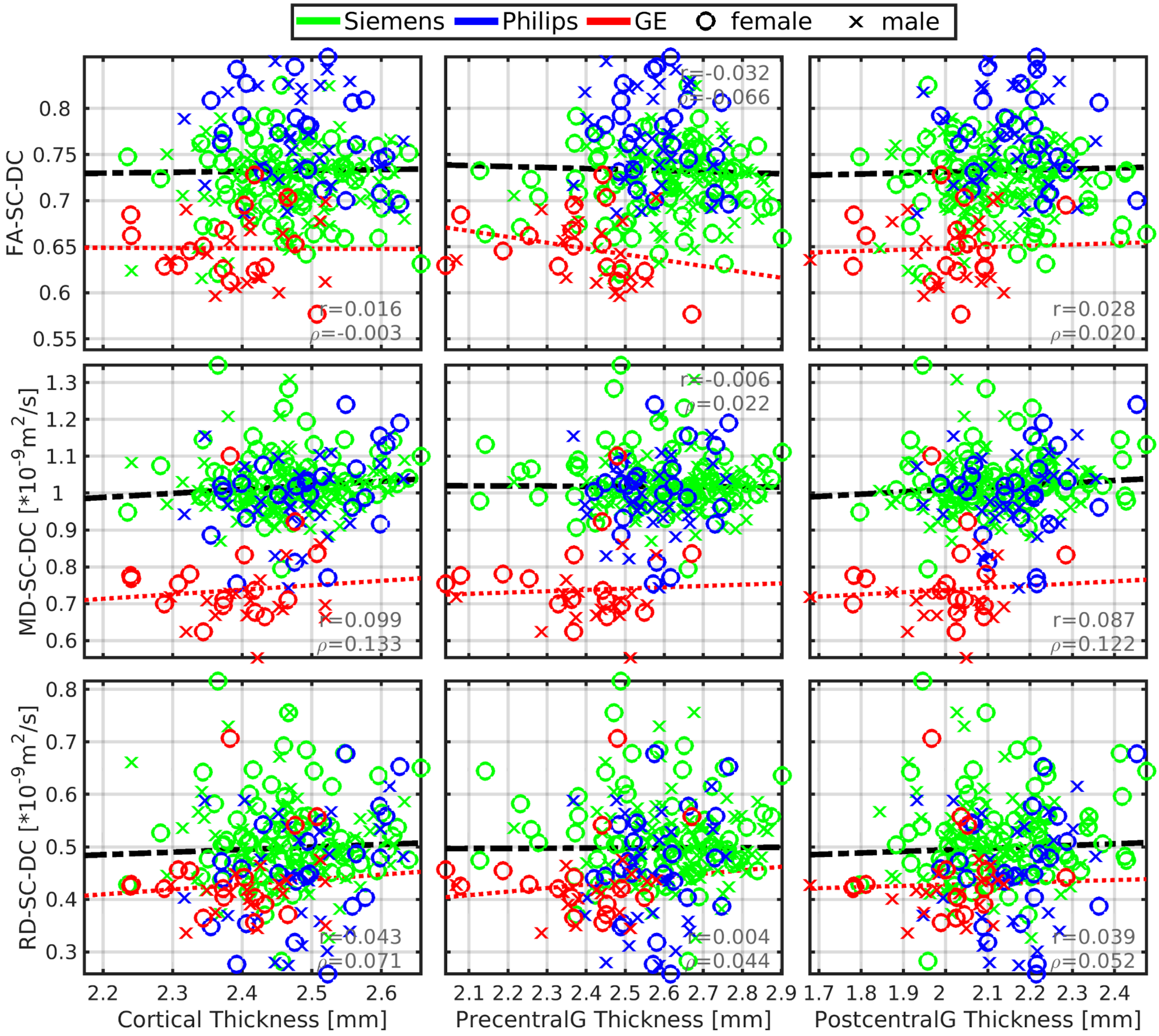

Figure S14: No links between cortical thicknesses (x-axis) and spinal cord diffusion tensor imaging of bilateral dorsal columns (y-axis).

Abbreviations: DC - dorsal columns; SC - spinal cord; FA - fractional anisotropy; MD - mean diffusivity; RD - radial diffusivity; r - Pearson correlation coefficient; ρ - Spearman correlation coefficient. All spinal cord measurements were averaged from cervical C2-5 levels. Black dashed regression lines were estimated from the Siemens and Philips scanners' data points. Red dotted regression lines were estimated from the GE scanner's data points. No plotted correlations are significant (p_{FWE}<0.05).

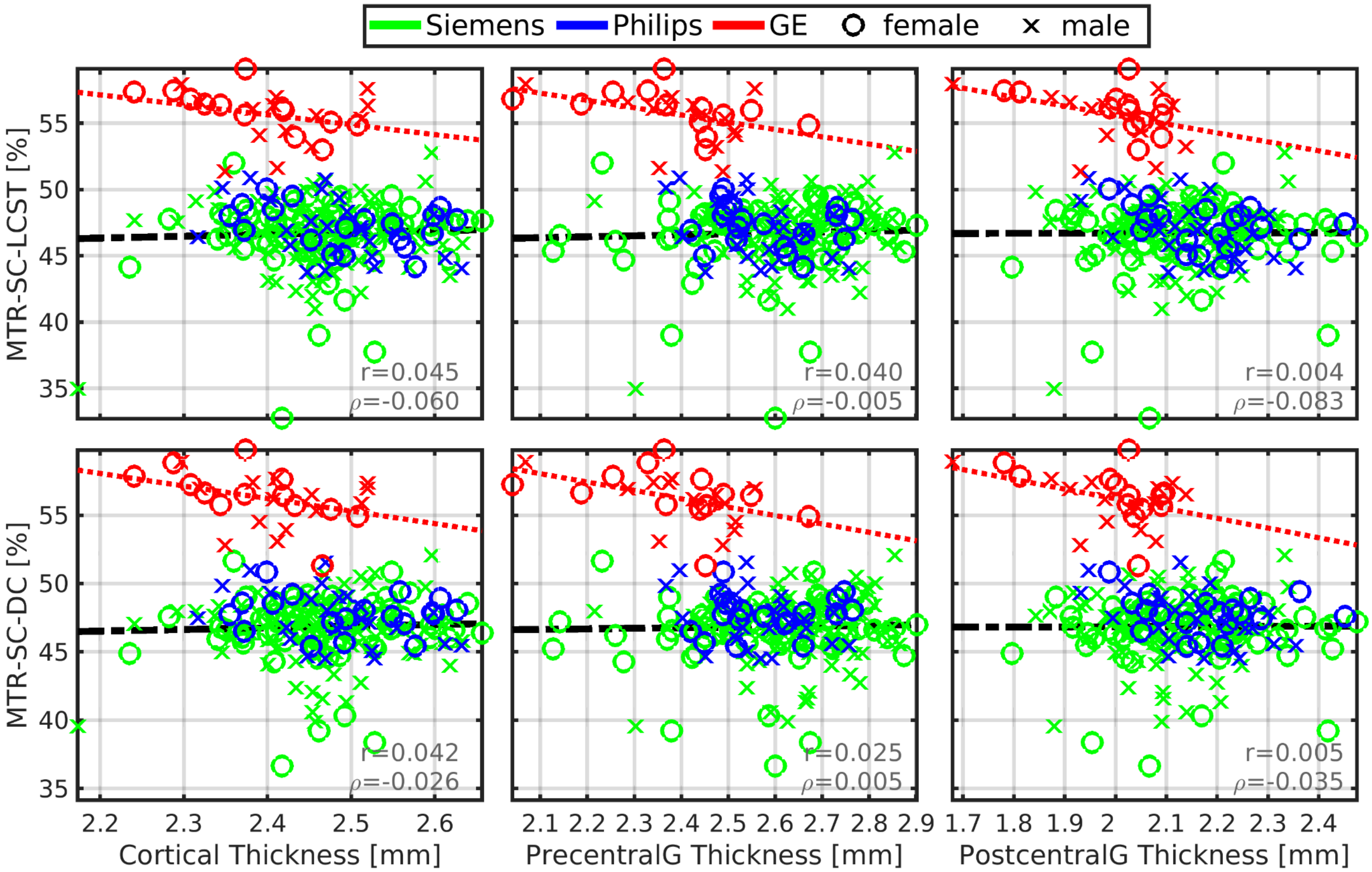

Figure S15: No links between cortical thicknesses (x-axis) and magnetization transfer ratio of bilateral lateral corticospinal tracts and bilateral dorsal columns (y-axis).

Abbreviations: SC - spinal cord; LCST - lateral corticospinal tracts; DC - dorsal columns; MTR - magnetization transfer ratio; r - Pearson correlation coefficient; ρ - Spearman correlation coefficient. All spinal cord measurements were averaged from cervical C2-5 levels. Black dashed regression lines were estimated from the Siemens and Philips scanners' data points. Red dotted regression lines were estimated from the GE scanner's data points. No plotted correlations are significant (p_{FWE}<0.05).

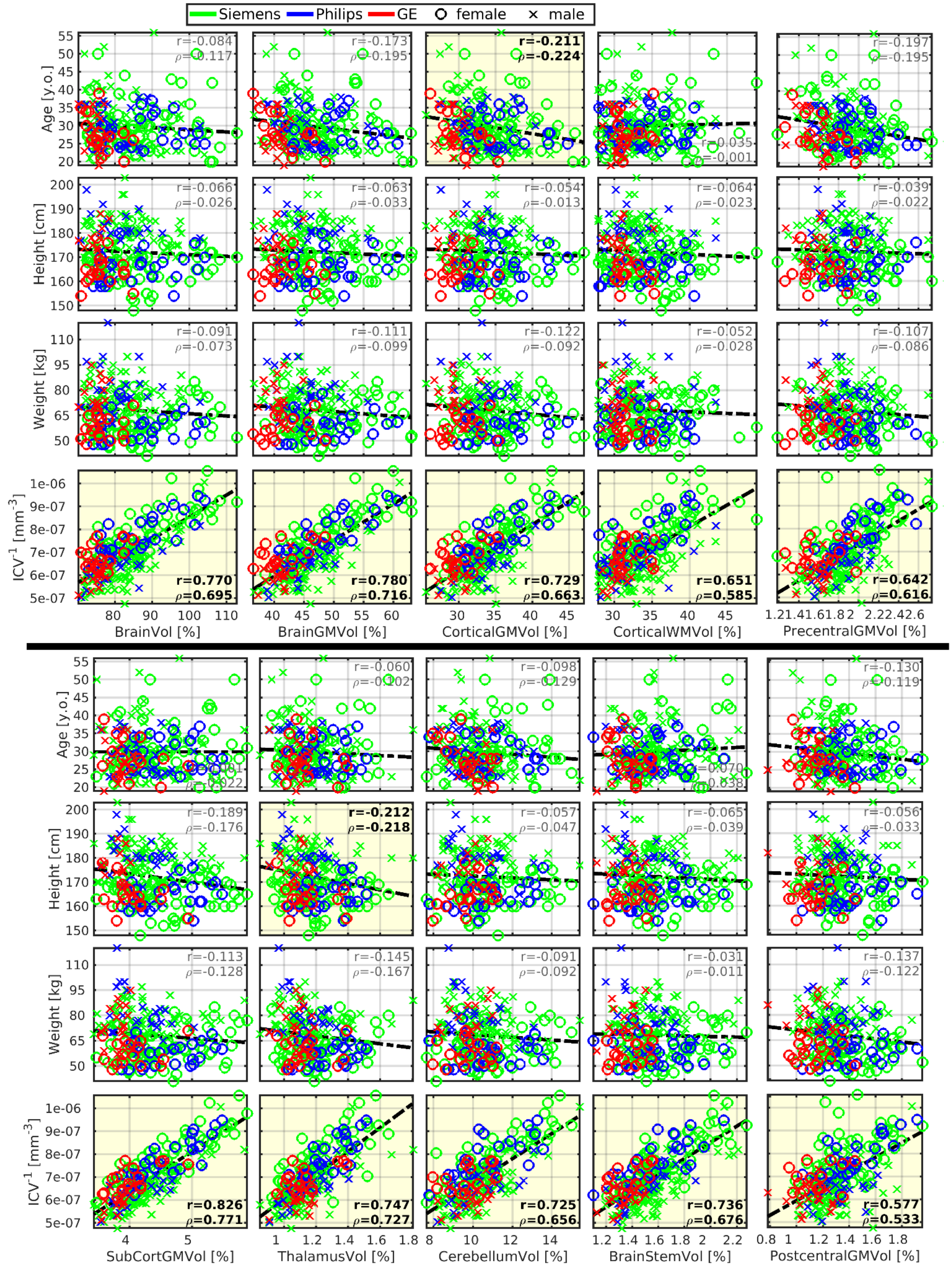

Figure S16: ICV-normalized brain morphology correlates with ICV^{-1} but weakly with age.

Abbreviations: ICV - intracranial volume; WM - white matter; GM - gray matter; Vol - volume; SubCort - subcortical; r - Pearson correlation coefficient; ρ - Spearman correlation coefficient. Regression lines (i.e., the dashed black lines) were estimated from all available data points. Plots with statistically significant correlation (p_{FWE}<0.05) are highlighted with yellow background, and corresponding r and ρ values are highlighted with black bold font.

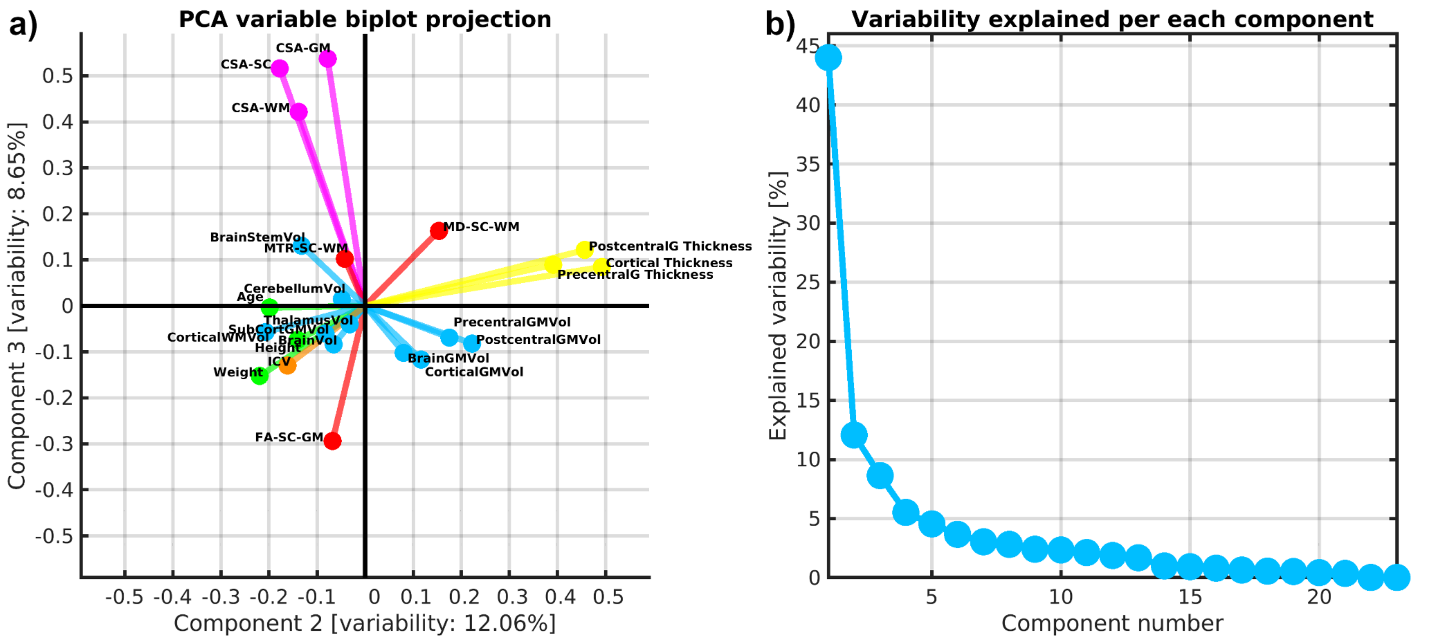

Figure S17: Biplot projection of 2nd and 3rd principal components (a) and explained data variability per principal component (b).

Supplementary Table 1: Sex-specific impact of manual segmentation edits on brain morphology measurements. The p-values are p-values of two-sample t-test between non-edited and manually edited FreeSurfer based segmentations. The p-values $p<0.05$ are highlighted with black bold font. Neither mean error difference is higher than 5%. *Abbreviations:* STD - standard deviation; WM - white matter; GM - gray matter; Vol - volume; SubCort - subcortical; PrecentralG - precentral gyrus; PostcentralG - postcentral gyrus.

			BrainVol [mm^3]	BrainGMVol [mm^3]	CorticalGMVol [mm^3]	CorticalWMVol [mm^3]	SubCortGMVol [mm^3]	ThalamusVol [mm^3]	CerebellumVol [mm^3]	BrainStemVol [mm^3]	Cortical Thickness [mm]	PrecentralG Thickness [mm]	PostcentralG Thickness [mm]	PrecentralGMVol [mm^3]	PostcentralGMVol [mm^3]
**MALE**		p-value	0.0818	**0.0182**	**0.0061**	0.3417	0.7109	0.0790	0.3629	0.9850	0.5998	0.9894	0.8076	**0.0203**	0.0709
	non-edited	Mean	1264540	695110	502793	516693	65893	17577	159161	23872	2.46	2.59	2.10	27852	19636
		STD	110317	56009	46459	58374	5187	1593	13569	2276	0.08	0.14	0.12	3000	2561
		Min	1033199	584482	416188	392250	52872	14168	125144	18460	2.24	2.07	1.68	19441	12399
		Max	1586324	832376	621823	682879	81152	23982	192054	29082	2.61	2.85	2.33	33242	28014
	edited	Mean	1300342	722093	528418	526218	66231	18098	161619	23864	2.47	2.59	2.10	29263	20562
		STD	119649	69706	56225	54830	5091	1723	16492	2427	0.08	0.14	0.12	3698	3118
		Min	1084884	590176	439499	426081	55191	14700	129748	19145	2.17	2.30	1.84	22307	15237
		Max	1733505	967485	735407	691227	79818	22033	196123	30117	2.63	2.86	2.35	42583	33180
**FEMALE**		p-value	0.1406	**0.0056**	**0.0028**	0.7477	0.4457	0.1186	0.3965	0.4884	**0.0349**	0.4245	0.0584	0.1050	0.0704
	non-edited	Mean	1122239	621282	451009	457516	59117	15963	140990	21035	2.44	2.54	2.11	24961	17770
		STD	79286	45638	37200	38014	4224	1342	14496	2011	0.08	0.16	0.12	2860	2422
		Min	928679	522290	361338	356792	50799	13190	101120	17478	2.24	2.04	1.78	16425	11898
		Max	1317982	705414	515040	549141	68453	19144	182798	27478	2.63	2.84	2.42	31328	23280
	edited	Mean	1147626	647654	474544	454712	59761	16447	143333	21315	2.48	2.57	2.16	25917	18614
		STD	98642	50401	42087	52982	4374	1874	13221	2118	0.10	0.19	0.17	3169	2237
		Min	948607	550133	394927	360258	52124	12812	118216	17257	2.24	2.08	1.78	17423	13838
		Max	1294419	733312	556335	579359	69169	22137	170509	25573	2.66	2.90	2.48	32467	23376

Supplementary Table 2: Full table of Pearson correlation coefficients (r) between body size, age, spinal cord structure, and brain structure, and post-hoc sex-effects in the correlation analysis.

Abbreviations: CSA - cross-sectional area; SC - spinal cord; WM - white matter; GM - gray matter; Vol - volume.

The correlation p-values (p) are uncorrected. Table 2 in the main paper is a subset of this supplementary table. CSA was measured as averages between C3-C4 segments. DTI and MTR were calculated as averages between C2-C5 segments. The column denoted “Original values” reports correlation coefficients for raw measurements with no normalization procedure prior to the correlation analysis. The column denoted “Manufacturer-specific normalized SC measures” reports correlation coefficients for SC structural measurements, which were normalized to zero mean for each scanner manufacturer before correlation analysis. Empty cells in the right half of the table represent combinations where no updated correlation coefficients were measured, because the utilized normalization of SC structural measurements had no effect on these correlation coefficients. Brain structural measurements were not considered necessary to normalize as we did not observe strong scanner-related effects in brain macrostructural measurements.

	Original values						Manufacturer-specific normalized values					
	All		Female		Male		All		Female		Male	
Correlation pair	r	p	r	p	r	p	r	p	r	p	r	p
Age vs CSA-SC	0.047	4.5E-1	0.031	7.3E-1	0.038	6.6E-1	0.029	6.5E-1	0.004	9.7E-1	0.024	7.8E-1
Age vs CSA-WM	-0.001	9.8E-1	-0.015	8.7E-1	-0.043	6.2E-1	-0.026	6.7E-1	-0.049	5.9E-1	-0.064	4.5E-1
Age vs CSA-GM	0.038	5.4E-1	-0.003	9.7E-1	0.070	4.1E-1	-0.001	9.9E-1	-0.053	5.6E-1	0.039	6.5E-1
Height vs CSA-SC	0.355	5.0E-9	0.323	2.9E-4	0.230	7.3E-3	0.344	1.5E-8	0.319	3.5E-4	0.205	1.7E-2
Height vs CSA-WM	0.437	1.7E-13	0.295	9.9E-4	0.303	3.2E-4	0.422	1.4E-12	0.285	1.4E-3	0.268	1.5E-3
Height vs CSA-GM	0.070	2.6E-1	0.148	1.0E-1	0.052	5.4E-1	0.092	1.4E-1	0.213	1.9E-2	0.026	7.7E-1
Weight vs CSA-SC	0.261	2.2E-5	0.140	1.2E-1	0.154	7.3E-2	0.256	3.1E-5	0.108	2.4E-1	0.159	6.3E-2
Weight vs CSA-WM	0.274	7.3E-6	0.100	2.7E-1	0.084	3.2E-1	0.266	1.4E-5	0.071	4.3E-1	0.077	3.7E-1
Weight vs CSA-GM	-0.021	7.3E-1	0.056	5.4E-1	-0.089	3.0E-1	-0.020	7.5E-1	0.002	9.8E-1	-0.075	3.8E-1
Age vs FA-WM	-0.033	6.2E-1	0.027	7.8E-1	-0.108	2.4E-1	0.043	4.9E-1	0.057	5.3E-1	0.011	9.0E-1
Age vs MD-WM	-0.085	2.0E-1	-0.024	8.1E-1	-0.124	1.8E-1	-0.093	1.3E-1	-0.079	3.8E-1	-0.077	3.7E-1
Age vs RD-WM	-0.030	6.5E-1	-0.022	8.2E-1	-0.014	8.8E-1	-0.080	2.0E-1	-0.070	4.4E-1	-0.060	4.8E-1
Height vs FA-WM	0.111	9.8E-2	0.064	5.1E-1	0.040	6.6E-1	0.132	3.4E-2	0.060	5.1E-1	0.068	4.3E-1
Height vs MD-WM	-0.136	4.1E-2	0.126	2.0E-1	-0.125	1.8E-1	-0.147	1.8E-2	0.082	3.7E-1	-0.088	3.1E-1
Height vs RD-WM	-0.133	4.7E-2	0.041	6.8E-1	-0.074	4.3E-1	-0.156	1.2E-2	0.022	8.1E-1	-0.073	4.0E-1
Weight vs FA-WM	0.074	2.7E-1	-0.013	9.0E-1	0.031	7.4E-1	0.103	9.8E-2	0.054	5.5E-1	0.018	8.3E-1
Weight vs MD-WM	-0.200	2.6E-3	-0.022	8.2E-1	-0.191	3.8E-2	-0.252	3.8E-5	-0.108	2.3E-1	-0.206	1.6E-2
Weight vs RD-WM	-0.145	3.0E-2	0.007	9.4E-1	-0.108	2.4E-1	-0.203	9.6E-4	-0.080	3.8E-1	-0.124	1.5E-1
Age vs MTR-WM	-0.058	4.0E-1	-0.233	2.0E-2	0.125	1.9E-1	-0.053	4.2E-1	-0.255	6.6E-3	0.157	8.0E-2
Height vs MTR-WM	-0.098	1.6E-1	-0.090	3.8E-1	-0.189	4.6E-2	-0.091	1.6E-1	-0.065	5.0E-1	-0.151	9.4E-2
Weight vs MTR-WM	-0.225	1.0E-3	-0.374	1.4E-4	-0.221	1.9E-2	-0.221	6.1E-4	-0.331	3.6E-4	-0.217	1.5E-2
BrainVol vs CSA-SC	0.417	3.6E-11	0.337	3.7E-4	0.361	3.9E-5	0.391	6.5E-10	0.311	1.0E-3	0.321	2.7E-4
BrainVol vs CSA-WM	0.519	1.7E-17	0.392	2.9E-5	0.445	1.8E-7	0.503	2.3E-16	0.379	5.7E-5	0.413	1.5E-6
BrainVol vs CSA-GM	0.221	6.9E-4	0.250	9.4E-3	0.296	7.6E-4	0.186	4.3E-3	0.219	2.3E-2	0.255	4.0E-3
BrainGMVol vs CSA-SC	0.357	2.3E-8	0.265	5.6E-3	0.283	1.4E-3	0.324	4.6E-7	0.225	1.9E-2	0.239	7.6E-3
BrainGMVol vs CSA-WM	0.479	9.5E-15	0.341	3.3E-4	0.397	4.1E-6	0.443	1.3E-12	0.286	2.8E-3	0.351	5.5E-5
BrainGMVol vs CSA-GM	0.160	1.5E-2	0.147	1.3E-1	0.227	1.1E-2	0.128	5.1E-2	0.133	1.7E-1	0.182	4.1E-2
CorticalGMVol vs CSA-SC	0.319	6.7E-7	0.195	4.3E-2	0.259	3.7E-3	0.291	6.8E-6	0.159	1.0E-1	0.223	1.3E-2
CorticalGMVol vs CSA-WM	0.447	7.5E-13	0.283	3.2E-3	0.383	9.8E-6	0.411	6.8E-11	0.227	1.9E-2	0.340	9.8E-5
CorticalGMVol vs CSA-GM	0.124	5.9E-2	0.102	3.0E-1	0.176	4.8E-2	0.106	1.1E-1	0.093	3.4E-1	0.154	8.5E-2
CorticalWMVol vs CSA-SC	0.430	7.8E-12	0.291	2.3E-3	0.414	1.8E-6	0.409	9.4E-11	0.280	3.3E-3	0.377	1.6E-5
CorticalWMVol vs CSA-WM	0.498	5.7E-16	0.315	9.5E-4	0.456	8.3E-8	0.496	7.1E-16	0.339	3.6E-4	0.432	4.3E-7
CorticalWMVol vs CSA-GM	0.267	3.5E-5	0.275	4.1E-3	0.352	5.3E-5	0.218	8.2E-4	0.215	2.6E-2	0.302	6.0E-4
SubCortGMVol vs CSA-SC	0.415	4.5E-11	0.207	3.2E-2	0.441	3.0E-7	0.387	1.1E-9	0.198	4.0E-2	0.385	1.0E-5
SubCortGMVol vs CSA-WM	0.506	1.5E-16	0.257	7.5E-3	0.508	1.2E-9	0.483	4.9E-15	0.248	1.0E-2	0.459	6.6E-8
SubCortGMVol vs CSA-GM	0.216	8.9E-4	0.093	3.4E-1	0.389	6.7E-6	0.197	2.5E-3	0.119	2.2E-1	0.344	8.0E-5
ThalamusVol vs CSA-SC	0.329	2.8E-7	0.176	6.8E-2	0.308	5.1E-4	0.333	2.0E-7	0.201	3.7E-2	0.296	8.6E-4
ThalamusVol vs CSA-WM	0.421	2.0E-11	0.244	1.1E-2	0.373	1.7E-5	0.441	1.6E-12	0.279	3.6E-3	0.384	8.9E-6
ThalamusVol vs CSA-GM	0.165	1.2E-2	0.058	5.5E-1	0.292	9.2E-4	0.212	1.1E-3	0.159	1.0E-1	0.316	3.1E-4
CerebellumVol vs CSA-SC	0.355	2.6E-8	0.508	2.0E-8	0.104	2.5E-1	0.341	1.0E-7	0.493	6.0E-8	0.082	3.6E-1
CerebellumVol vs CSA-WM	0.430	6.5E-12	0.501	3.8E-8	0.170	5.7E-2	0.438	2.3E-12	0.508	2.4E-8	0.177	4.7E-2
CerebellumVol vs CSA-GM	0.199	2.2E-3	0.325	6.4E-4	0.181	4.2E-2	0.187	4.2E-3	0.374	7.3E-5	0.122	1.7E-1
BrainStemVol vs CSA-SC	0.572	1.4E-21	0.622	6.5E-13	0.485	1.2E-8	0.517	2.9E-17	0.573	9.1E-11	0.403	3.5E-6
BrainStemVol vs CSA-WM	0.635	1.2E-27	0.624	6.8E-13	0.533	1.3E-10	0.585	8.0E-23	0.580	6.0E-11	0.454	9.2E-8
BrainStemVol vs CSA-GM	0.390	6.8E-10	0.444	1.6E-6	0.478	1.6E-8	0.335	1.7E-7	0.440	2.1E-6	0.376	1.5E-5
PrecentralGMVol vs CSA-SC	0.307	1.9E-6	0.159	1.0E-1	0.273	2.1E-3	0.275	2.2E-5	0.132	1.7E-1	0.228	1.1E-2
PrecentralGMVol vs CSA-WM	0.420	2.2E-11	0.235	1.5E-2	0.382	9.9E-6	0.370	5.7E-9	0.178	6.7E-2	0.317	2.9E-4
PrecentralGMVol vs CSA-GM	0.119	6.9E-2	0.080	4.1E-1	0.176	4.8E-2	0.102	1.2E-1	0.092	3.5E-1	0.141	1.2E-1

PostcentralGMVol vs CSA-SC	0.240	**2.3E-4**	0.146	1.3E-1	0.184	**4.1E-2**	0.218	**8.1E-4**	0.120	2.2E-1	0.160	7.5E-2
PostcentralGMVol vs CSA-WM	0.389	**8.0E-10**	0.243	**1.2E-2**	0.356	**4.2E-5**	0.345	**6.6E-8**	0.181	6.1E-2	0.311	**3.9E-4**
PostcentralGMVol vs CSA-GM	0.034	6.1E-1	0.008	9.3E-1	0.059	5.1E-1	0.064	3.3E-1	0.050	6.1E-1	0.088	3.2E-1
Cortical Thickness vs CSA-SC	0.067	3.1E-1	0.077	4.3E-1	0.049	5.9E-1	0.044	5.0E-1	0.046	6.3E-1	0.031	7.3E-1
Cortical Thickness vs CSA-WM	0.154	**1.8E-2**	0.184	5.7E-2	0.133	1.4E-1	0.093	1.6E-1	0.096	3.3E-1	0.089	3.2E-1
Cortical Thickness vs CSA-GM	-0.069	2.9E-1	-0.051	6.0E-1	-0.085	3.4E-1	0.029	6.6E-1	0.090	3.5E-1	-0.028	7.6E-1
PrecentralG Thickness vs CSA-SC	0.211	**1.3E-3**	0.180	6.3E-2	0.201	**2.5E-2**	0.144	**2.8E-2**	0.125	2.0E-1	0.117	2.0E-1
PrecentralG Thickness vs CSA-WM	0.252	**9.9E-5**	0.249	**9.7E-3**	0.209	**1.9E-2**	0.146	**2.5E-2**	0.142	1.5E-1	0.087	3.3E-1
PrecentralG Thickness vs CSA-GM	0.113	8.5E-2	0.083	4.0E-1	0.149	9.6E-2	0.081	2.2E-1	0.122	2.1E-1	0.044	6.2E-1
PostcentralG Thickness vs CSA-SC	0.035	6.0E-1	0.145	1.3E-1	-0.013	8.8E-1	0.014	8.3E-1	0.116	2.3E-1	-0.027	7.6E-1
PostcentralG Thickness vs CSA-WM	0.152	**2.1E-2**	0.245	**1.1E-2**	0.171	5.6E-2	0.084	2.0E-1	0.165	9.0E-2	0.108	2.3E-1
PostcentralG Thickness vs CSA-GM	-0.069	2.9E-1	0.018	8.5E-1	-0.160	7.4E-2	0.062	3.4E-1	0.152	1.2E-1	-0.030	7.4E-1
BrainVol vs Age	-0.079	2.3E-1	-0.165	8.4E-2	-0.148	9.5E-2						
BrainVol vs Height	0.611	**1.1E-25**	0.274	**3.8E-3**	0.409	**1.8E-6**						
BrainVol vs Weight	0.422	**1.0E-11**	0.057	5.6E-1	0.119	1.8E-1						
BrainGMVol vs Age	-0.188	**3.6E-3**	-0.309	**1.0E-3**	-0.263	**2.6E-3**						
BrainGMVol vs Height	0.622	**8.7E-27**	0.321	**6.3E-4**	0.446	**1.5E-7**						
BrainGMVol vs Weight	0.394	**2.6E-10**	0.101	2.9E-1	0.071	4.2E-1						
CorticalGMVol vs Age	-0.213	**9.4E-4**	-0.357	**1.3E-4**	-0.257	**3.3E-3**						
CorticalGMVol vs Height	0.583	**5.5E-23**	0.252	**7.9E-3**	0.449	**1.2E-7**						
CorticalGMVol vs Weight	0.351	**2.4E-8**	0.041	6.7E-1	0.070	4.3E-1						
CorticalWMVol vs Age	0.034	6.0E-1	0.005	9.6E-1	-0.017	8.5E-1						
CorticalWMVol vs Height	0.522	**5.8E-18**	0.157	1.0E-1	0.313	**3.3E-4**						
CorticalWMVol vs Weight	0.395	**2.5E-10**	0.023	8.1E-1	0.142	1.1E-1						
SubCortGMVol vs Age	0.006	9.3E-1	-0.003	9.8E-1	-0.076	3.9E-1						
SubCortGMVol vs Height	0.521	**6.6E-18**	0.107	2.6E-1	0.288	**1.0E-3**						
SubCortGMVol vs Weight	0.425	**6.3E-12**	0.169	7.8E-2	0.081	3.6E-1						
ThalamusVol vs Age	-0.084	2.0E-1	-0.012	9.0E-1	-0.226	**9.9E-3**						
ThalamusVol vs Height	0.385	**8.9E-10**	0.031	7.5E-1	0.173	5.2E-2						
ThalamusVol vs Weight	0.301	**2.1E-6**	0.041	6.7E-1	0.015	8.7E-1						
CerebellumVol vs Age	-0.120	6.5E-2	-0.097	3.1E-1	-0.258	**3.1E-3**						
CerebellumVol vs Height	0.546	**8.7E-20**	0.411	**8.3E-6**	0.219	**1.3E-2**						
CerebellumVol vs Weight	0.354	**1.9E-8**	0.095	3.2E-1	0.005	9.6E-1						
BrainStemVol vs Age	0.094	1.5E-1	0.076	4.3E-1	0.055	5.4E-1						
BrainStemVol vs Height	0.530	**1.4E-18**	0.310	**9.8E-4**	0.259	**3.3E-3**						
BrainStemVol vs Weight	0.431	**3.3E-12**	0.119	2.1E-1	0.193	**2.9E-2**						
PrecentralGMVol vs Age	-0.205	**1.4E-3**	-0.326	**5.2E-4**	-0.232	**8.3E-3**						
PrecentralGMVol vs Height	0.495	**4.9E-16**	0.092	3.4E-1	0.418	**1.0E-6**						
PrecentralGMVol vs Weight	0.289	**5.7E-6**	-0.114	2.4E-1	0.102	2.5E-1						
PostcentralGMVol vs Age	-0.118	6.8E-2	-0.174	7.0E-2	-0.145	1.0E-1						
PostcentralGMVol vs Height	0.434	**2.8E-12**	0.121	2.1E-1	0.369	**2.0E-5**						
PostcentralGMVol vs Weight	0.231	**3.1E-4**	-0.089	3.6E-1	0.080	3.7E-1						
Cortical Thickness vs Age	-0.274	**1.8E-5**	-0.278	**3.3E-3**	-0.277	**1.5E-3**						
Cortical Thickness vs Height	0.089	1.7E-1	0.115	2.3E-1	0.091	3.1E-1						
Cortical Thickness vs Weight	-0.020	7.6E-1	0.010	9.1E-1	-0.083	3.5E-1						
PrecentralG Thickness vs Age	-0.169	**8.9E-3**	-0.245	**9.8E-3**	-0.119	1.8E-1						
PrecentralG Thickness vs Height	0.154	**1.8E-2**	0.082	4.0E-1	0.119	1.8E-1						
PrecentralG Thickness vs Weight	0.074	2.6E-1	-0.012	9.0E-1	0.019	8.3E-1						
PostcentralG Thickness vs Age	-0.149	**2.2E-2**	-0.097	3.1E-1	-0.187	**3.4E-2**						
PostcentralG Thickness vs Height	0.041	5.3E-1	0.146	1.3E-1	0.161	7.1E-2						
PostcentralG Thickness vs Weight	-0.094	1.5E-1	-0.041	6.7E-1	-0.032	7.1E-1						
ICV vs BrainVol	0.773	**7.9E-49**	0.522	**5.1E-9**	0.750	**1.5E-24**						
ICV vs BrainGMVol	0.703	**5.9E-37**	0.412	**7.6E-6**	0.663	**1.1E-17**						
ICV vs CorticalGMVol	0.684	**2.9E-34**	0.420	**5.1E-6**	0.644	**1.7E-16**						
ICV vs CorticalWMVol	0.723	**6.6E-40**	0.507	**1.6E-8**	0.666	**7.7E-18**						
ICV vs SubCortGMVol	0.673	**6.4E-33**	0.389	**2.6E-5**	0.604	**3.6E-14**						
ICV vs ThalamusVol	0.558	**5.4E-21**	0.372	**6.4E-5**	0.435	**2.5E-7**						
ICV vs CerebellumVol	0.556	**8.3E-21**	0.225	**1.8E-2**	0.470	**1.9E-8**						
ICV vs BrainStemVol	0.500	**1.7E-16**	0.145	1.3E-1	0.396	**3.4E-6**						
ICV vs PrecentralGMVol	0.524	**2.9E-18**	0.230	**1.6E-2**	0.461	**3.9E-8**						
ICV vs PostcentralGMVol	0.526	**2.2E-18**	0.290	**2.2E-3**	0.504	**1.2E-9**						
ICV vs CSA-SC	0.300	**3.2E-6**	0.108	2.7E-1	0.273	**2.2E-3**	0.312	**1.2E-6**	0.132	1.7E-1	0.279	**1.7E-3**
ICV vs CSA-WM	0.370	**5.5E-9**	0.140	1.5E-1	0.304	**5.3E-4**	0.413	**5.1E-11**	0.210	**3.0E-2**	0.342	**9.0E-5**
ICV vs MD-SC-WM	-0.015	8.3E-1	0.210	**4.2E-2**	-0.038	7.0E-1	-0.057	3.9E-1	0.116	2.3E-1	-0.014	8.8E-1
ICV vs MTR-SC-WM	-0.116	1.1E-1	0.003	9.8E-1	-0.205	**3.6E-2**	-0.122	7.2E-2	0.002	9.8E-1	-0.191	**3.8E-2**
ICV vs Cortical Thickness	0.042	5.2E-1	0.004	9.7E-1	0.064	4.7E-1						

Supplementary Table 3: Coefficients of determination (R^2) of regression models explaining a variable y as a linear mixture of age, sex, body height, body weight and/or intracranial volume.

Bold highlighted R^2 coefficients identifies models which explained most of the neuroimaging data variance by given demographic variables and/or intracranial volume.

Abbreviations: CSA - cross-sectional area; ICV - intracranial volume; SC - spinal cord; WM - white matter; GM - gray matter; Vol - volume; MD mean diffusivity; MTR - magnetization transfer ratio.

y	BrainGM Vol	Brain Vol	Cortical GMVol	CerebellumVol	BrainStemVol	Cortical WMVol	SubCort GMVol	PrecentralGMVol	PostcentralGMVol	CSA-WM	CSA-SC	MD-SC-WM	MTR-SC-WM	Cortical Thickness
y ∝ y0 + Age	3.5%	0.6%	4.5%	1.4%	0.9%	0.1%	0.0%	4.2%	1.4%	0.1%	0.1%	0.9%	0.3%	7.5%
y ∝ y0 + Sex	31.3%	34.8%	26.0%	28.7%	27.5%	29.1%	33.4%	20.4%	12.8%	11.7%	6.0%	4.6%	0.0%	0.0%
y ∝ y0 + Weight	15.5%	17.8%	12.3%	12.5%	18.5%	15.6%	18.1%	8.3%	5.3%	7.1%	6.5%	6.4%	4.9%	0.0%
y ∝ y0 + Height	38.7%	37.4%	34.0%	29.8%	28.1%	27.2%	27.2%	24.5%	18.8%	17.8%	11.8%	2.2%	0.8%	0.8%
y ∝ y0 + ICV	49.4%	59.8%	46.7%	30.9%	25.0%	52.2%	45.3%	27.5%	27.6%	17.1%	9.7%	0.3%	1.5%	0.2%
y ∝ y0 + Sex + Age	36.6%	36.3%	32.3%	31.3%	27.8%	29.1%	33.5%	26.2%	14.9%	12.0%	6.0%	5.1%	0.3%	7.7%
y ∝ y0 + Sex + Weight	31.7%	35.4%	26.2%	28.8%	29.4%	29.8%	34.2%	20.4%	12.8%	12.2%	7.8%	6.9%	7.1%	0.2%
y ∝ y0 + Sex + Height	42.6%	43.6%	36.7%	35.4%	33.8%	34.0%	37.2%	27.1%	19.8%	18.4%	11.9%	4.4%	1.2%	1.1%
y ∝ y0 + Sex + ICV	53.6%	63.6%	49.2%	38.5%	33.8%	55.0%	51.4%	31.2%	28.3%	19.1%	10.4%	3.4%	1.6%	0.2%
y ∝ y0 + Sex + Age + Weight	37.7%	37.3%	33.2%	31.7%	29.6%	29.8%	34.5%	26.5%	15.1%	12.6%	7.8%	**7.2%**	7.2%	7.7%
y ∝ y0 + Sex + Age + Height	46.3%	44.3%	41.3%	37.1%	34.5%	34.1%	37.2%	31.5%	21.1%	18.4%	12.1%	5.1%	1.8%	**8.1%**
y ∝ y0 + Sex + Age + ICV	58.1%	64.7%	54.7%	40.7%	34.2%	55.1%	51.5%	36.4%	30.0%	19.4%	10.5%	3.9%	1.8%	7.7%
y ∝ y0 + Sex + Age + ICV + Weight	58.9%	65.3%	55.2%	40.9%	35.8%	55.5%	52.2%	36.6%	30.0%	20.1%	12.4%	5.7%	**8.1%**	7.7%
y ∝ y0 + Sex + Age + ICV + Height	**63.9%**	**68.8%**	**59.7%**	**44.4%**	**39.2%**	**57.4%**	**53.1%**	**39.6%**	**33.9%**	**25.8%**	**16.2%**	3.8%	3.5%	**8.2%**

Supplementary Table 4: Stepwise linear regression fitted models predicting CNS structural measure (y) for males.
Abbreviations: ICV - intracranial volume; CSA - cross-sectional area; SC - spinal cord; WM - white matter; GM - gray matter; Vol - volume; SubCort - subcortical; ***y*** - CNS measured structure; y_0 - model constant member (intersect); b_i - regression coefficient of *i-th* variable *x*; *x* - regressed significant variable (e.g. Height, ICV, etc.); R^2 - coefficient of determination for the stepwise fitted model; R^2_{ICV} - coefficient of determination for fitted linear regression model utilizing sex, age and ICV variables; R^2_{Height} - coefficient of determination for fitted linear regression model utilizing sex, age and body height variables; r - Pearson correlation coefficient between measured ***y*** and stepwise model predicted ***y***; r_{ICV} - Pearson correlation coefficient between measured ***y*** and ICV; RMSE - root mean square error between measured ***y*** and stepwise model predicted ***y***; STD - standard deviation.
All variables listed in the fitted models met the statistical threshold condition p<0.05. In all cases the stepwise linear regression fitted model explained more data variance than concurrent linear mixture model utilizing sex, age and ICV (R^2_{ICV}); or sex, age and body height (R^2_{Height}), respectively. Coefficients of determination for other investigated mixture models are listed in the **Supplementary Table 3**. Pearson correlation coefficient also increased for the stepwise fitted model when compared to correlation levels with ICV (r_{ICV}) or body height (**Table 2**) separately.

**Fitted model: $y \propto y_0 + \sum b_i{*}x_i$**	R^2	R^2_{ICV}	R^2_{Height}	*r*	r_{ICV}	**RMSE**	***y (mean ± STD)***
BrainGMVol ∝ -17388 -1940*Age +3075*Height -960*Weight +0.195*ICV	60.4%	44.0%	19.9%	0.777	0.663	41233mm³	709752±64983mm³
BrainVol ∝ 96635 -2287*Age +3415*Height +0.407*ICV	64.1%	56.2%	16.7%	0.800	0.750	70360mm³	1283968±116414mm³
CorticalGMVol ∝ -89416 -1504*Age +2631*Height -838*Weight +0.154*ICV	58.0%	41.5%	20.1%	0.762	0.644	34957mm³	516698±53352mm³
CerebellumVol ∝ 123065 -628*Age +0.036*ICV	29.3%	22.1%	4.8%	0.539	0.470	12811mm³	160495±15217mm³
BrainStemVol ∝ 7880 +52.6*Height +0.0042*ICV	18.8%	15.7%	6.7%	0.433	0.396	2117mm³	23868±2350mm³
CorticalWMVol ∝ 19463 +1222*Height +0.179*ICV	47.6%	44.3%	9.8%	0.690	0.666	41271mm³	521861±56456mm³
SubCortGMVol ∝ 25010 +102*Height +0.014*ICV	38.8%	36.5%	8.3%	0.623	0.604	3987mm³	66076±5117mm³
PrecentralGMVol ∝ -2718 -104*Age +133*Height +0.0068*ICV	35.1%	21.2%	17.4%	0.592	0.461	2829mm³	28618±3456mm³
PostcentralGMVol ∝ -11060 +137*Height -48.4*Weight +0.0066*ICV	35.2%	25.4%	13.6%	0.593	0.504	2367mm³	20139±2903mm³
CSA-WM ∝ 16.59 +0.216*Height +1e-05*ICV	17.5%	11.7%	7.2%	0.418	0.304	6.3mm²	70.4±7.1mm²
CSA-SC ∝ 31.75 +0.184*Height +9e-06*ICV	10.4%	7.8%	4.2%	0.322	0.273	7.7mm²	79.2±8.1mm²
MD-SC-WM ∝ 0.934	0.0%	0.0%	0.8%	NaN	-0.038	$0.07{*}10^{-9}$ m²/s	$(0.93 \pm 0.13){*}10^{-9}$ m²/s
MTR-SC-WM ∝ 49.4 +0.07*Age -0.05*Weight	7.9%	3.7%	2.3%	0.286	-0.205	2.3%	47.4±3.5%
Cortical Thickness ∝ 2.57 -0.0035*Age	7.8%	0.4%	0.8%	0.277	0.064	0.08mm	2.46±0.08mm

Supplementary Table 5: Stepwise linear regression fitted models predicting CNS structural measure (y) for females.

Abbreviations: ICV - intracranial volume; CSA - cross-sectional area; SC - spinal cord; WM - white matter; GM - gray matter; Vol - volume; SubCort - subcortical; ***y*** - CNS measured structure; y_0 - model constant member (intersect); b_i - regression coefficient of *i-th* variable *x*; *x* - regressed significant variable (e.g. Height, ICV, etc.); R^2 - coefficient of determination for the stepwise fitted model; R^2_{ICV} - coefficient of determination for fitted linear regression model utilizing sex, age and ICV variables; R^2_{Height} - coefficient of determination for fitted linear regression model utilizing sex, age and body height variables; r - Pearson correlation coefficient between measured ***y*** and stepwise model predicted ***y***; r_{ICV} - Pearson correlation coefficient between measured ***y*** and ICV; RMSE - root mean square error between measured ***y*** and stepwise model predicted ***y***; STD - standard deviation.

All variables listed in the fitted models met the statistical threshold condition $p<0.05$. In all cases the stepwise linear regression fitted model explained more data variance than concurrent linear mixture model utilizing sex, age and ICV (R^2_{ICV}); or sex, age and body height (R^2_{Height}), respectively. Coefficients of determination for other investigated mixture models are listed in the **Supplementary Table 3**. Pearson correlation coefficient also increased for the stepwise fitted model when compared to correlation levels with ICV (r_{ICV}) or body height (**Table 2**) separately.

**Fitted model: $y \propto y_0 + \sum b_i * x_i$**	R^2	R^2_{ICV}	R^2_{Height}	***r***	r_{ICV}	**RMSE**	***y (mean ± STD)***
BrainGMVol ∝ 217777 -1844*Age +1991*Height +0.103*ICV	31.2%	17.0%	10.3%	0.558	0.412	41227mm^3	631351±49004mm^3
BrainVol ∝ 248425 +3301*Height +0.252*ICV	33.5%	27.2%	7.5%	0.579	0.522	72109mm^3	1131932±87622mm^3
CorticalGMVol ∝ 205975 -1926*Age +1176*Height +0.086*ICV	31.0%	17.6%	6.4%	0.557	0.420	34209mm^3	459995±40606mm^3
CerebellumVol ∝ -19376 +843*Height +0.016*ICV	21.1%	5.1%	16.9%	0.46	0.225	12555mm^3	141885±14008mm^3
BrainStemVol ∝ 5414 +95.2*Height	9.6%	2.1%	9.6%	0.31	0.145	1955mm^3	21142±2047mm^3
CorticalWMVol ∝ 287567 +0.126*ICV	25.7%	25.7%	2.5%	0.507	0.507	38207mm^3	456445±44114mm^3
SubCortGMVol ∝ 40128 +98.2*Weight +0.01*ICV	19.8%	15.2%	1.1%	0.445	0.389	3862mm^3	59363±4273mm^3
PrecentralGMVol ∝ 25312 -151*Age +0.0033*ICV	14.4%	5.3%	0.9%	0.379	0.230	2805mm^3	25326±3004mm^3
PostcentralGMVol ∝ 12888 +0.0039*ICV	8.4%	8.4%	1.5%	0.29	0.290	2287mm^3	18092±2378mm^3
CSA-WM ∝ 4.87 +0.307*Height +7e-06*ICV	14.0%	4.4%	8.1%	0.374	0.140	6.2mm^2	65.4±6.8mm^2
CSA-SC ∝ 14.41 +0.37*Height	11.6%	1.7%	10.2%	0.319	0.108	6.9mm^2	75.4±7.4mm^2
MD-SC-WM ∝ 0.962	0.0%	1.4%	0.7%	NaN	0.210	0.09*10^{-9} m^2/s	(0.96±0.12)*10^{-9} m^2/s
MTR-SC-WM ∝ 51.6 -0.063*Weight	9.6%	0.0%	0.4%	0.331	0.003	1.9%	47.5±3.9%
Cortical Thickness ∝ 2.58 -0.0042*Age	7.7%	0.0%	1.3%	0.278	0.004	0.09mm	2.46±0.09mm
