# Supplementary material for: Body size and intracranial volume interact with the structure of the central nervous system: A multi-center in vivo neuroimaging study
Source: Imaging Neurosci (Camb). 2025 May 7;3:imag_a_00559. doi: 10.1162/imag_a_00559 (PMC12319740; doi:10.1162/imag_a_00559)

Figure 1 – stable correlation coefficients

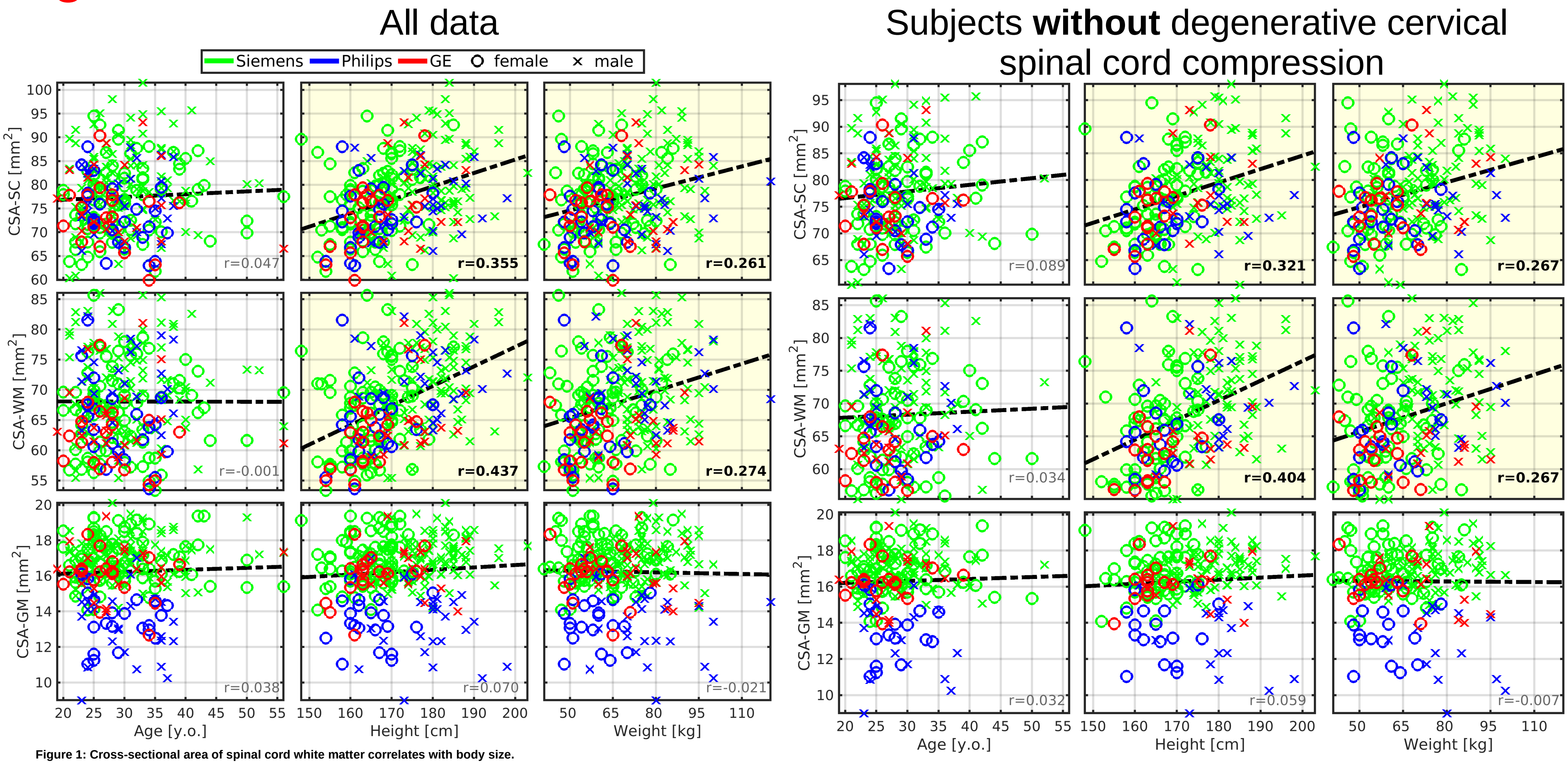

Figure 1: Cross-sectional area of spinal cord white matter correlates with body size.
Abbreviations: CSA - cross-sectional area; SC - spinal cord; WM - white matter; GM - gray matter; r - Pearson correlation coefficient. All spinal cord measurements were averaged from cervical C3-4 levels. Regression lines (i.e., the dashed black lines) were estimated from all available data points. Plots with statistically significant correlation (pFWE<0.05) are highlighted with yellow background, and corresponding r values are highlighted with black bold font.

Figure 1 – stable correlation coefficients

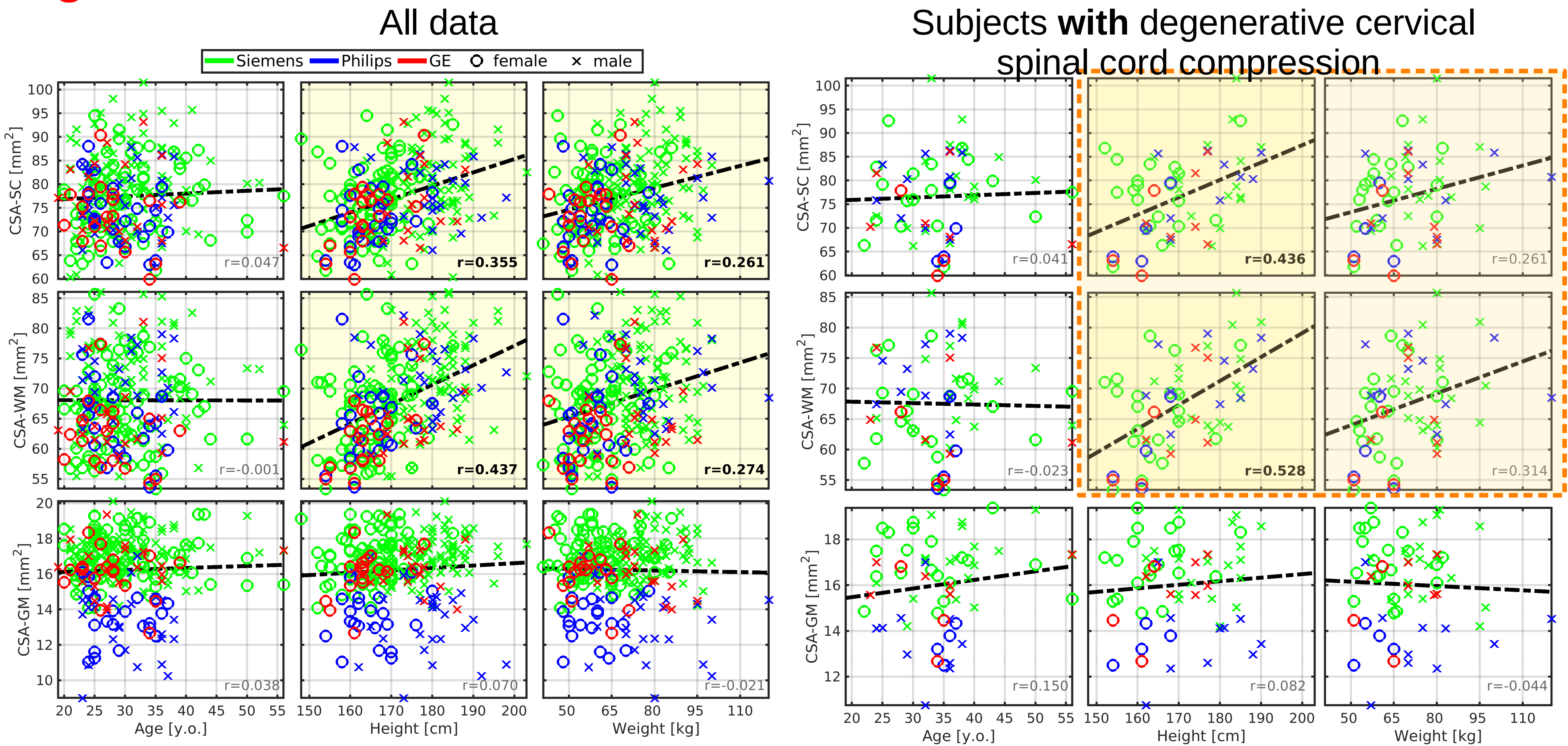

Figure 1: Cross-sectional area of spinal cord white matter correlates with body size.
Abbreviations: CSA - cross-sectional area; SC - spinal cord; WM - white matter; GM - gray matter; r - Pearson correlation coefficient. All spinal cord measurements were averaged from cervical C3-4 levels. Regression lines (i.e., the dashed black lines) were estimated from all available data points. Plots with statistically significant correlation (pFWE<0.05) are highlighted with yellow background, and corresponding r values are highlighted with black bold font.

Figure 3 – stable correlation coefficients

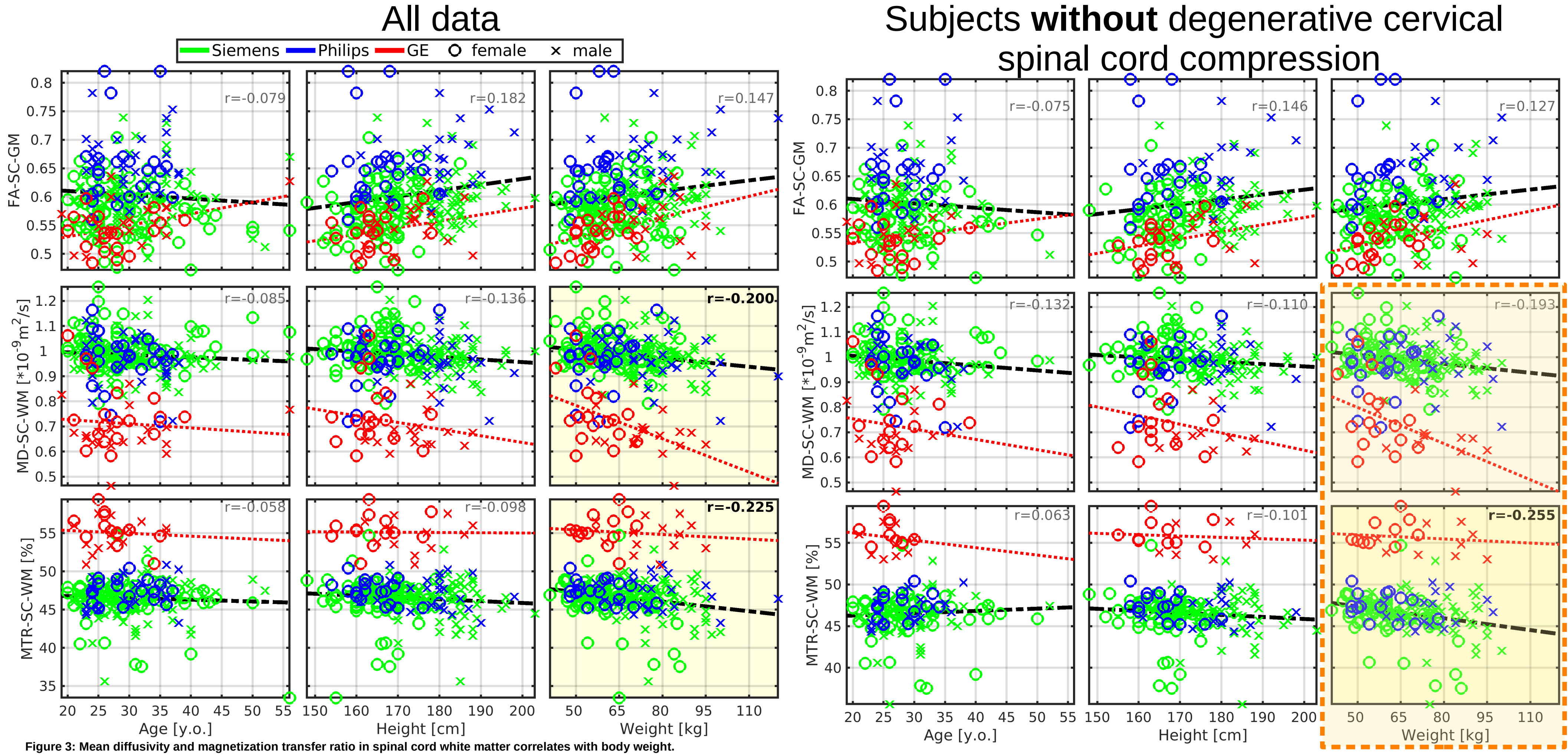

Figure 3: Mean diffusivity and magnetization transfer ratio in spinal cord white matter correlates with body weight.
Abbreviations: WM- white matter; MD - mean diffusivity; RD - radial diffusivity; MTR - magnetization transfer ratio; r - Pearson correlation coefficient. All spinal cord measurements were averaged from cervical C2-5 levels. Black dashed regression lines were estimated from the Siemens and Philips scanners' data points. Red dotted regression lines were estimated from the GE scanner's data points. Plots with statistically significant correlation (pFWE<0.05) are highlighted with yellow background, and corresponding r values are highlighted with black bold font.

Figure 3 – mostly stable correlation coefficients

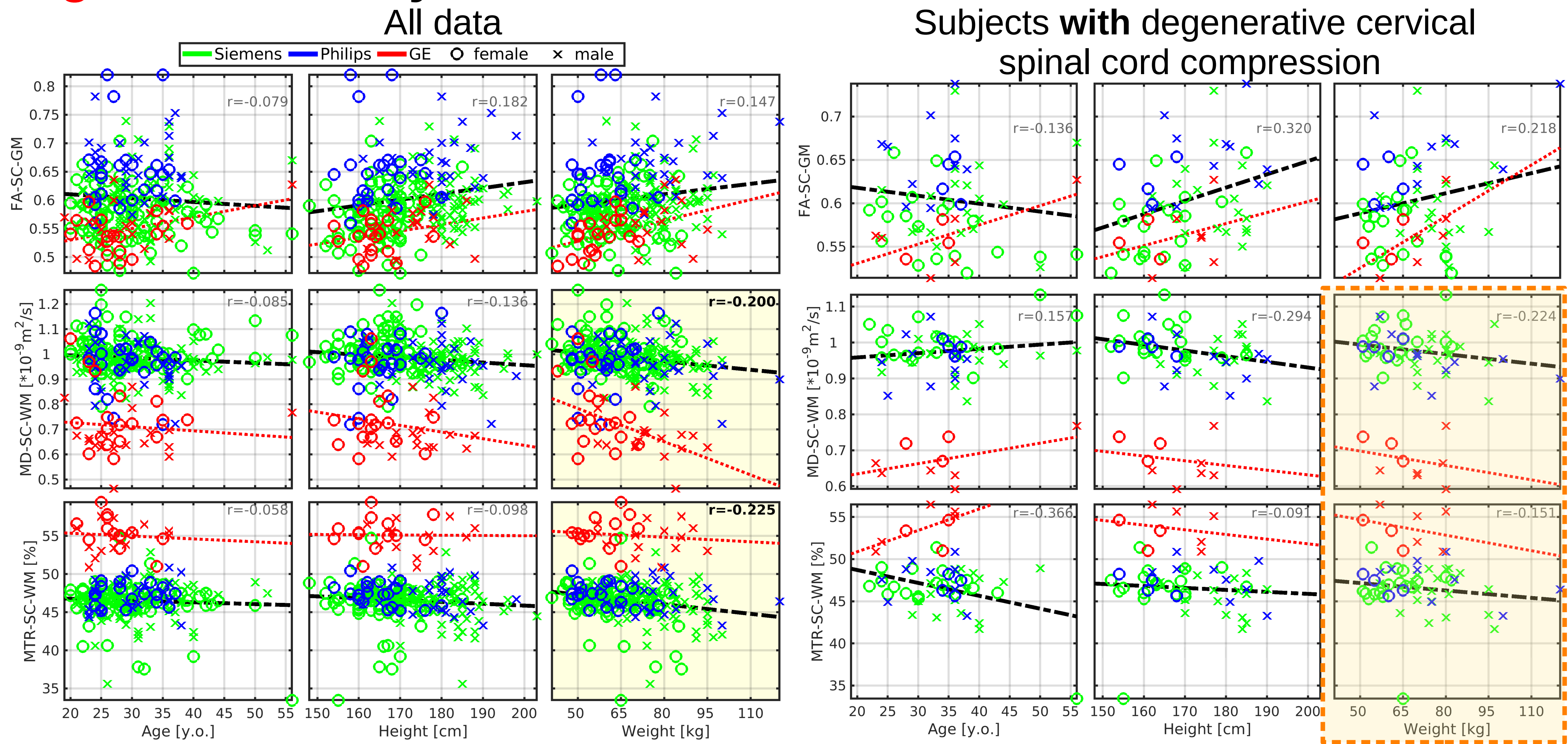

Figure 3: Mean diffusivity and magnetization transfer ratio in spinal cord white matter correlates with body weight.
Abbreviations: WM- white matter; MD - mean diffusivity; RD - radial diffusivity; MTR - magnetization transfer ratio; r - Pearson correlation coefficient. All spinal cord measurements were averaged from cervical C2-5 levels. Black dashed regression lines were estimated from the Siemens and Philips scanners' data points. Red dotted regression lines were estimated from the GE scanner's data points. Plots with statistically significant correlation (pFWE<0.05) are highlighted with yellow background, and corresponding r values are highlighted with black bold font.

Figure 5b – stable correlation coefficients

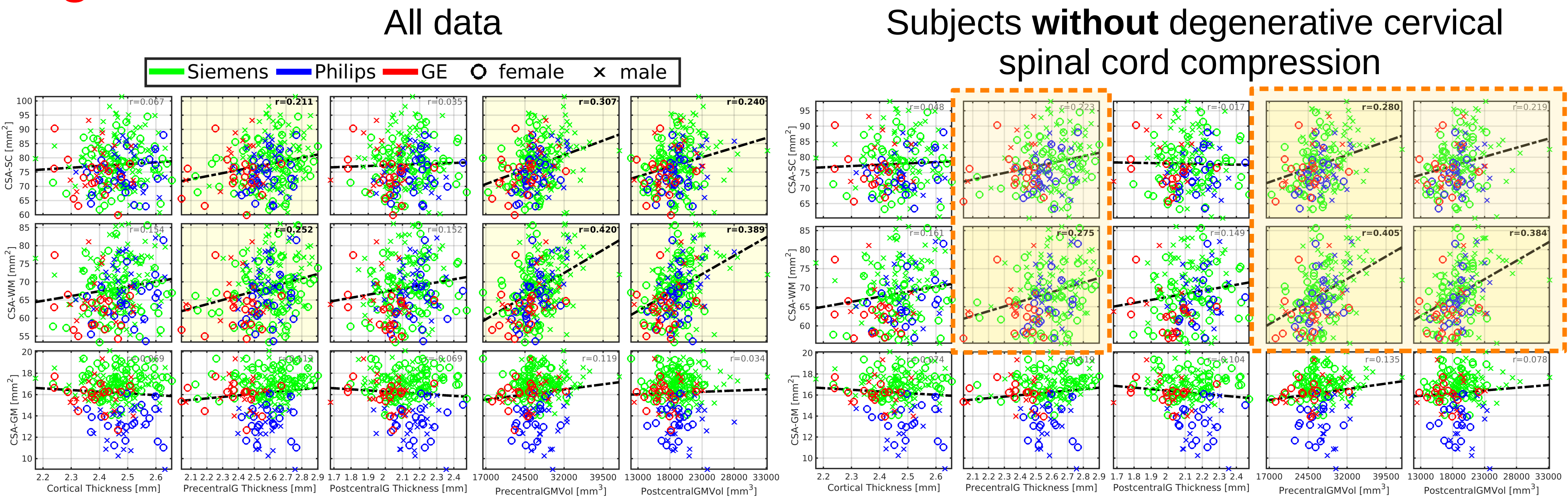

Figure 5: Cortical morphology correlates with body size, age, and cross-sectional area of the spinal cord white matter.
Abbreviations: CSA - cross-sectional area; SC - spinal cord; WM - white matter; GM - gray matter; PrecentralG - precentral gyrus; PostcentralG - postcentral gyrus; Vol - volume; r - Pearson correlation coefficient. Regression lines (i.e., the dashed black lines) were estimated from all available data points. Plots with statistically significant correlation (pFWE<0.05) are highlighted with yellow background, and corresponding r values are highlighted with black bold font. Graphs demonstrate correlation with CSA measured in the SC region as averages from cervical C3-4 levels.

Figure 5b – stable correlation coefficients

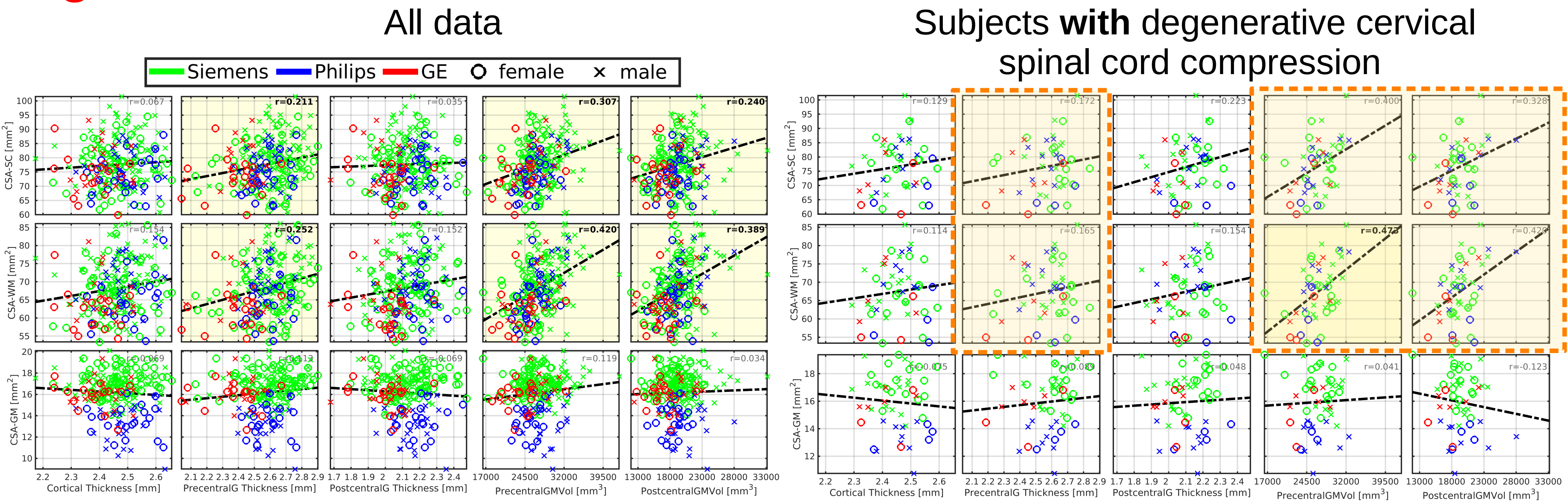

Figure 5: Cortical morphology correlates with body size, age, and cross-sectional area of the spinal cord white matter.
Abbreviations: CSA - cross-sectional area; SC - spinal cord; WM - white matter; GM - gray matter; PrecentralG - precentral gyrus; PostcentralG - postcentral gyrus; Vol - volume; r - Pearson correlation coefficient. Regression lines (i.e., the dashed black lines) were estimated from all available data points. Plots with statistically significant correlation (pFWE<0.05) are highlighted with yellow background, and corresponding r values are highlighted with black bold font. Graphs demonstrate correlation with CSA measured in the SC region as averages from cervical C3-4 levels.

Figure 6 – stable correlation coefficients

All data

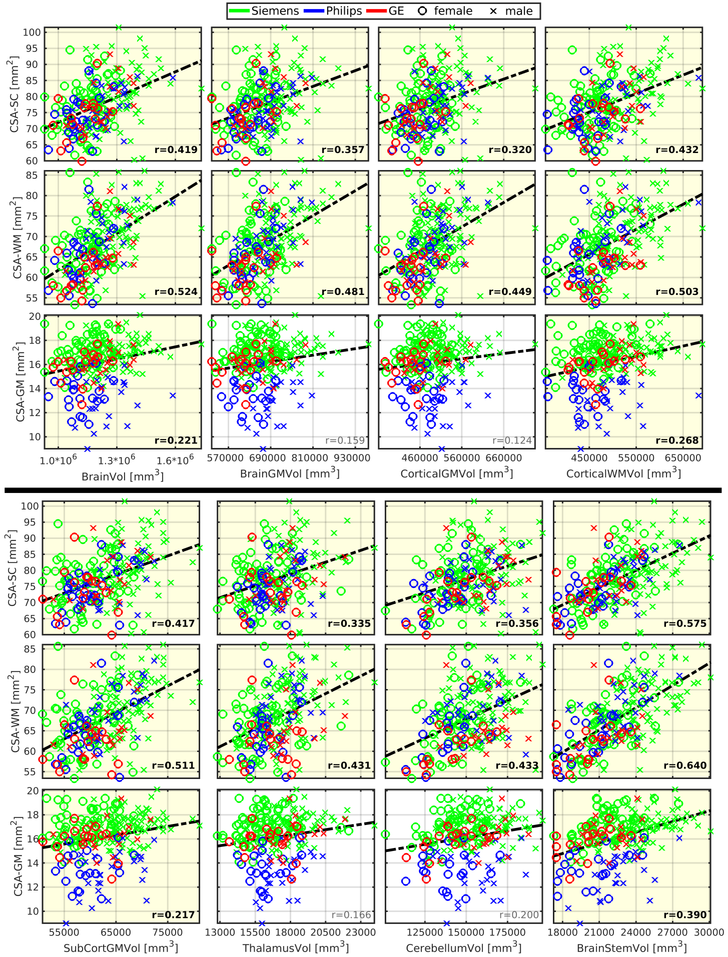

Subjects **without** degenerative cervical spinal cord compression

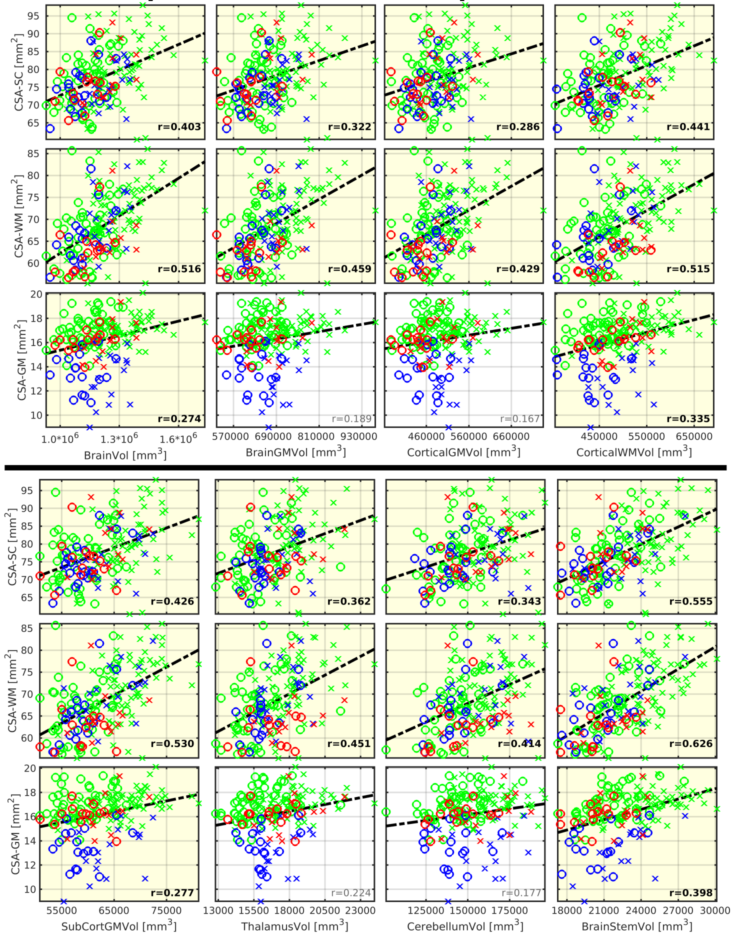

Figure 6: Brain morphology correlates with spinal cord morphology. Abbreviations: CSA - cross-sectional area; SC - spinal cord; WM - white matter; GM - gray matter; Vol - volume; SubCort - subcortical; r - Pearson correlation coefficient. All SC measurements were averaged from cervical C3-4 levels. Regression lines (i.e., the dashed black lines) were estimated from all available data points. Plots with statistically significant correlation (pFWE<0.05) are highlighted with yellow background, and corresponding r values are highlighted with black bold font.

Figure 6 – stable correlation coefficients

All data

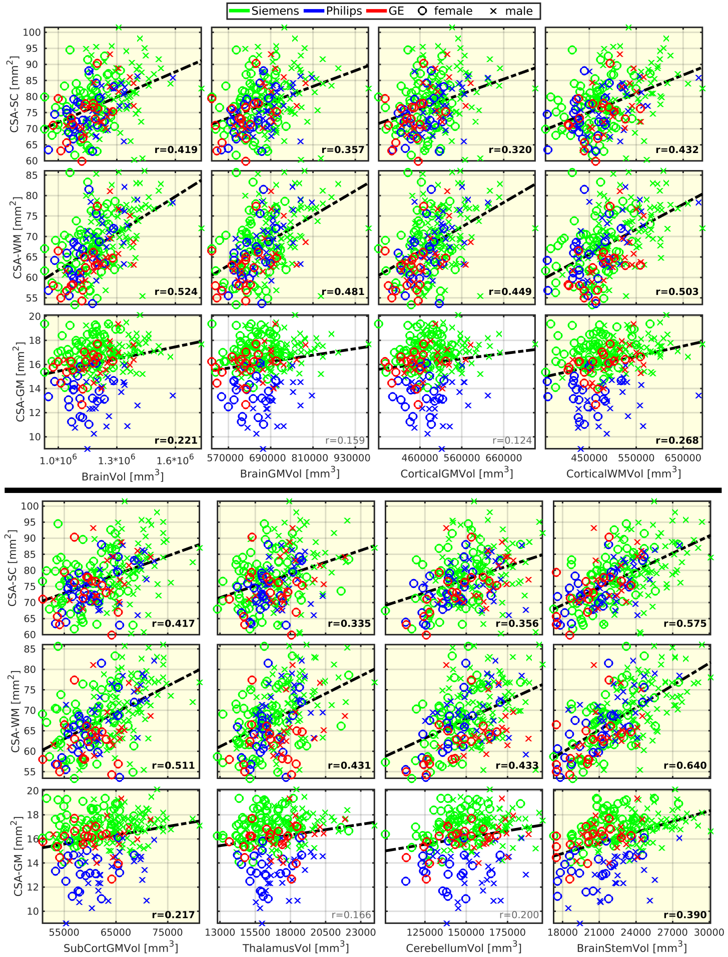

Figure 6: Brain morphology correlates with spinal cord morphology. Abbreviations: CSA - cross-sectional area; SC - spinal cord; WM - white matter; GM - gray matter; Vol - volume; SubCort - subcortical; r - Pearson correlation coefficient. All SC measurements were averaged from cervical C3-4 levels. Regression lines (i.e., the dashed black lines) were estimated from all available data points. Plots with statistically significant correlation (pFWE<0.05) are highlighted with yellow background, and corresponding r values are highlighted with black bold font.

Subjects **with** degenerative cervical spinal cord compression

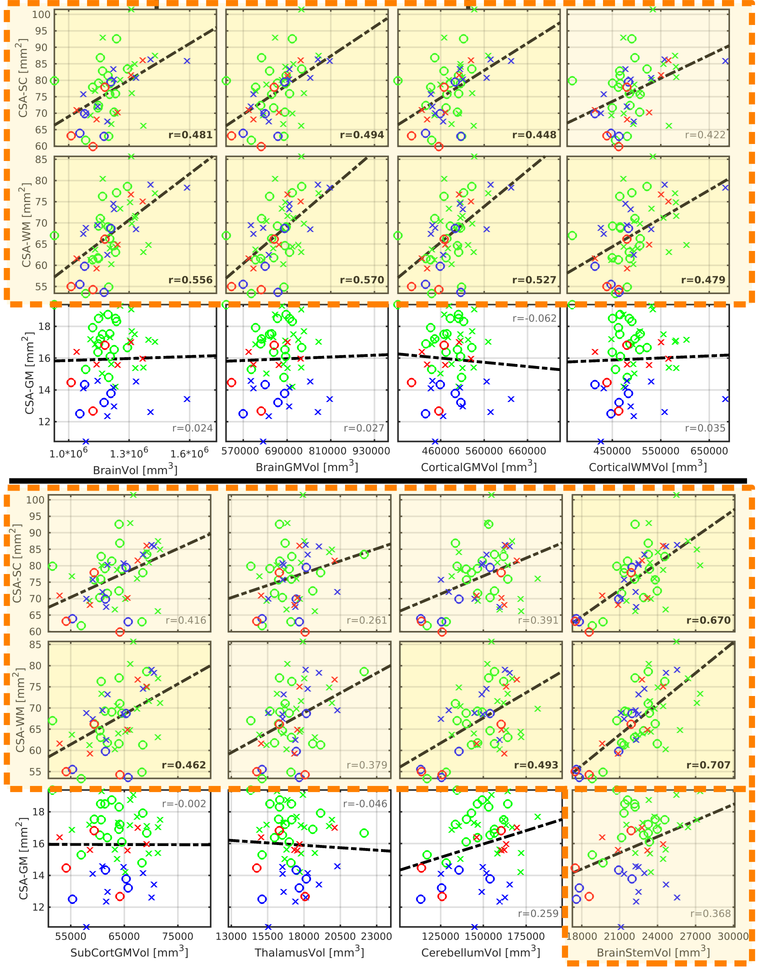

Supplement: Supplementary Slides [file imag_a_00559-supp_slides2.pdf]
